# Chloride, Alkoxide, or Silicon: The Bridging Ligand Dictates the Spin State in Dicobalt Expanded Pincer Complexes

**DOI:** 10.1021/acs.organomet.4c00374

**Published:** 2024-11-28

**Authors:** Roel L. M. Bienenmann, Arun S. Asundi, Martin Lutz, Ritimukta Sarangi, Daniël L. J. Broere

**Affiliations:** † Organic Chemistry and Catalysis, Institute for Sustainable and Circular Chemistry, Faculty of Science, Utrecht University, Universiteitsweg 99, 3584 CG Utrecht, The Netherlands; ‡ Stanford Synchrotron Radiation Lightsource, SLAC National Accelerator Laboratory, Stanford University, Menlo Park, California 94025, United States; § Structural Biochemistry, Bijvoet Centre for Biomolecular Research, Faculty of Science, Utrecht University, Universiteitsweg 99, 3584 CG Utrecht, The Netherlands

## Abstract

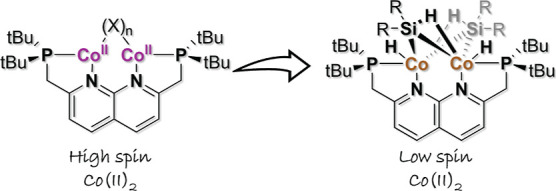

We report the synthesis and characterization of a series of high- and low-spin dicobalt complexes of the ^tBu^PNNP expanded pincer ligand. Reacting this dinucleating ligand in its neutral form with two equiv of CoCl_2_(tetrahydrofuran)_1.5_ yields a high-spin dicobalt complex featuring one Co inside and one Co outside of the dinucleating pocket. Performing the same reaction in the presence of two equivalents of KOtBu provides access to a high-spin dicobalt complex wherein both Co centers are bound within the PNNP pocket, and this complex also features a bridging OtBu ligand. Reacting either of the high-spin complexes with excess diethyl silane affords a low-spin dicobalt complex containing two unusual bridging Si-based ligands. These complexes were investigated using NMR spectroscopy, XAS, single crystal X-ray structure determination, and computational methods, showing that the Si-based ligands are best described as base-stabilized silylenes.

## Introduction

One of the major challenges in homogeneous catalysis is the use of first-row transition metals as catalysts.^1^ The reactivity of these metals is fundamentally different from that of their heavier and rarer noble metal counterparts that are currently often employed in catalysis. First-row transition metals tend to form high-spin complexes, for example, and they are typically more prone to 1e^–^ reactivity than noble metals.^1^ In nature, enzymes often employ multiple earth-abundant metals in one active site to facilitate catalysis using the cooperative action of the metal centers.^2−7^ This sparked the growing interest in using complexes with multiple first-row metal atoms in close proximity to control their reactivity by using this metal–metal cooperativity (MMC).^1,3,6−10^ The area of MMC in coordination chemistry is still relatively underdeveloped; hence, it is important to gain a better fundamental understanding of this phenomenon and the effect that it has on the reactivity of metal complexes.

**Scheme 1:**
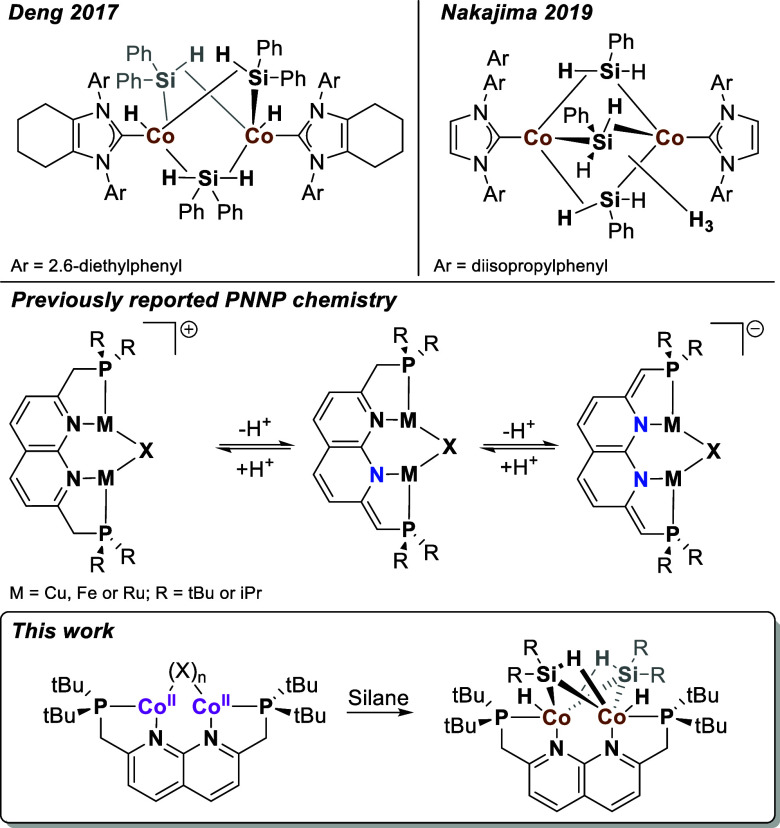
Previously Reported Dicobalt Silane Complexes by the Groups of Deng and Nakajima (Top);^16,17^ In the Latter Structure, the Positions of Three of the Hydrides in the Co_2_Si_3_ Core are Unclear. PNNP Ligand Protonation and Deprotonation Reactions Reported Previously by Our Group (Middle);^18−23^ A Representation of the Work Detailed in This Study (Bottom)

A class of dinuclear complexes that have been studied extensively for catalysis in the past are dinuclear metal carbonyl complexes such as Fe_2_(CO)_9_ and Co_2_(CO)_8_.^11^ For example, Co_2_(CO)_8_ is used as a catalyst for hydroformylation industrially.^12,13^ In addition, it has also been shown to perform reduction reactions such as hydrosilylation.^14,15^ However, even though Co_2_(CO)_8_ is a dinuclear precatalyst, the important intermediates that are found in the hydrosilylation mechanisms of this compound are mononuclear.^15^ There are few examples in the literature of well-defined dicobalt silane complexes, even though these are of interest for studying their relevance in catalytic reduction reactions. Two examples of work on dicobalt complexes were reported by the groups of Nakajima and Deng.^16,17^ They show that low-valent cobalt complexes supported by monodentate *N*-heterocyclic carbene ligands can react with silanes to form dinuclear cobalt silane complexes. These complexes feature interesting bridging silane ligands, which can be described as a halted oxidative addition of the Si–H bonds (Scheme 1).

To study the coordination chemistry and reactivity of dinuclear complexes, our group has developed the PNNP expanded pincer ligand system.^19^ This naphthyridine-based ligand can tightly bind two metal atoms in close proximity, which allows for potential MMC in well-defined dinuclear complexes. Previously, this ligand has been used to make copper,^18−20^ iron,^22^ and ruthenium^21,23^ complexes (Scheme 1). Motivated by our interest in further exploring the potential of the PNNP ligand with other first-row transition metals, especially those that readily adopt high-spin states, we here report the coordination chemistry of the PNNP ligand with cobalt. We disclose the synthesis and reactivity of two high-spin dinuclear cobalt(II) complexes and investigate their reactivity with silanes. In addition, two low-spin dinuclear cobalt complexes featuring unusual bridging Si-based ligands are investigated.

## Results and Discussion

### Synthesis of High-Spin Dicobalt Complexes

**Scheme 2:**
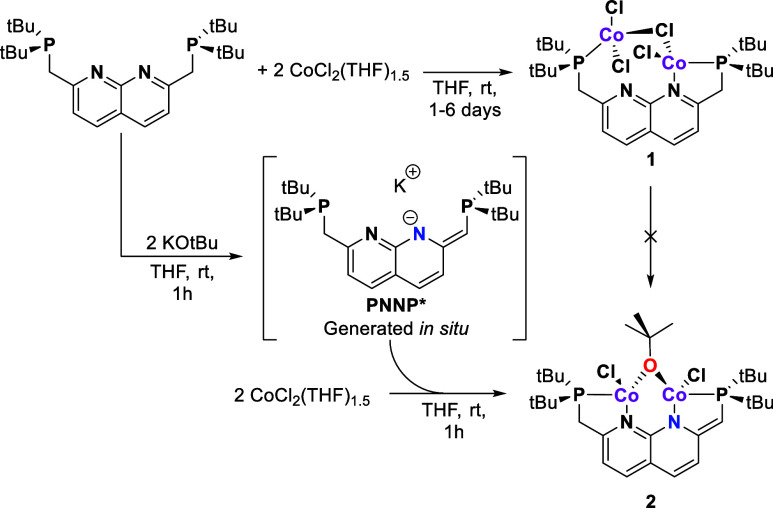
Synthesis of Complexes **1** and **2**

Attempts to directly synthesize low-valent dinuclear cobalt complexes by reacting the PNNP ligand^19^ with low-valent cobalt(I) precursors such as CoCl(PPh_3_)_3_ and Co(PMe_3_)_4_^24^ led to intractable mixtures. Therefore, we decided to attempt the synthesis of a cobalt(II) parent compound, which could then be reduced to obtain the desired low-valent cobalt species. The reaction of 1 eq ^tBu^PNNP ligand with 2 eq of CoCl_2_(THF)_1.5_ (Scheme 2) in tetrahydrofuran (THF) led to the formation of a bright blue paramagnetic precipitate, which was characterized as complex **1** and isolated in 99% yield. Complex **1** does not dissolve in ethereal or apolar solvents but readily dissolves in dichloromethane (DCM). The paramagnetic ^1^H NMR spectrum of **1** in DCM-*d*_2_ at 298 K features broad peaks in the range of 118.47 to −33.47 ppm. There are 7 distinguishable peaks that integrate to one proton each, indicating a nonsymmetric complex.^25^ There are two signals integrating to ∼9 protons and one that integrates to 18 protons, meaning that two of the *t*Bu-groups of the ligand are magnetically inequivalent. This is consistent with the observation of more than 6 peaks for the naphthyridine backbone, and it indicates that both the *C*_2_ and *C*_*S*_ symmetries of the ligand are no longer present in complex **1**.

**Figure 1:**
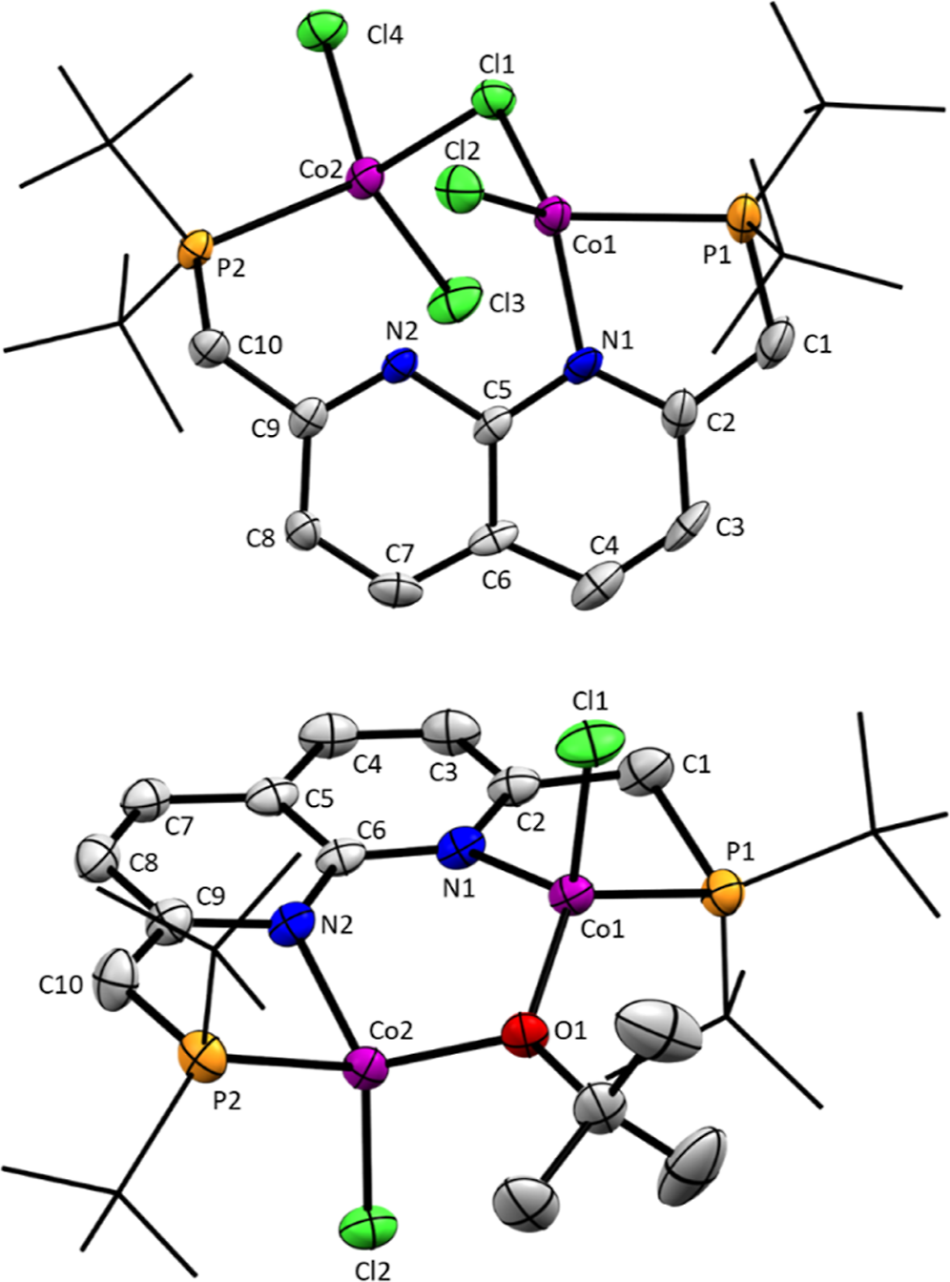
Solid state structures of complex **1** (top) and of complex **2** (bottom). Ellipsoids are drawn at 50% probability, H atoms are omitted, and the *t*Bu-groups on the phosphines are shown in wireframe for clarity. In the structure of **1**, one disordered DCM molecule was omitted for clarity.

Crystals of **1**, suitable for single crystal X-ray diffraction (scXRD), were grown by vapor diffusion of diethyl ether into a DCM solution of **1**. The solid-state structure of **1** (Figure 1) features two different cobalt centers; one is bound in the PN binding pocket, and one is only bound to the phosphine of the second binding pocket. Both cobalt centers have a tetrahedral geometry. The structure of **1** features one bridging chloride atom and three terminal ones. Due to one of the cobalt atoms binding outside of the PNNP pocket, the Co–Co distance of 3.8838(17) Å is larger than what is normally observed for PNNP complexes in which both metals are bound within the dinucleating binding pocket.^18,19^ The Co1–Cl1–Co2 angle of 113.07(10)° is indicative of the chloride binding as an LX type ligand to the cobalt centers. The Co–Cl1 bond distances (Table 1) are also significantly longer than the Co–Cl bonds for the terminal chlorides, consistent with this LX-type binding description. The C1–C2 and C9–C10 bond lengths of 1.486(13) and 1.505(11) Å are in line with those of C–C single bonds, which is indicative of a neutral PNNP backbone. The solid-state structure of **1** is consistent with the observed low symmetry in the paramagnetic ^1^H NMR spectrum. This suggests that the structure in solution is similar to that observed in the solid state.

**Table 1:** Selected Bond Distances and (Torsion) Angles of Compounds **1** and **2**, in Å and °, Respectively

Atoms	**1**	**2**
Co1–Co2	3.8838(17)	3.0710(5)
Co1–Cl1	2.288(2)	2.2347(8)
Co1–Cl2	2.220(3)	-
Co2–Cl1	2.367(3)	-
Co2–Cl2	-	2.2344(8)
Co2–Cl3	2.237(3)	-
Co2–Cl4	2.257(2)	-
Co1–O1	-	1.9093(17)
Co2–O1	-	1.9246(19)
Co1–N1	2.010(7)	2.057(2)
Co2–N2	3.231(6)	2.051(2)
Co1–P1	2.405(3)	2.3868(8)
Co1–P2	2.425(3)	2.3688(8)
C1–C2	1.486(13)	1.493(4)
C9–C10	1.505(11)	1.375(4)
P1–C1–C2	113.4(6)	114.0(2)
P2–C10–C9	114.8(6)	120.1(2)
Co1–Cl1–Co2	113.07(10)	-
Co1–O1–Co2	-	106.46(8)
P1–C1–C2–N1	22.9(11)	30.3(3)
P2–C10–C9–N2	63.1(9)	1.4(4)

Various attempts to reduce **1** with reducing agents such as KC_8_ and NaHBEt_3_ led to the formation of intractable mixtures. Similarly, attempts to deprotonate **1** with strong bases were also unsuccessful. We hypothesize that the reduction and deprotonation of **1** is hampered by its insolubility in non-halogenated solvents, resulting in detrimental side reactions with the more soluble reaction products that are exposed to a large excess of reductant or base. To overcome this, we changed the order and first deprotonated the PNNP ligand in situ and then added this deprotonated ligand to the cobalt precursor. The deprotonation of the PNNP ligand with KH to form the PNNP* anion and the subsequent complexation with CoCl_2_(THF)_1.5_ led to the formation of a CoCl_2_ THF coordination polymer and not to a complex containing the ligand.^26^ Inspired by our group’s dicopper PNNP chemistry,^19^ we hypothesized that performing the complexation of the PNNP* anionic ligand in the presence of an equivalent of KOtBu may lead to a OtBu-bridged complex. Therefore, 2 eq of KOtBu were added to the PNNP ligand^27^ in THF, leading to a quick color change to deep red, indicative of the formation of the partially dearomatized PNNP* anion (Scheme 2).^28^ The addition of this deep red mixture to a blue THF solution of CoCl_2_(THF)_1.5_ resulted in a brown/red mixture from which paramagnetic complex **2** was isolated in 74% yield. The ^1^H NMR spectrum of **2** in C_6_D_6_ at 298 K features much sharper resonances than **1** in the range of 197 to −2.6 ppm.

From the 12 resonances in the ^1^H NMR spectrum of complex **2**, 5 integrate to 9H each, indicating 5 magnetically inequivalent tBu-groups. The other 7 resonances integrate to 1H, consistent with the presence of 7 magnetically inequivalent protons on the ligand backbone. The inequivalence of the tBu-groups as well as the presence of two signals for the methylene linker in **2** is indicative of a nonsymmetric complex similar to **1**.

Crystals of **2** suitable for scXRD were obtained by slow vapor diffusion of hexane into a toluene solution of the complex. The solid-state structure of **2** (Figure 1) revealed the presence of a bridging OtBu ligand consistent with the NMR spectra. The C1–C2 bond length of 1.493(4)Å is consistent with a C–C single bond and of similar length as the C–C bonds of the methylene linkers in **1**. The C9–C10 bond length of 1.375(4) in **2** is significantly shorter than the C1–C2 bond length, indicative of double bond character, which is consistent with a partially dearomatized naphthyridine backbone. This is further supported by the observed P1–C1–C2 and P2–C10–C9 angles, which are 114.0(2) and 120.1(2)° and hence consistent with sp^3^ and sp^2^ hybridization of the bridging carbon, respectively. The Co1–O–Co2 angle of 106.46(8)° is indicative of the *tert*-butoxide ligand binding in an LX-type fashion to the cobalt centers, similar to the bridging chloride in **1**. The Co–Co distance in **2** is 0.8128(18) Å shorter than that in **1** since both cobalt atoms are in the PNNP binding pocket in this structure. Compared to other PNNP complexes, however, the M–M distance of 3.0710(5) in **2** is still rather large.^18−20,29^ This seems to be due to the bridging *tert*-butoxide ligand that is bound in an LX-type fashion, since the relatively large ideal M–L–M angle of 109.5° in such a binding mode forces a larger M–M separation than what would be expected for a three-center two-electron (3c-2e) bound bridging ligand, for example. This was also observed for the M–M distance in similar PNNPCu_2_OtBu complexes (2.9259(6) to 3.0467(2)Å), which feature significantly larger M–M distances compared to dicopper PNNP complexes with 3c-2e bound bridging ligands (2.3870(6) to 2.5122(6) Å).^18−20,29^ The cobalt centers in **2** display distorted tetrahedral geometries and are located slightly above and below the naphthyridine plane, respectively. This is enabled by the flexibility in the phosphine arms, which accommodate the preferred bond angles in combination with the LX-type binding of the *tert*-butoxide ligand. The solid-state structure is consistent with the lack of symmetry observed in the ^1^H NMR spectra. The chloride atoms that point in different directions on Co1 and Co2 break the *C*_*s*_ symmetry, and the *C*_2_ symmetry of the ligand is broken by the partially dearomatized backbone. This accounts for the fact that each *t*Bu group and backbone proton is magnetically inequivalent.

### Reducing Compound **2**

**Scheme 3:**
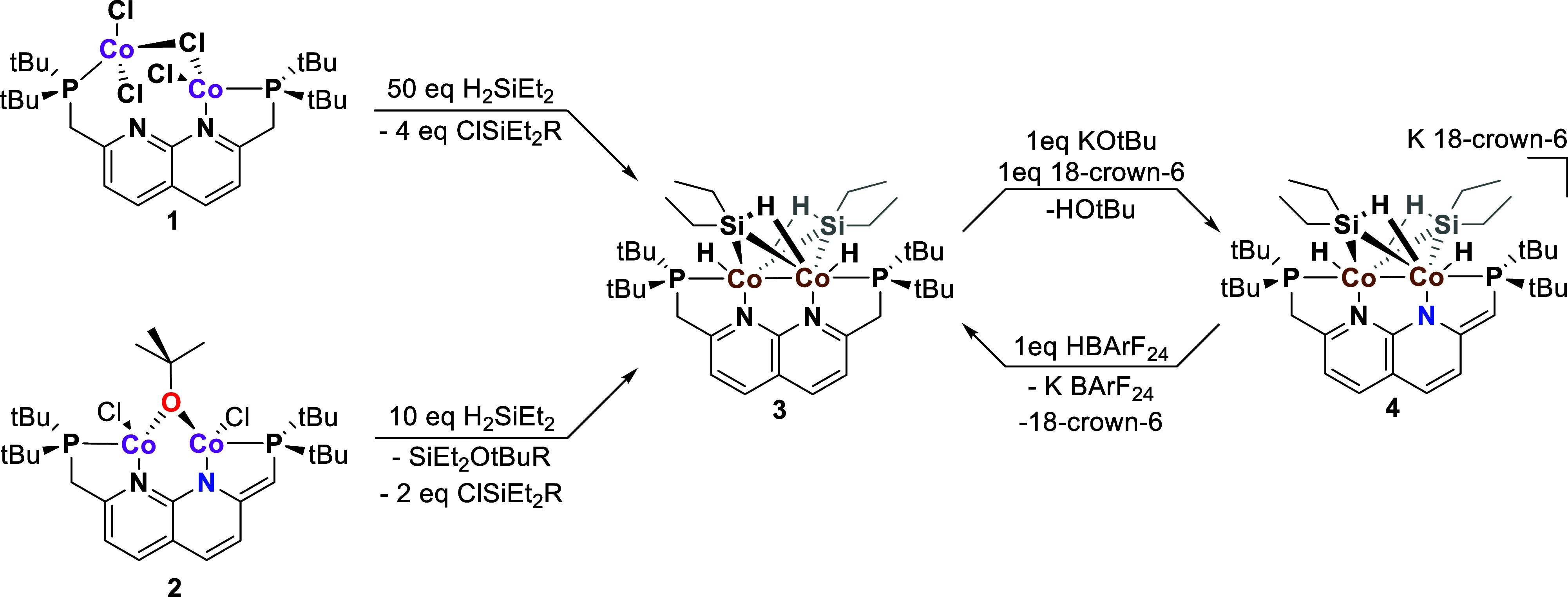
Synthesis of Complexes **3** and **4** from Complexes **1** and **2** as Starting Materials R = H or Cl, the exact composition of silane biproducts was not determined

Taking inspiration from the related copper complexes,^19^ we reacted **2** with a silane with the goal to substitute the *tert*-butoxide for a hydride ligand. Complex **2** was reacted with excess diethylsilane at 90 °C during which the color changed from red/brown to dark brown/black. From this mixture, complex **3** was isolated as an air- and moisture-sensitive black solid (Scheme 3). Alternatively, **3** can be synthesized from **1** by reacting it with diethylsilane at 110 °C, which is a more robust method. In this case, **3** was isolated as a black crystalline solid in 76% yield. Surprisingly, complex **3** was diamagnetic in contrast to **1** and **2**. The ^31^P NMR spectrum of **3** in C_6_D_6_ at 298 K features one broad resonance at 117 ppm indicative of a *C*_2*V*_ symmetric complex. The ^1^H NMR spectrum of **3** in C_6_D_6_ at 298 K shows a broad doublet at −13.74 ppm that integrates to 4H, indicative of four hydrides coupling to the phosphines of the PNNP ligand. The two doublets at 7.00 and 6.37 ppm originating from the naphthyridine backbone and one doublet at 2.75 ppm from the methylene linkers also indicate a *C*_2*V*_ symmetric complex. In the aliphatic region, there is one doublet at 1.30 ppm coupling to phosphorus (^3^*J*_H–P_ = 11.9 Hz) for the tBu-groups and three signals (m, 1.47 ppm, 10H; t, 1.10 ppm, 6H; q, 0.80 ppm, 4H) for the ethyl groups on the silicon atoms, indicating two magnetically inequivalent ethyl groups. Complex **2** also shows a ^29^Si resonance at 136 ppm,^30^ a region in which typically (base-stabilized) silylenes are found.^31,32^

**Figure 2:**
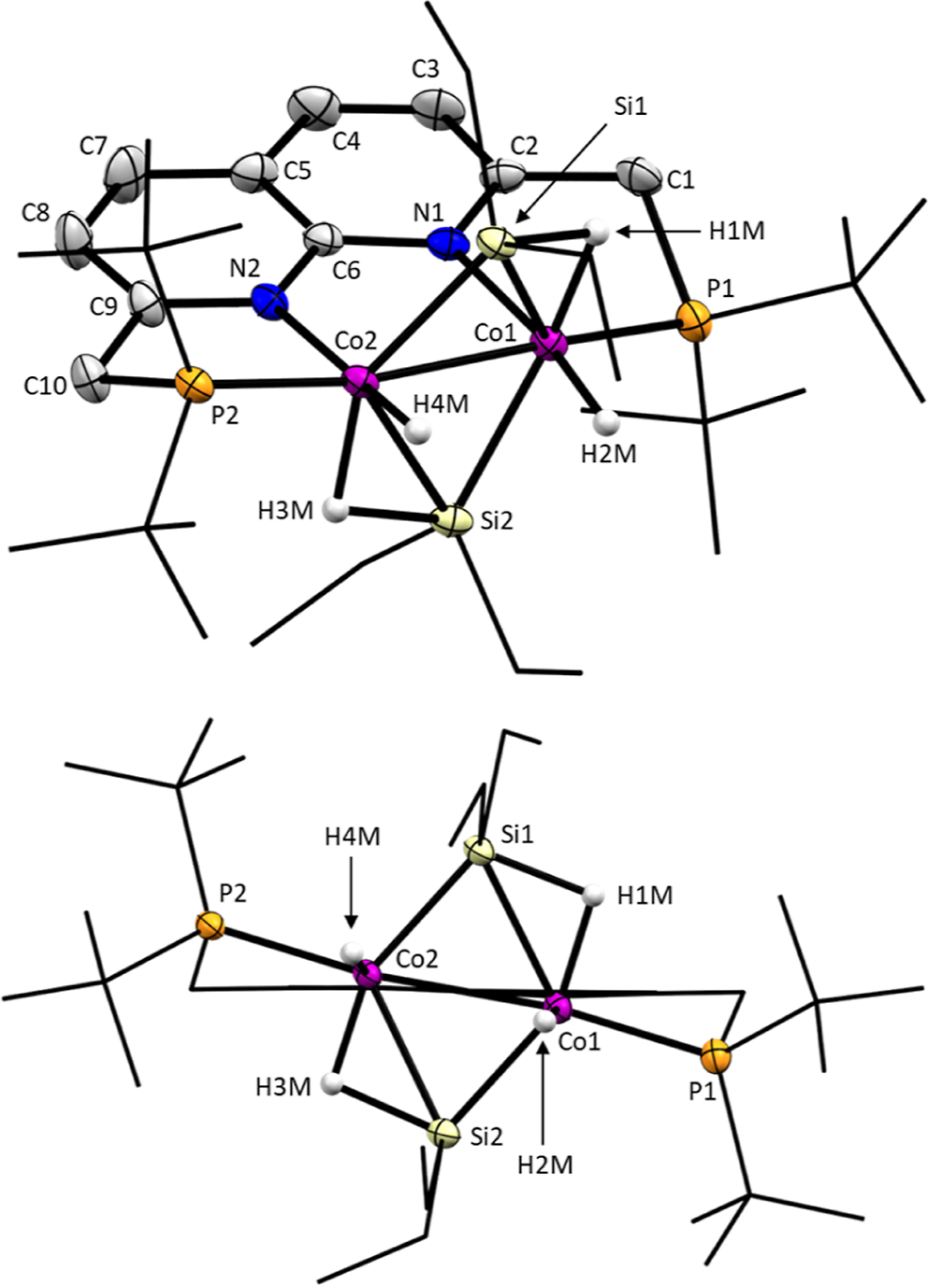
Solid-state structure of **3** from two perspectives. Ellipsoids are drawn at 50% probability and H-atoms (except the hydrides) were omitted. The *t*Bu and Et groups are drawn in wireframe (top and bottom) and the naphthyridine is drawn in wireframe (bottom only) for clarity. Selected distances (Å) and torsion angles (deg): C1–C2,1.496(5), C10–C9 1.504(4), Co2–Co1 2.5172(6), Co2–Si1 2.2263(9), Co1–Si1 2.3261(10), Co2–Si2 2.3325(10), Si2–Co1 2.2276(9), Co1–P1 2.1563(9), P2–Co2 2.1543(9), Co2–N2 1.954(3), Co1–N1 1.963(3), Co1–Si1–Co2 67.09(3), Co1–Si2–Co2 66.96(3), P1–C1–C2 109.3(2), P2–C10–C9 109.5(2), P1–Co1–Co2 170.88(3), P2–Co2–Co1 171.40(3), P1–C1–C2–N1 27.6(4), and P2–C10–C9–N2 28.6(4).

**Scheme 4:**
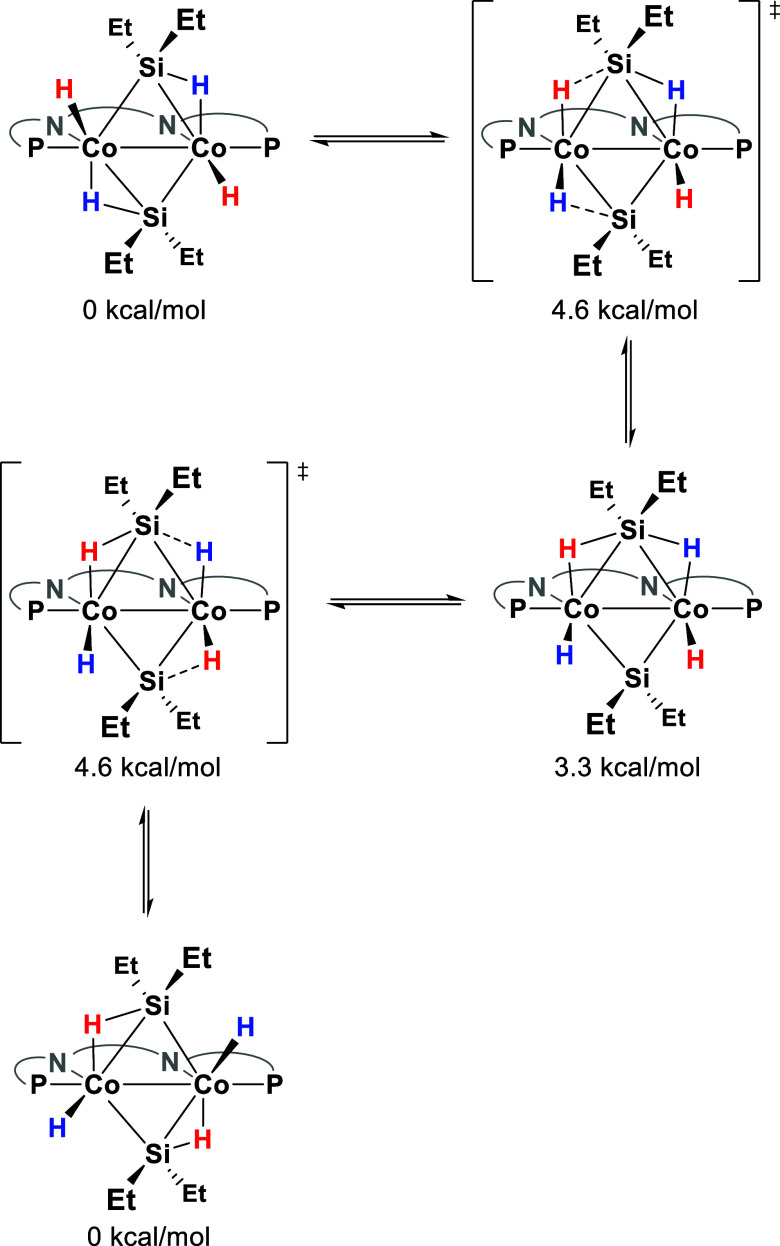
Proposed Rapid Equilibrium Responsible for the Observed *C*_2*v*_ Symmetry in NMR for Complex **3** Based on DFT Calculations The naphthyridine backbone is shown schematically for clarity.

Crystals of **3** suitable for X-ray diffraction were obtained by the slow vapor diffusion of hexane into a benzene solution of the complex. The solid-state structure of **3** (Figure 2) features two silicon atoms bridging the two cobalt centers; however, not symmetrically. The Si1–Co2 and Si2–Co1 distances are significantly shorter by ∼0.10 Å than the Si1–Co1 and Si2–Co2 distances. This asymmetry is also visible in the hydride ligands that were located in the difference Fourier maps, whose positions are consistent with the density functional theory (DFT) optimized structure. Two hydrides (H1M and H3M) are bridging between cobalt and silicon, and two hydrides (H2M and H4M) are bound terminally to cobalt, despite there being only one hydride signal in NMR. This difference between the solid-state and solution can be rationalized through a rapid equilibrium between the two sets of hydrides (Scheme 4) on the NMR time scale due to which their resonances average out. This also explains why in NMR a *C*_2*v*_ symmetric species is observed in contrast to the approximate *C*_2_ symmetry observed in the solid state. We attempted to observe the decoalescence of the hydride peaks using VT NMR; however, even at −70 °C no decoalescence was observed. The feasibility of this equilibrium was also evaluated with DFT calculations, which showed that the energy barrier associated with this equilibrium is 4.6 kcal/mol, indicating that this process should be very rapid both at room temperature and at −70 °C. The silicon atoms in **3** bind the complex in such a way that one of the ethyl groups points toward the backbone and one in the direction of the hydrides (H2M and H4M). This explains the observation of two magnetically inequivalent ethyl groups in the ^1^H NMR spectrum, as discussed previously. It also shows that the orientation of the ethyl groups is retained in solution, implying the absence of fast equilibria equilibrating the ethyl groups on the NMR time scale. This supports the idea that only the hydrides move due to an equilibrium, as proposed in Scheme 4, as opposed to an equilibration mechanism, which would involve dissociation of the silicon groups.

The Co1–Co2 distance in the solid-state is 2.5172(6) Å, which is well within the sum of van der Waals radii for cobalt and also within the range of M–M distances that have been observed before in PNNP complexes.^33^ It is, however, fairly long compared to known complexes which feature a Co–Co bond, especially considering that the current consensus in the field is that, in Co_2_(CO)_8_ (Co–Co 2.52 Å), such a bond is not present, despite what was long believed.^34^ In the literature, conclusions on Co–Co bonding (or lack thereof) for similar complexes are based mainly on the metal–metal distance.^16,17^ However, in our experience with dicopper PNNP complexes, the metal–metal distance is not an ideal indication for metal–metal bonding in these complexes.^29^ An in-depth study on the Co–Co bonding in **3** using X-ray absorption spectroscopy is reported in a separate study, in which this question is addressed both experimentally and with time-dependent density-functional theory calculations.^35^ From that work, we were able to conclude that there is a direct Co–Co bond present in complex **3** (and also in complex **4**, see below) and also that cobalt is in its 2+ oxidation state in these complexes.

**Scheme 5:**
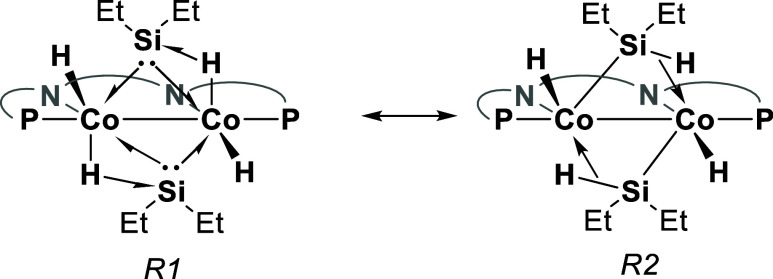
Two main resonance structures describing the core of **3**, shown using the 3c-2e bond notation proposed by Parkin et al.^36^ The naphthyridine backbone is shown schematically for clarity.

The sum of angles around the silicon atoms with the two ethyl groups and a centroid in the middle of the two cobalt centers is, on average, 359.9°. Together with the chemical shift of these nuclei in ^29^Si NMR, this suggests that these silicon groups have significant silylene character (*R*1, Scheme 5). Although the positions of H1M and H3M in the solid-state structure imply the presence of some silyl character as well (*R*2, Scheme 5). Based on the experimental data and the electronic structure calculations (see Supporting Information), we conclude that this is best described as a base-stabilized silylene structure in which H1M and H3M act as bases to stabilize the empty p-orbital on the silylene moieties and hence lead to the partial silyl character that is observed. The base-stabilized silylene description is in essence a combination of *R*1 and *R*2 shown in Scheme 5, meaning that silicon possesses partial silylene and partial silyl character.

Something to note is that complex **3** features a neutral naphthyridine backbone, as evidenced by the C1–C2 and C9–C10 distances of 1.496(5) and 1.504(4) Å, similar to what was observed in complex **1**. Considering that complex **3** can be synthesized from complex **2** using only diethyl silane (Scheme 3), this implies that one of the protons on the methylene linker originates from silane in this case. It is not completely clear what the mechanism of the formation of **3** is; however, it seems possible that at some point a Co–H intermediate is formed in which the hydride is sufficiently acidic to protonate the backbone. Alternatively, a radical reaction in which a H atom is transferred from cobalt to the backbone is also possible.^37^

### Synthesis of Complex **4**

Complex **3** is a very stable molecule under an N_2_ atmosphere, as can already be deduced from the harsh conditions under which it is synthesized. Since the NMR analysis and solid-state structure of **3** implied that the silicon moieties are silylenes, they would be expected to react as electrophiles, especially toward alkoxide bases. When 1 equiv of KOtBu with 1 equiv of 18-crown-6 was added to complex **3**, a color change from black/brown to dark green was observed, indicative for the formation of a new complex (**4**), which was isolated as a dark green air- and moisture-sensitive solid in 77% yield. To our surprise, the NMR spectra of **4** are quite similar to those of **3**. The ^1^H NMR spectrum features two broad overlapping doublets in the hydride region of the spectrum which integrate to four protons in total similar to **3**, however, it is shifted upfield to −15.77 ppm. In addition, the naphthyridine backbone of **4** shows four doublet resonances, indicative of a lower symmetry than that for complex **3**. There is one peak at 3.09 ppm belonging to the methylene linker of **4** and one at 4.26 ppm which belongs to a methine linker. This shows that in this reaction, the backbone was deprotonated instead of there being a reaction between the alkoxide base and the silylene fragment. When 1 eq of HBArF_24_ is added to complex **4**, complex **3** is formed again, showing that the deprotonation is reversible, which supports the structural composition of **4**, as depicted in Scheme 3. In addition, the structure of **4** was probed with the extended X-ray absorption fine structure (EXAFS) (see below).

To better understand why KOtBu deprotonates **3** instead of reacting with the silane groups, we decided to probe whether this preference is due to an electronic effect or whether sterics may kinetically block a reaction on silicon. Therefore, we calculated the *f*_*n*+1_ Fukui function (Figure S28) that shows that there is an electrophilic site on two of the methylene protons, while there is no contribution of the Fukui function visible on silicon. This indicates that the lack of reactivity has an electronic origin and is likely caused by the effective stabilization of the silylene by hydrides.

### X-ray Absorption Spectroscopy

**Figure 3:**
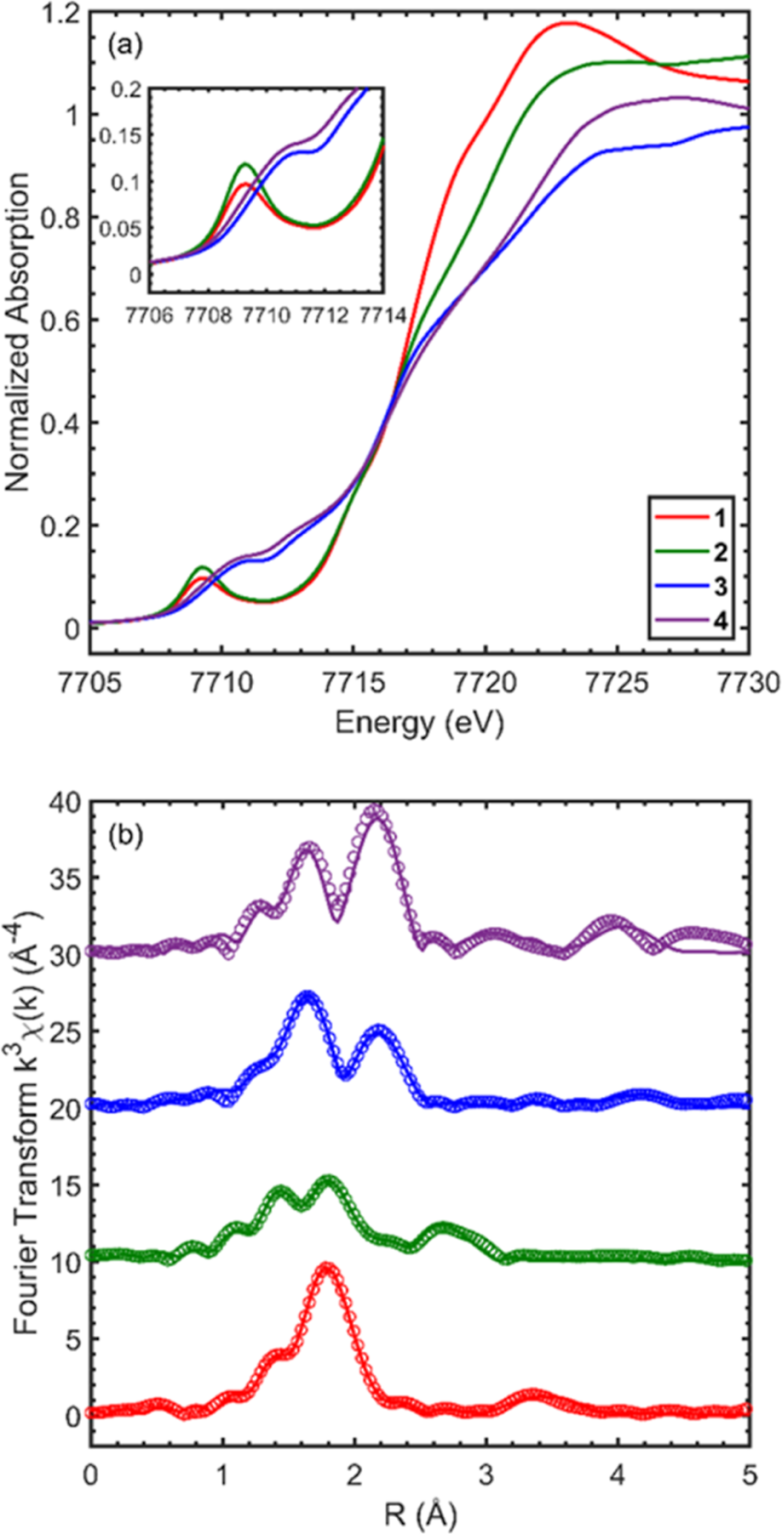
(a) Co K-edge XAS of dicobalt complexes **1**–**4**. Inset shows the expanded pre-edge region. (b) *k*^3^-weighted EXAFS Fourier transform of dicobalt complexes **1**–**4**. Measured data shown as circles, and the best-fit model is shown as solid lines.

In order to characterize the local geometry around the Co-centers of complexes **1**–**4**, as well as to probe the cobalt oxidation states, we performed Co K-edge XAS measurements (Figure 3a). The absorption spectra of **1** and **2** exhibit a pre-edge peak at 7709.3 eV that is well-separated from higher-energy rising-edge contributions. This feature arises from Co 1s → 3d transitions, which are quadrupole-allowed but dipole-forbidden. However, due to 3d-4p hybridization, this feature gains a normalized intensity of 0.1–0.15, which is consistent with tetrahedrally coordinated Co species that lack cetrosymmetry.^38,39^ Moreover, the energy of this feature is consistent with Co^2+^ species, confirming the formal Co-oxidation state in compounds **1** and **2**.^40,41^ The pre-edge transitions in **3** and **4** appear at higher energies (7709.3–7713.0 eV) and overlap with other contributions to the rising edge absorption spectra. However, the spectra of **3** and **4** exhibit no consistent red- or blue-shift along the absorption edge relative to those of **1** and **2**, and as a result, the Co-oxidation state in these compounds cannot be readily determined. An in-depth study on the detailed electronic structure and Co-ligand bonding information contained in the Co K-edge XAS spectra is reported separately.^35^

In NMR, complex **4** is very similar to **3**; however, to obtain more structural information, the Co-edge EXAFS spectra of these complexes were analyzed. The Fourier transform EXAFS of **3** (Figure 3b) shows intense scattering features between 1 and 2.5 Å (not phase-shift corrected). The data were modeled well by 1 Co–N at 2.00 ± 0.02 Å, 1 Co–P at 2.14 ± 0.03 Å, 1 Co–Si at 2.20 ± 0.03 Å, and 1 Co–Si at 2.31 ± 0.03 Å, which is consistent with the X-ray crystal structure (Figure 2).^42^ In addition, the Co–Co distance in **3** was fit to 2.51 ± 0.02 Å, which is in excellent agreement with the value of 2.5172(6) Å in the X-ray crystal structure. Finally, a low-intensity multiple scattering contribution from Co–Co–P was successfully modeled at 4.49 ± 0.04, confirming the bimetallic structure of the complex and the nearly linear P–Co–Co–P moiety.

For compound **4**, the Fourier transform EXAFS (Figure 3b) shows a notable increase in scattering intensity at long distances compared to **3**, with strong features evident up to 5 Å (not phase-shift corrected). Despite the structural similarity of **3** and **4**, the EXAFS behavior indicates slight changes in bond distances and angles between Co and the ^*t*Bu^PNNP ligand system that result in significant multiple scattering contributions from the rings. The features from 1 to 2.5 Å in the Fourier transform spectra were fitted by 1 Co–N at 2.00 ± 0.05 Å, 1 Co–P at 2.16 ± 0.04 Å, 1 Co–Si at 2.24 ± 0.04 Å, 1 Co–Si at 2.38 ± 0.04 Å, and 1 Co–Co at 2.49 ± 0.01 Å. These bond lengths for **4** are not significantly different from those found in **3**, indicating that the deprotonation process going from **3** to **4** results in only minor changes in the nearest-neighbor structure around the Co centers. A multiple scattering Co–Co–P path was modeled at 4.66 ± 0.05 Å, slightly longer than the corresponding path in **3**, suggesting an increase in the Co–Co–P angle closer to 180°. Finally, the scattering features between 2.5 and 4.5 Å were modeled by single and multiple scattering features from the ^tBu^PNNP and silane ligands. Overall, these results demonstrate a similar local geometric structure around Co in **3** and **4**, with evidence of subtle differences in the long-range single and multiple scattering EXAFS contributions.

The geometrical features of complexes **1** and **2** were also probed using EXAFS, and the geometrical parameters for both of these complexes match those determined by scXRD (Figure 1) well (see Supporting Information for detailed discussion).

### Reactivity of Complexes **3** and **4**

The reactivity of complexes **3** and **4** was tested against a variety of small molecules such as PMe_3_, benzophenone imine, H_2_, CO_2_, 1-octene, benzonitrile, 4-fluorophenyl acetylene, diethylsilane, and diphenylsilane (Table S1). What we observe for many of these substrates is that at room temperature there is no reaction, while at high temperature there is some reactivity, which then also leads to the formation of intractable mixtures. The latter is often associated by a decrease of signal intensity in the ^1^H NMR spectrum, indicative of the formation of paramagnetic decomposition products alongside various diamagnetic products observed in the NMR spectrum.

**Figure 4:**
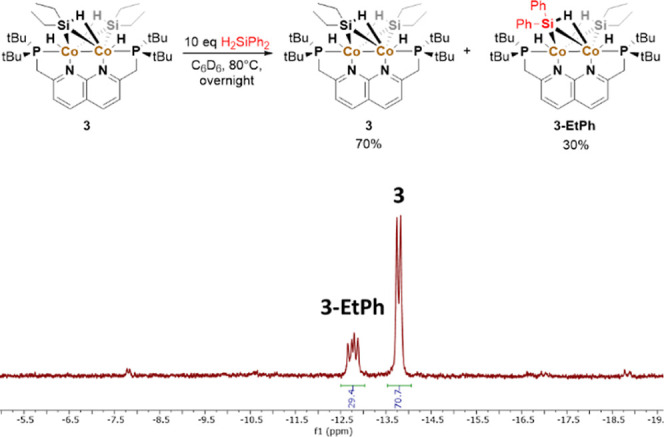
Reaction of **3** with H_2_SiPh_2_ to form the mixed silicon species **3-EtPh** (top) and the hydride region of the ^1^H NMR spectrum of the reaction (bottom).

One notable exception is diphenylsilane. When adding diphenylsilane to **3** and heating the solution to 80 °C, we observe the exchange of the silane moieties on **3** with the free silane in solution (Figure 4). This type of reactivity was also observed for other dicobalt complexes in which the silanes are bound in a similar fashion.^17^ Despite the observed silane exchange, which should proceed through a dissociative mechanism, productive exchange with other ligands suitable for low-valent cobalt such as PMe_3_ and benzophenone imine (BPI) led to either no reaction or decomposition into a complex mixture of species at higher temperatures as described. It seems that this is a general trend for complexes **3** and **4**, in which reactions take place only at higher temperatures due to the stability of the base-stabilized silylene motif. If at high temperatures a reaction takes place, that temperature may also lead to decomposition of the new product before the reaction is anywhere near completion. This indicates that the motif in **3** and **4** is more stable than that in the products that may be formed with other ligands. The somewhat remarkable stability of this silicon binding motif is also reflected in the fact that this motif, or variations on it, is encountered for various dinuclear complexes, especially those with noble metals.^43−55^ We reason that the stability of this motif is grounded in the balance between the two important resonance structures discussed above (Scheme 5). Shifting the balance from *R*1 to *R*2 provides several degrees of freedom to tailor the electronic characteristics of this motif to the demands of the metal centers, leading to stable structures.

## Conclusions

In conclusion, we have shown that the PNNP expanded pincer ligand can stabilize dicobalt cores in both high- and low-spin states. Although binding two high-spin cobalt centers in close proximity proved challenging, exemplified by the synthesis of a high-spin complex featuring a Co atom dangling outside of the dinucleating pocket, this was remedied by deprotonation of the PNNP backbone and the introduction of a bridging OtBu ligand. Reacting the high-spin dicobalt(II) complexes with secondary silanes yielded unusual diamagnetic dicobalt(II) complexes with two bridging silicon moieties (**3**) and a Co–Co bond.^35^ The silicon moieties have partial silyl and partial silylene character and are best described as base-stabilized silylenes, wherein the hydrides act as a base. We hypothesize that the base-stabilized nature of the silylene moieties diminishes the expected electrophilicity on silicon and provides unexpectedly robust dicobalt complexes. These insights can be important for understanding potential reaction or deactivation pathways for dinuclear cobalt complexes in reactions with silanes as well as for the design of dinuclear cobalt complexes in the future.

## Materials and Methods

### General Synthetic Considerations

All manipulations were performed under a N_2_ atmosphere inside a glovebox or on a Schlenk line unless mentioned otherwise. The ambient temperature in our glovebox is on average ∼27 °C. Glassware was dried at 130 °C in an oven overnight or flame-dried prior to using it. Toluene, hexane, DCM, and diethyl ether were collected from an SPS system and degassed by bubbling N_2_ through the liquid for at least 30 min. Then, they were further dried over molecular sieves. THF was distilled over Na/benzophenone ketyl (purple) and degassed by bubbling N_2_ through the liquid for at least 30 min. Benzene was degassed by bubbling N_2_ through the liquid for at least 30 min and subsequently dried over molecular sieves. Water content in the solvents was tested using Karl Fischer titration, and all but DCM were also tested by titration with a Na/benzophenone ketyl solution. Deuterated solvents were degassed using three freeze–pump–thaw cycles and dried over molecular sieves. The water content in these was tested with either Karl Fischer titration or by titration with a Na/benzophenone ketyl solution.

### Synthesis of Complex **1**

A suspension of the ^tBu^PNNP ligand (299.4 mg, 0.67 mmol, 1 equiv) in THF (20 mL) was added to a vigorously stirred suspension of CoCl_2_(THF)_1.5_ in THF (20 mL). Upon addition, the color slightly changed from deep sapphire blue to a duller, lighter blue. In addition, a blue precipitate formed. The mixture was stirred for 6 days*, after which the mixture was filtered, leaving a blue solid. The solid was washed with hexane (4 mL), after which it was dried under vacuum, yielding complex **1** as a pure blue powder in >99% yield. In some instances, additional stripping of the powder with pentane was needed to remove minor amounts of solvent from the final product. Crystals suitable for X-ray diffraction were grown from a DCM solution (0.6 mL) using vapor diffusion with diethyl ether (2.5 mL) as an antisolvent. *Consecutive syntheses showed that the reaction time can be shortened to 1 day without being detrimental to the yield.

**^1^H NMR (400 MHz, CD_2_Cl_2_, 298 K):** δ 118.5 (1H), 83.3 (1H), 79.7 (1H), 51.5 (1H), 21.2 (1H), 19.5 (1H), 5.2 (18H)*, −0.8 (9H)*, −5.9 (9H)*, −33.7 (1H) ppm. *In the region between 9 and −10 ppm, there are many broad overlapping signals, including signals that overlap with the solvent; hence, the integrals in this area are less reliable. In addition, we assume that there is one peak in that region belonging to **1** that we cannot reliably discern due to this overlap. **Effective magnetic moment in solution:** 6.1 μ_B_ measured in DCM with 2%_v/v_*o*-DFB at 298 K using the Evans method. Note that due to the low solubility of **1** (∼1.8 mg/mL), the Evans method measurement is less accurate than normally. However, considering the Co(II) oxidation state of both metals, the magnetic moment of 6.1 μ_B_ indicates an S = 3 system potentially with some antiferromagnetic coupling. This means that both cobalt(II) atoms are (mostly) independent high-spin (*S* = 3/2) centers, yielding a total *S* = 3 configuration for the dinuclear complex. **IR-ATR (cm**^**–1**^**):** 3065(w), 2960(s), 2910(s), 2869(s), 1602(s), 1505(s), 1472(m), 1372(m), 1274(w), 1180(m), 873(m), 816(m), 607(w). **Anal. Calc. for C**_**26**_**H**_**44**_**Cl**_**4**_**Co**_**2**_**N**_**2**_**P**_**2**_**:** C, 44.22; H, 6.28; N, 3.96. Found C, 44.63; H, 6.12; N, 3.56.

### Synthesis of Complex **2**

A solution of KOtBu (252.8 mg, 2.25 mmol, 2 equiv) in THF (7 mL) was added dropwise to a vigorously stirred suspension of ^tBu^PNNP (503.3 mg, 1.13 mmol, 1 equiv) in THF (25 mL), resulting in an immediate color change to red. After 20 min, a dark red solution was added dropwise to a suspension of CoCl_2_(THF)_1.5_ in THF (25 mL). During the addition, the color of the suspension changed from blue to green and finally to brown. The solution was stirred for 2 h after which the solvent was evaporated, yielding a dark solid. The solid was suspended in pentane (50 mL), and the suspension was filtered, leaving a brown residue. The residue was washed with pentane (5 mL) and extracted with toluene*, leaving a minor blue residue on the filter. The brown filtrate was dried and stripped with hexane, yielding 0.5901 g (74%) of **2** as a brown air- and moisture-sensitive solid. In some batches, some remaining solvent was found in **2**, and in these cases, this can be removed by dissolving **2** in benzene and freeze-drying the sample. Crystals of **2** suitable for X-ray diffraction were obtained by slow vapor diffusion of hexane into a toluene solution of the complex. * Extraction with diethyl ether is not suitable since it led to some of the blue starting material/byproduct to dissolve and end up in the filtrate.

**^1^H NMR (400 MHz, C_6_D_6_, 298 K):** δ 197.51 (1H), 74.9 (1H), 43.8 (1H), 42.3 (1H), 41.9 (1H), 26.9 (9H), 18.3 (1H), 13.6 (9H), 8.9 (9H), 7.3 (9H), 6.8 (1H), 5.5 (9H) ppm. **Effective magnetic moment in solution:** 6.6 μ_B_ measured in THF with 2%_w/w_*o*-DFB at 298 K using the Evans method. This indicates a S = 3 system consisting of two independent high-spin (S = 3/2) cobalt(II) centers. **IR-ATR (cm**^**–1**^**):** 2960(s), 2900(s), 2865(s), 1629(w), 1553(m), 1505(s), 1413(s), 1323(m), 1261(w), 1181(w), 1138(w), 1022(w), 805(m). **Anal. Calc. for C**_**30**_**H**_**52**_**Cl**_**2**_**Co**_**2**_**N**_**2**_**OP**_**2**_**:**C, 50.93; H, 7.41; N, 3.96. Found C, 50.20; H, 7.49; N, 3.49. The reactive nature of this compound precluded obtaining a satisfactory elemental analysis. The values that are found are consistent with the incorporation of ∼1 molecule of H_2_O toward which this compound is very sensitive due to its partially dearomatized backbone and hence the basic methylene linker.

### Synthesis of Complex **3**

Diethylsilane (1.4 mL, 10.8 mmol, 51 equiv) was added to a suspension of complex **1** (150.2 mg, 0.213 mmol, 1 equiv) in toluene (27 mL) in a Schlenk bomb with J-Young valve*. The sample was heated to 110 °C for 30 min with stirring, during which the blue suspension turned into a black solution (Figure S7)***. The black solution did not contain any visible blue precipitate anymore, and the solvent was evaporated. The resulting black solid was washed with hexane (3 × 1.6 mL) and extracted with toluene (∼7 mL). Removing all volatiles under a dynamic vacuum yielded 119.2 mg (76%) of **3** as a crystalline powder. Crystals suitable for X-ray diffraction were grown through vapor diffusion from a toluene solution of **3** with hexane as the antisolvent.

*The use of a vessel with a J-young valve is needed since the reaction takes place far above the boiling point of diethylsilane.
**If the reaction proceeds (see **), leaving it at 110 °C for longer than 30 min leads to lower yields due to the formation of a side product.
***The reaction is very sensitive to temperature and to how deep it is in the oil bath. We had cases in which putting the liquid 1–3 mm deeper into the oil bath made the difference between the reaction taking place or not at all. If the solution is not discolored after 30 min, the Schlenk bomb should be put deeper into the oil bath such that the liquid in the bomb is at least a few mm underneath the level of the oil in the bath. Thirty minutes after adjusting the Schlenk bomb, the mixture should be fully discolored, and the rest of the protocol can be followed.

**^1^H NMR (400 MHz, C_6_D_6_, 298 K):** δ 7.00 (d, ^3^*J*_H,H_ = 8.0 Hz, 2H), 6.37 (d, ^3^*J*_H,H_ = 8.0 Hz, 2H), 2.75 (d, ^2^*J*_H,P_ = 7.5 Hz, 4H), 1.47 (m, 10H), 1.30 (d, ^3^*J*_H,P_ = 11.9 Hz, 36H), 1.10 (t, ^3^*J*_H,H_ = 7.7 Hz, 6H), 0.80 (q, ^3^*J*_H,H_ = 7.7 Hz, 4H), −13.73 (d, ^2^*J*_H,P_ = 35.5 Hz, 4H) ppm. ^**31**^**P NMR (162 MHz, C**_**6**_**D**_**6**_, **298 K):** δ 117.9 (br s) ppm ^**13**^**C NMR (101 MHz, C**_**6**_**D**_**6**_, **298 K):** δ 163.1 (d, ^2^*J*_P,C_ = 9.6 Hz), 162.7 (s), 126.9 (s), 125.3 (s), 119.7 (d, ^3^*J*_P,C_ = 8.6 Hz), 39.8 (d, ^1^*J*_P,C_ = 5.4 Hz), 34.8 (d, ^1^*J*_P,C_ = 8.5 Hz), 29.9 (s), 15.8 (s), 14.6 (s), 12.5 (s), 12.5 (s), 12.1 (s) ppm. ^**29**^**Si NMR determined with HMBC (79 MHz, C**_**6**_**D**_**6**_, **298 K):** δ 136.1 ppm. **IR-ATR (cm**^**–1**^**):** 2942(s), 2892(s), 2860(s), 1916(bw), 1622(w), 1506(m), 1456(m), 1365(m), 1307(m), 1213(w), 1180(w), 1153(w), 1019(w), 1020(w), 839(w), 811(w), 693(w), 602(w). **Anal. Calc. for:** C, 55.12; H, 9.25; N, 3.78. Found C, 52.50; H, 8.95; N, 3.57. The reactive nature of this compound precluded obtaining satisfactory elemental analysis. The values that are found are consistent with the incorporation of one molecule of O_2_ toward which this complex is very sensitive.

### Synthesis of Complex **4**

A solution of KOtBu (7.8 mg, 69.5 mmol, 1 equiv) and 18-crown-6 (18.4 mg, 69.6 mmol, 1 equiv) in THF (5.5 mL) was added dropwise to a solution of complex **3** (51.2 mg, 69.1 μmol) in THF (5.5 mL). During the addition, the color of the mixture changed from red brown to dark green. The mixture was stirred for 75 min after which the solvent was evaporated to yield a dark solid. The solid was suspended in hexane (6 mL) and filtered. The green residue was washed with hexane (3 × 2 mL) and subsequently extracted with THF (∼4 mL). The THF extract was dried under a dynamic vacuum, yielding **4** (62.8 mg, 77%) as a dark green powder.

**^1^H NMR (400 MHz, C_6_D_6_, 298 K):** δ 6.27 (d, ^3^*J*_H,H_ = 7.2 Hz, 1H), 6.15 (d, ^3^*J*_H,H_ = 8.7 Hz, 1H), 5.93 (m, 2H), 4.25 (d, ^2^*J*_H,P_ = 1.6 Hz, 1H), 4.25 (d, ^2^*J*_H,P_ = 1.6 Hz, 1H), 3.61 (br s, 24H), 1.35 (d, ^3^*J*_H,P_ = 10.9 Hz, 18H), 1.34 (d, ^3^*J*_H,P_ = 11.1 Hz, 18H), 0.80 (m, 10H), 0.69 (m, 10H) ppm. ^**31**^**P NMR (162 MHz, C**_**6**_**D**_**6**_, **298 K):** δ 109.7, 108.3 ppm. ^**13**^**C NMR (101 MHz, C**_**6**_**D**_**6**_, **298 K):** δ 167.6 (d, ^2^*J*_C,P_ = 24.6 Hz), 165.1 (s), 162.6 (d, ^2^*J*_C,P_ = 11.4 Hz), 128.0 (s), 125.1 (s), 119.3 (d, ^3^*J*_C,P_ = 15.6 Hz), 115.8 (s), 106.0 (d, ^3^*J*_C,P_ = 8.9 Hz), 84.5 (d, ^1^*J*_C,P_ = 27.4 Hz), 71.3 (s), 39.9 (d, ^1^*J*_C,P_ = 4.7 Hz), 35.3 (d, ^1^*J*_C,P_ = 12.6 Hz), 34.9 (d, ^1^*J*_C,P_ = 6.2 Hz), 32.2 (d, ^2^*J*_C,P_ = 6.3 Hz), 31.0 (d, ^2^*J*_C,P_ = 6.1 Hz), 16.3 (br s), 15.2 (s), 12.3 (s) 12.2 (s) ppm. ^**29**^**Si NMR determined with HMBC (79 MHz, C**_**6**_**D**_**6**_, **298 K):** δ 127 ppm. **IR-ATR (cm**^**–1**^**):** 2888(s), 2855(s), 1886(bw), 1611(w), 1529(w), 1494(m), 1409(m), 1351(w), 1109(s), 963(w). **Anal. Calc. for:** C, 52.96; H, 8.79; N, 2.69. Found C, 46.53; H, 8.05; N, 2.25. The reactive nature of this compound precluded obtaining satisfactory elemental analysis.

### Computational Methods

Calculations were performed using Gaussian 16 rev. C01 software.^56^ The Becke 3-parameter Lee–Yang–Parr (B3LYP) functional was used.^57,58^ The redefinition of Ahlrichs triple-ζ split valence basis set (def2-TZVP) was used on all atoms, except for in the relaxed potential energy surface scan.^59^ For all calculations, DFT-D3 dispersion correction with Becke-Johnson damping was used.^60,61^ The Fukui functions were calculated by subtracting the electron density of **3** from the electron density of the radical anion of **3** using the MultiWFN program.^62^ Visualization of the Fukui function was done in Jmol.^63^ The starting geometry for the optimization of **3** was obtained from the coordinates of the solid-state structure. The starting geometry for **4** was obtained by removing a hydrogen atom from the structure of **3** and modifying the charge accordingly. The relaxed potential energy surface scan was performed with the redefinition of the Ahlrichs split valence basis set (def2-SVP) instead of the def2-TZVP basis set used for all other optimizations. Quantum theory of atoms in molecules calculations were performed using the MultiWFN program, and natural bonding orbital (NBO) calculations were performed with the NBO 6.0 software.^62,64^

### EXAFS Measurements

Co K-edge X-ray absorption spectra were measured at the Stanford synchrotron radiation lightsource on the unfocused 20-pole 2T wiggler side-station beamline 7–3 under standard ring conditions of 3 GeV and ∼500 mA. A Si(220) double crystal monochromator was used for energy selection with a crystal orientation φ = 0°. Bulk solid samples were stored and handled in a dry N_2_ glovebox. Samples were prepared by grinding ∼5 mg of sample (mass calculated to yield ∼1 absorption length above the Co K-edge) with ∼25 mg of dry boron nitride in an agate mortar and pestle to form a uniformly colored, fine powder. The powder was pressed into a 7 mm diameter cylindrical pellet and held between 64 μm of Kapton tape. During data collection, the sample was held in a Cryo Industries closed-cycle liquid He cryostat and maintained at ∼10 K throughout the measurement. Spectra were measured to *k* = 14.1 Å^–1^ in the transmission mode using N_2_-filled ionization chambers. The X-ray beam size was 1.2 × 4 mm (height) for all measurements. A Co foil was measured simultaneously for energy calibration, and the first inflection point in the Co foil spectrum was fixed at 7709 eV.

Data processing was performed in the Athena program of the Demeter package.^65^ The data presented here were obtained by aligning and merging two scans on different sample spots to ensure minimal beam damage. The postedge EXAFS background was modeled in the Pyspline program using a three-region spline of orders 2, 3, and 3.^66^ EXAFS was modeled by using the Artemis program of the Demeter package. Theoretical EXAFS signals χ(*k*) were calculated by using FEFF6. Absorber-backscatter paths were generated from the atomic coordinates by using DFT-optimized structures. The EXAFS model was optimized in *k*-space using *k*^1^, *k*^2^, and *k*^3^ weightings, with the model obeying the Nyquist criterion.^67^ EXAFS fits were performed between a *k*-range of 2–14 Å^–1^ and varying *R*-ranges. Structural parameters that varied during the fitting process were the bond distance (*R*) and bond variance (σ^2^). The nonstructural parameter Δ*E*_0_ (*E*_0_ is the energy at which *k* equals 0) was also fit. Coordination numbers were systematically varied over the course of the fitting process to assess different models but were fixed during a given fit. The value of *S*_0_^2^ was set to 0.9 for all fits.

All other information pertaining to the experimental and computational materials and methods used in this study is provided in the Supporting Information.

## Supporting Information Available

NMR datafiles and DFT output files supporting this work can be obtained free of charge from doi.org/10.24416/UU01-7PL9KM. Experimental and computational details for this manuscript, synthetic procedures and spectroscopic data of complexes **1**, **2**, **3,** and **4**, and the crystal structure determinations of compounds **1**, **2,** and **3**. (PDF)

## Notes

An earlier version of this manuscript was upload to ChemRxiv as a preprint.^68^
