# Supplementary material for: Chloride, Alkoxide, or Silicon: The Bridging Ligand Dictates the Spin State in Dicobalt Expanded Pincer Complexes
Source: Organometallics. 2024 Nov 28;44(1):94–104. doi: 10.1021/acs.organomet.4c00374 (PMC11734127; doi:10.1021/acs.organomet.4c00374)
Supplement: Supplementary file 1 — om4c00374_si_001.pdf [file om4c00374_si_001.pdf]

Supporting information
for:
Chloride, Alkoxide or Silicon: The Bridging Ligand Dictates the Spin-state in Dicobalt Expanded Pincer Complexes

Roel L. M. Bienenmann,[a] Arun S. Asundi,[b] Martin Lutz,[c] Ritimukta Sarangi,[b] Daniël L. J. Broere[a]*

[a]Organic Chemistry and Catalysis, Institute for Sustainable and Circular Chemistry, Faculty of Science, Utrecht University, Universiteitsweg 99, 3584 CG, Utrecht, The Netherlands. [b]Stanford Synchrotron Radiation Lightsource, SLAC National Accelerator Laboratory, Stanford University, 94025, Menlo Park, California, USA. [c]Structural Biochemistry, Bijvoet Centre for Biomolecular Research, Faculty of Science, Utrecht University, Universiteitsweg 99, 3584 CG, Utrecht, The Netherlands. Email: d.l.j.broere@uu.nl.

Contents

Experimental methods 3
General considerations 3
Synthesis of $^{tBu}PNNPCo_2Cl_4$ (**1**) 3
Synthesis of $^{tBu}PNNP^*Co_2OtBuCl_2$ (**2**) 6
Synthesis of $^{tBu}PNNPCo_2(SiEt_2)_2H_4$ (**3**) 9
VT NMR of complex **3** 15
Synthesis of $[^{tBu}PNNP^*Co_2(SiEt_2)_2H_4]$K18-crown-6 (**4**) 16
Protonation of **4** with $HBArF_{24}$ 21
Tested reactions with **3** and **4** 23
Reaction of **3** with PMe_3 23
Reaction of **3** with benzophenone imine 23
Reaction of **3** with diphenylsilane 23
Reaction of **3** with 4-fluoroacetylene 23
Reaction of **3** with diethylsilane and 4-fluoroanisole 23
Reaction of **3** with H_2 23
Reaction of **4** with 4-fluoroacetylene 24
Reaction of **4** with 1-octene and CO_2 24
Reaction of **4** with benzonitrile and diethylsilane 24
Reaction of **4** with 4-chlorobenzaldehyde 24
Reaction of **4** with Butylacrylate 24
Computational methods 25
General considerations 25
Overlay calculated and measured structure of **3** 25
Fukui function of **3** 25

Hydride exchange mechanism of complex **3** 26
Optimized structure of **4** 27
QTAIM analysis of complex **3** 27
NBO analysis of complex **3** 28
X-ray crystal structure determinations 29
X-ray crystal structure determination of **1** 29
X-ray crystal structure determination of **2** 29
X-ray crystal structure determination of **3** 30
EXAFS measurements 30
General considerations 30
EXAFS fit results for complexes **1-4** 31
EXAFS analysis of complexes **1** and **2** 33
References 33

Experimental methods

General considerations

All manipulations were performed under N_2 atmosphere inside a glovebox or on a Schlenk line unless mentioned otherwise. The ambient temperature in our glovebox is on average ~27°C. Glassware was dried at 130°C in an oven overnight or flame dried prior using it. Toluene, hexane, DCM and diethyl ether were collected from an SPS system and degassed by bubbling N_2 through the liquid for at least 30 min. Then they were further dried over molecular sieves. THF was distilled over Na/benzophenone ketyl (purple) and degassed by bubbling N_2 through the liquid for at least 30 min. Benzene was degassed by bubbling N_2 through the liquid for at least 30 min and subsequently dried over molecular sieves. Water content in the solvents was tested using Karl-Fisher titration and all but DCM were also tested by titration with a Na/benzophenone ketyl solution. Deuterated solvents were degassed using three freeze pump thaw cycles and dried over molecular sieves. The water content in these were tested with either Karl-Fisher titration or by titration with a Na/benzophenone ketyl solution. The PNNP ligand[1], HBArF24•2Et2O (Brookhart's acid)[2] and $CoCl_2(THF)_{1.5}$[3] were synthesized according to literature procedures. Other chemicals were obtained from commercial sources. Liquids were degassed using 3 freeze-pump-thaw cycles before use and solids were degassed under vacuum before use. NMR spectra were recorded on a Varian MRF 400 equipped with a OneNMR probe and Optima Tune system, a Varian VNMR-S-400 equipped with a PFG probe or a 400 MHz Jeol EZCL G system with an HFX probe and 90 G/cm gradient amplifier. All resonances in ^{1}H NMR were referenced to residual solvent peaks.[4] IR-data was recorded on a PerkinElmer SpectrumTwo Infrared Spectrophotometer equipped with an ATR-probe. Elemental analysis was performed by MEDAC ltd. in the United Kingdom.

Synthesis of $^{tBu}PNNPCo_2Cl_4$ (1)

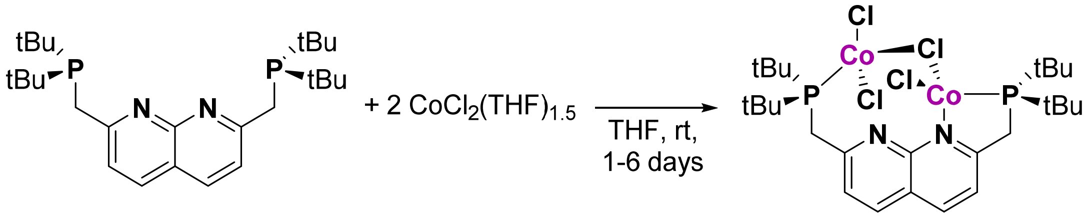

1

A suspension of the ^{tBu}PNNP ligand (299.4 mg, 0.67 mmol, 1 eq) in THF (20 mL) was added to a vigorously stirred suspension of $CoCl_2(THF)_{1.5}$ in THF (20 mL). Upon addition, the color slightly changed from a deep sapphire blue to a more dull lighter blue. In addition, a blue precipitated formed. The mixture was stirred for 6 days* after which the mixture was filtered leaving a blue solid. The solid was washed with hexane (4 mL) after which it was dried under vacuum yielding complex **1** as a pure blue powder in >99% yield. In some instances, additional stripping of the powder with pentane was needed to remove minor amounts of solvent from the final product. Crystals suitable for X-ray diffraction were grown from a DCM solution (0.6 mL) using vapor diffusion with diethyl ether (2.5 mL) as antisolvent.

*Consecutive syntheses showed that the reaction time can be shortened to 1 day without being detrimental to the yield.

^{1}H NMR (400 MHz, DCM-d2, 298 K): δ 118.5 (1H), 83.3 (1H), 79.7 (1H), 51.5 (1H), 21.2 (1H), 19.5 (1H), 5.2 (18 H)*, -0.8 (9H)*, -5.9 (9H)*, -33.7 (1H) ppm.

*in the region between 9 and -10 ppm, there are many broad overlapping signals, including signals that overlap with the solvent, hence the integrals in this area are less reliable. In addition, we assume that there is one peak in that region belonging to **1** that we cannot reliably decern due to this overlap.

Effective magnetic moment in solution: 6.1 μ_B measured in DCM with $2\%_{v/v}$ o-DFB at 298 K using Evans method. Note that due to the low solubility of **1** (~1.8 mg/mL), the Evans method measurement is less accurate than normally. However, considering the Co(II) oxidation state of both metals, the magnetic moment of 6.1 μ_B indicates an S = 3 system potentially with some antiferromagnetic coupling. This means that both cobalt(II)

atoms are (mostly) independent high-spin (S = 3/2) centers, yielding a total S = 3 configuration for the dinuclear complex.

IR-ATR (cm^{-1}): 3065(w), 2960(s), 2910(s), 2869(s), 1602(s), 1505(s), 1472(m), 1372(m), 1274(w), 1180(m), 873(m), 816(m), 607(w).

Anal. Calc. for $C_{26}H_{44}Cl_4Co_2N_2P_2$: C, 44.22; H, 6.28; N, 3.96. Found C, 44.63; H, 6.12; N, 3.56.

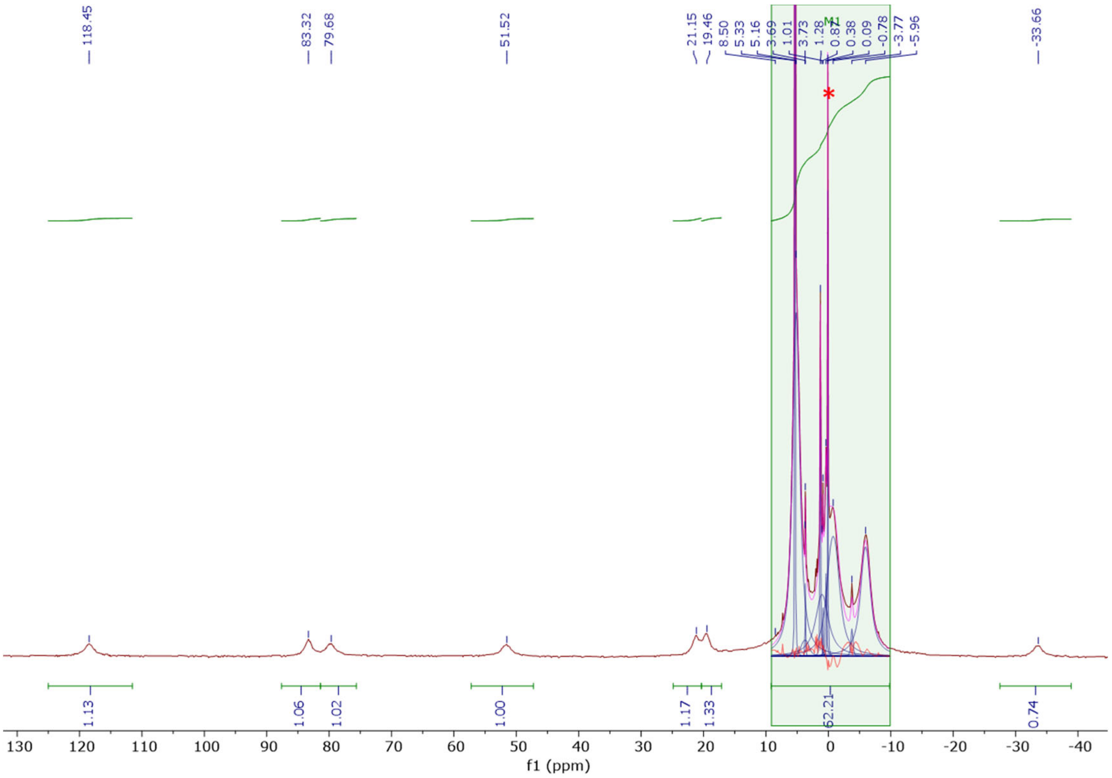

*Figure S1: ^{1}H NMR(298K, CD_2Cl_2) of complex **1**. * denotes grease.*

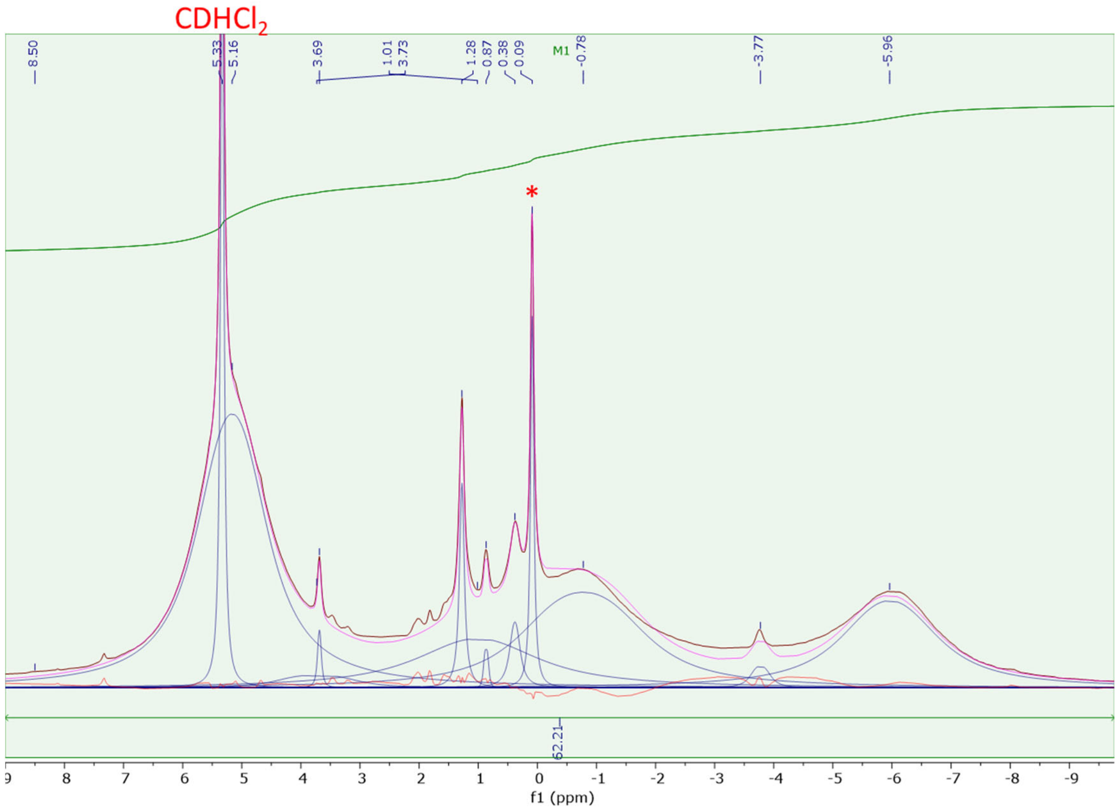

Figure S2: The peak fitted area from the [1]*H NMR(298K,* CD_2Cl_2*) of complex* ***1*** *containing several resonances of* ***1*** *(at 5.16, -0.78 and -5.96 ppm) as well as the* $CDHCl_2$ *solvent residual and grease (*). The total integral of this region relative to the peak at 51.5 ppm is 62.21, meaning that the normalized integrals of the peaks at 5.16, -0.78 and -5.96 ppm are 23, 13 and 10 respectively. It should be noted that these values do have a large uncertainty though due to the fitting procedure and the large extend of overlap as well as the width of the peaks.*

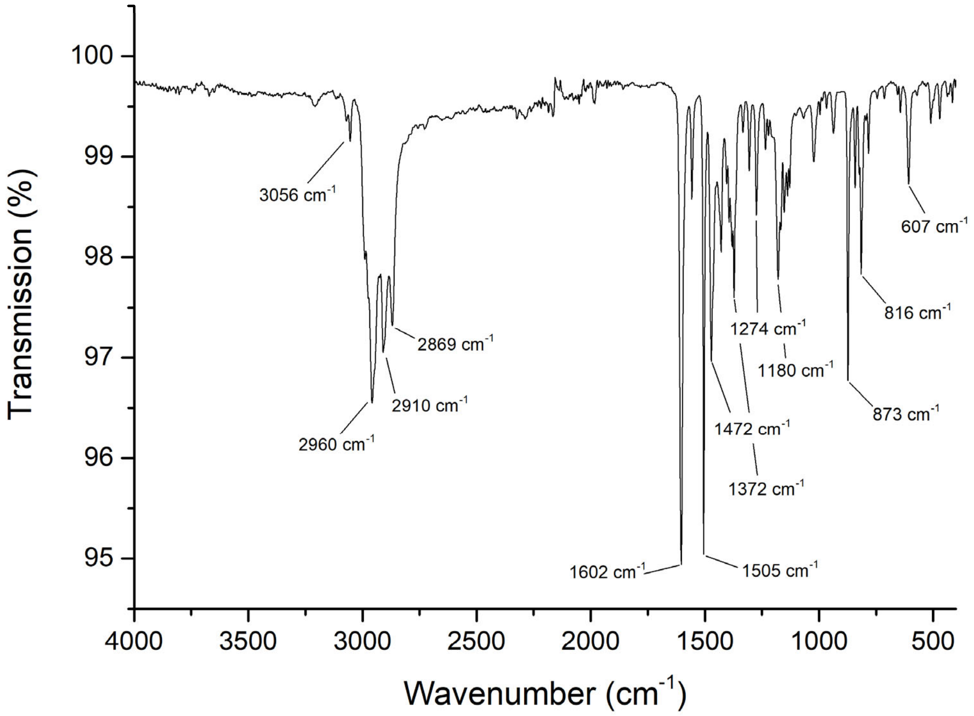

*Figure S3: FTIR (ATR) spectrum of complex **1**.*

Synthesis of ^{tBu}PNNP*Co_2OtBuCl_2 (2)

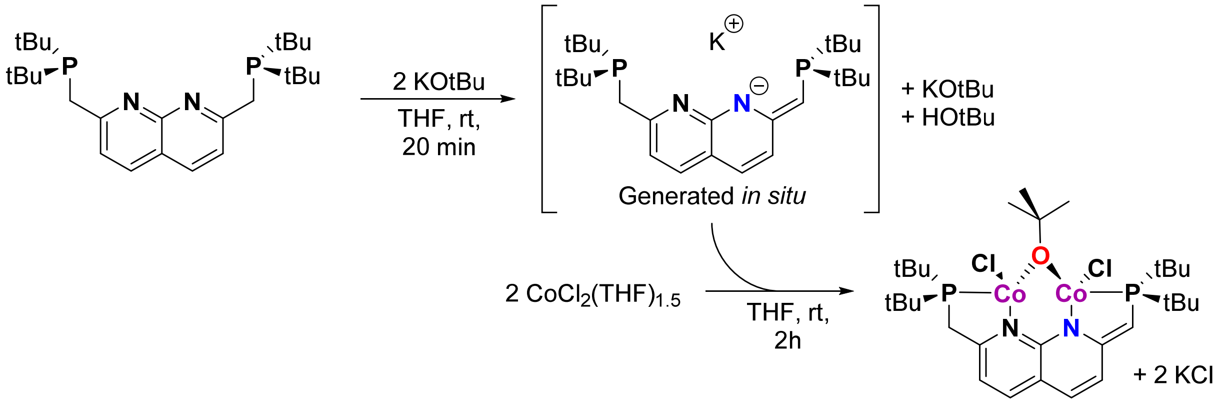

A solution of KOtBu (252.8 mg, 2.25 mmol, 2 eq) in THF (7 mL) was added dropwise to a vigorously stirred suspension of ^{tBu}PNNP (503.3 mg, 1.13 mmol, 1 eq) in THF (25 mL), resulting in an immediate color change to red. After 20 min, the dark red solution was added dropwise to a suspension of $CoCl_2(THF)_{1.5}$ in THF (25 mL). During the addition, the color of the suspension changed from blue to green and finally to brown. The solution was stirred for 2 h after which the solvent was evaporated yielding a dark solid. The solid was suspended in pentane (50 mL) and the suspension was filtered leaving a brown residue. The residue was washed with pentane (5 mL) and extracted with toluene* leaving a minor blue residue on the filter. The brown filtrate was dried and stripped with hexane yielding 0.5901g (74%) of **2** as a brown air and moisture sensitive solid. In some batches, some remaining solvent was found in **2**, in these cases, this can be removed by dissolving **2** in benzene and freeze-drying the sample. Crystals of **2** suitable for X-ray diffraction were obtained by slow vapor diffusion of hexane into a toluene solution of the complex.

* Extraction with diethyl ether is not suitable since it led to some of the blue starting material/by product to dissolve and end up in the filtrate.

^{1}H NMR (400 MHz, C_6D_6, 298 K): δ 197.51 (1H), 74.9 (1H), 43.8 (1H), 42.3 (1H), 41.9 (1H), 26.9 (9H), 18.3 (1H), 13.6 (9H), 8.9 (9H), 7.3 (9H), 6.8 (1H), 5.5 (9H) ppm.

Effective magnetic moment in solution: 6.6 μ_B measured in THF with $2\%_{w/w}$ o-DFB at 298 K using Evans method. This indicates a S = 3 system consisting of two independent high spin (S =3/2) cobalt(II) centers.

IR-ATR (cm^{-1}): 2960(s), 2900(s), 2865(s), 1629(w), 1553(m), 1505(s), 1413(s), 1323(m), 1261(w), 1181(w), 1138(w), 1022(w), 805(m).

Anal. Calc. for $C_{30}H_{52}Cl_2Co_2N_2OP_2$: C, 50.93; H, 7.41; N, 3.96. Found C, 50.20; H, 7.49; N, 3.49. The reactive nature of this compound precluded obtaining satisfactory elemental analysis. The values that are found are consistent with the incorporation of ~1 molecule of H_2O towards which this compound is very sensitive due to its partially dearomatized backbone and hence basic methylene linker.

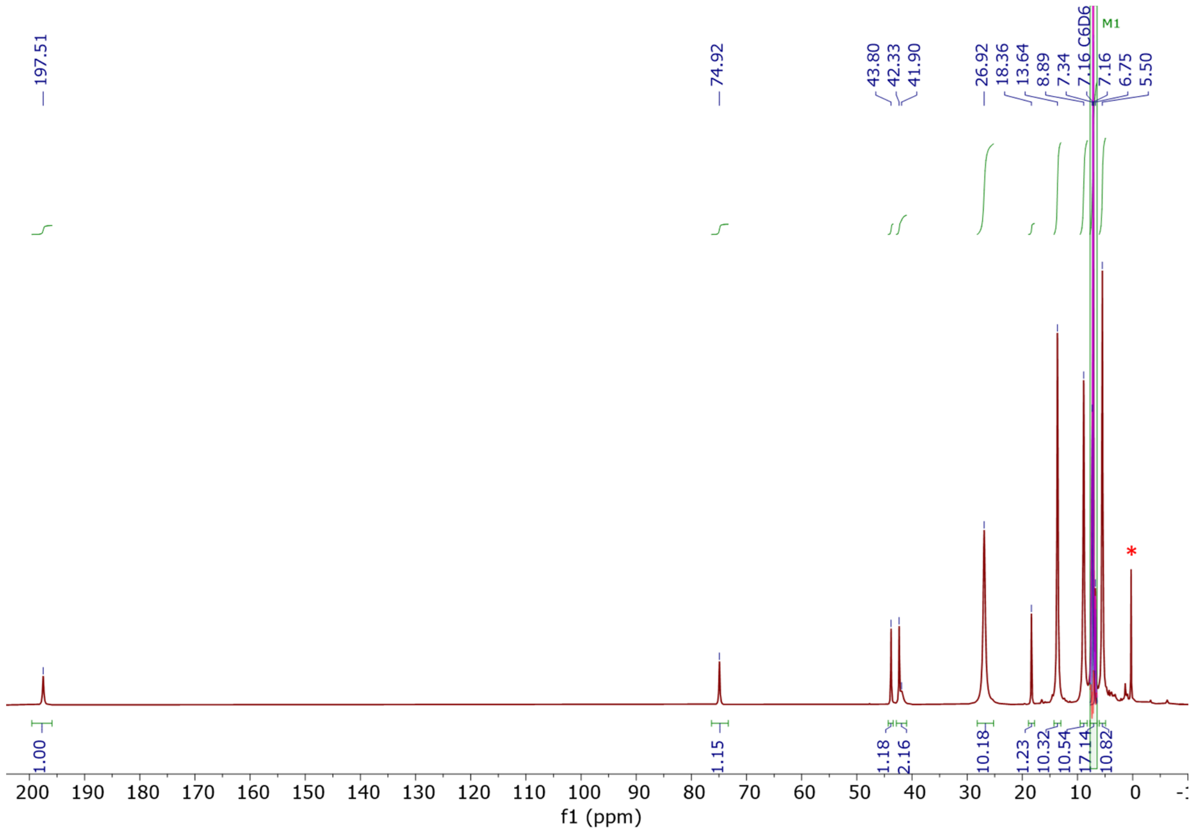

*Figure S4: ^{1}H NMR(298K, C_6D_6) of complex **2**. * denotes grease.*

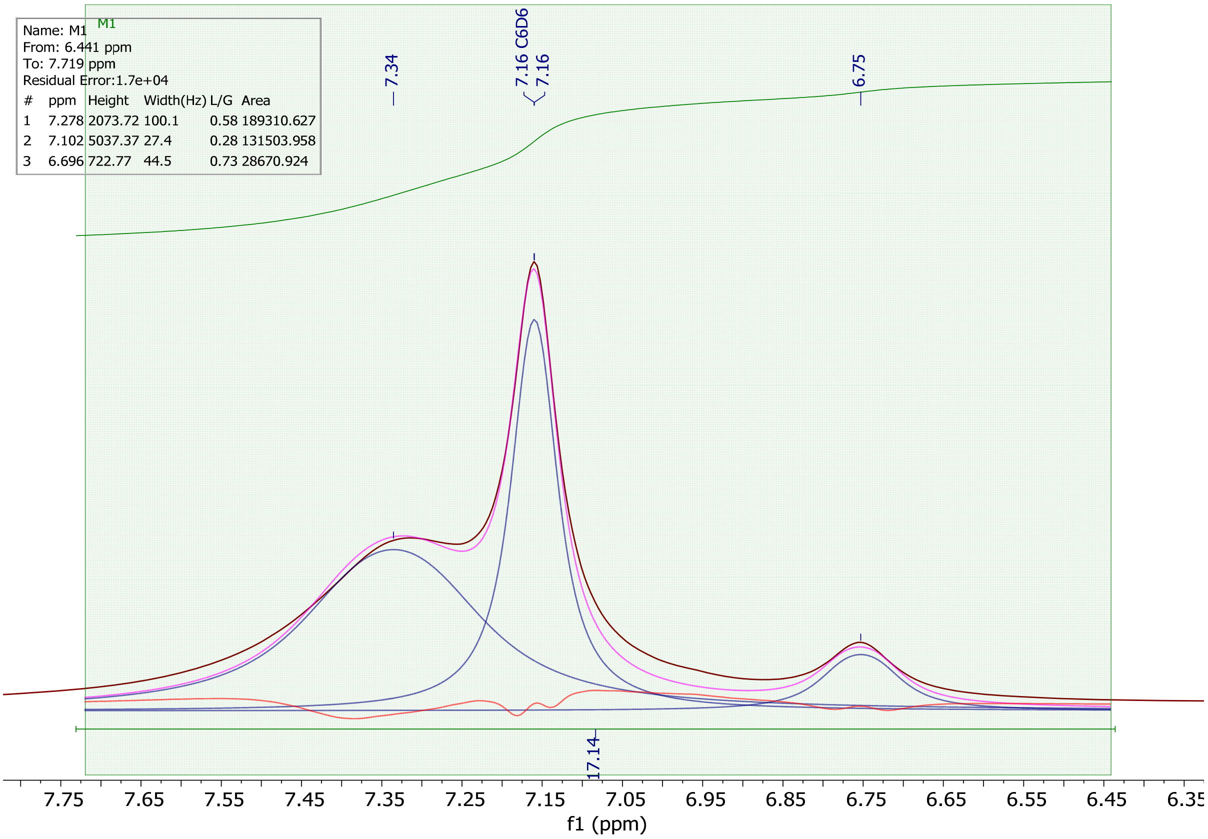

*Figure S5: The peak fitted area from the ^{1}H NMR(298K, C_6D_6) of complex **2** containing two resonances of **2** (at 7.34 and 6.75 ppm) as well as the C6D5H solvent residual (7.16 ppm). The total integral of this region relative to the peak at 197.51ppm is 17.14, meaning that the normalized integrals of the peaks at 7.34 and 6.75 ppm are 9.3 and 1.4 respectively.*

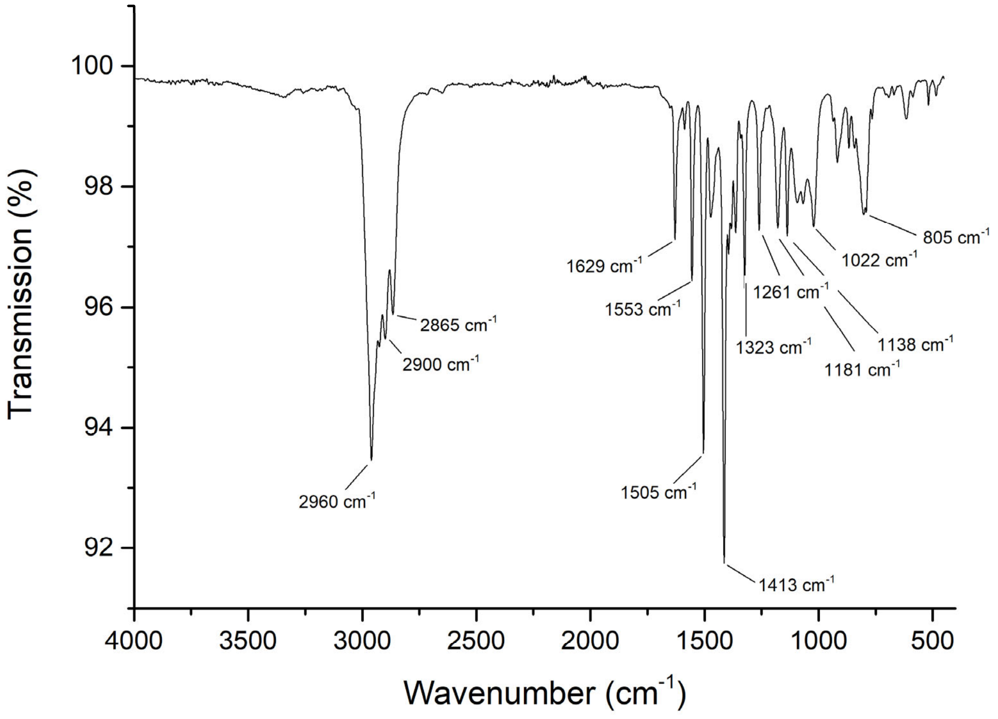

*Figure S6: FTIR (ATR) spectrum of complex **2**.*

Synthesis of $^{tBu}PNNPCo_2(SiEt_2)_2H_4$ (3)

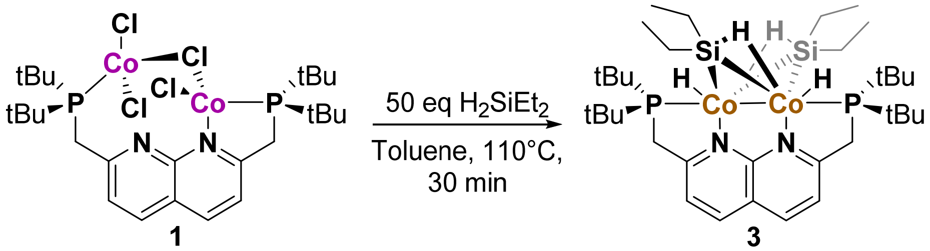

Diethylsilane (1.4 mL, 10.8 mmol, 51 eq) was added to a suspension of complex **1** (150.2 mg, 0.213 mmol, 1 eq) in toluene (27 mL) in a Schlenk bomb with J-Young valve*. The sample was heated to 110°C for 30 minutes** while stirring, during which the blue suspension turned into a black solution (Figure S7)***. The black solution did not contain any visible blue precipitate anymore and the solvent was evaporated. The resulting black solid was washed with hexane (3x 1.6 mL) and extracted with toluene (~7 mL). Removing all volatiles under a dynamic vacuum yielded 119.2 mg (76%) of **3** as a crystalline powder. Crystals suitable for X-ray diffraction were grown through vapor diffusion from a toluene solution of **3** with hexane as anti-solvent.

*The use of a vessel with a J-young valve is needed since the reaction takes place far above the boiling point of diethylsilane.

If the reaction proceeds (see *), leaving it at 110°C for longer than 30 minutes leads to lower yields due to the formation of a side product.

***The reaction is very sensitive to temperature and to how deep it is in the oil bath. We had cases in which putting the liquid 1-3 mm deeper into the oil bath made the difference between the reaction taking place or not at all. If the solution is not discolored after 30 min, the Schlenk bomb should be put deeper into the oil bath such

that the liquid in the bomb is at least a few mm underneath the level of the oil in the bath. 30 minutes after adjusting the Schlenk bomb, the mixture should be fully discolored and the rest of the protocol can be followed.

^{1}H NMR (400 MHz, C_6D_6, 298 K): δ 7.00 (d, $^{3}J_{H,H}$ = 8.0 Hz, 2H), 6.37 (d, $^{3}J_{H,H}$ = 8.0 Hz, 2H), 2.75 (d, $^{2}J_{H,P}$ = 7.5 Hz, 4H), 1.47 (m, 10H), 1.30 (d, $^{3}J_{H,P}$ = 11.9 Hz, 36H), 1.10 (t, $^{3}J_{H,H}$ = 7.7 Hz, 6H), 0.80 (q, $^{3}J_{H,H}$ = 7.7 Hz, 4H), -13.73 (d, $^{2}J_{H,P}$ = 35.5 Hz, 4H) ppm.

^{31}P NMR (162 MHz, C_6D_6, 298 K): δ 117.9 (bs) ppm

^{13}C NMR (101 MHz, C_6D_6, 298 K): δ 163.1 (d, $^{2}J_{P,C}$ = 9.6 Hz), 162.7 (s), 126.9 (s), 125.3 (s), 119.7 (d, $^{3}J_{P,C}$ = 8.6 Hz), 39.8 (d, $^{1}J_{P,C}$ = 5.4 Hz), 34.8 (d, $^{1}J_{P,C}$ = 8.5 Hz), 29.9 (s), 15.8 (s), 14.6 (s), 12.5 (s), 12.5 (s), 12.1 (s) ppm.

^{29}Si NMR determined with HMBC (79 MHz, C_6D_6, 298 K): δ 136.1 ppm

IR-ATR (cm^{-1}): 2942(s), 2892(s), 2860(s), 1916(bw), 1622(w), 1506(m), 1456(m), 1365(m), 1307(m), 1213(w), 1180(w), 1153(w), 1019(w), 1020(w), 839(w), 811(w), 693(w), 602(w).

Anal. Calc. for: C, 55.12; H, 9.25; N, 3.78. Found C, 52.50; H, 8.95; N, 3.57. The reactive nature of this compound precluded obtaining satisfactory elemental analysis. The values that are found are consistent with the incorporation of one molecule of O_2 towards which this complex is very sensitive.

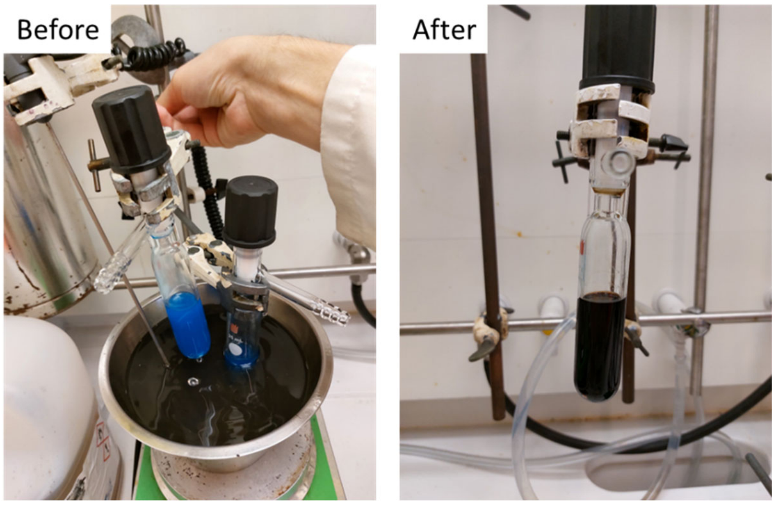

*Figure S7: The reaction mixture for the synthesis of **3** before and after heating at 110°C for 30 minutes.*

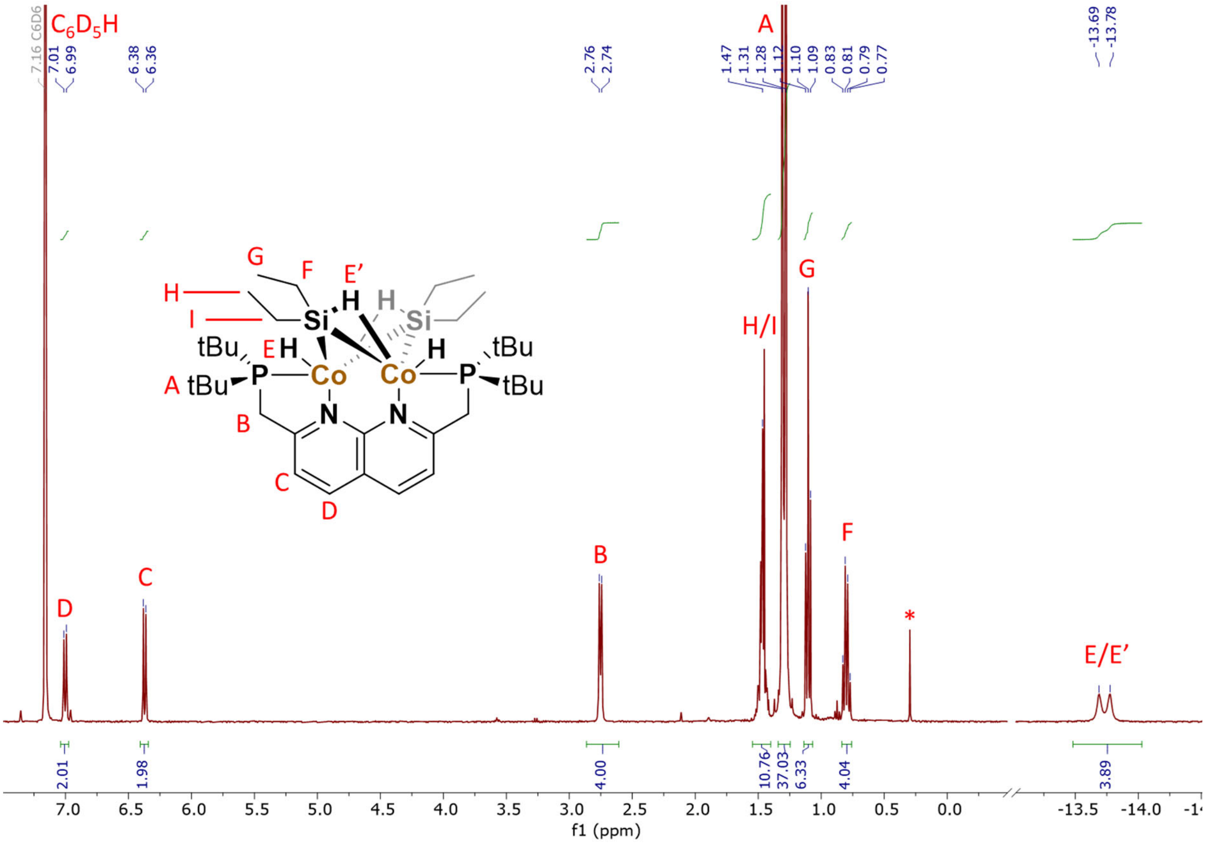

*Figure S8: ¹H NMR(298K, C_6D_6) of complex **3**.*

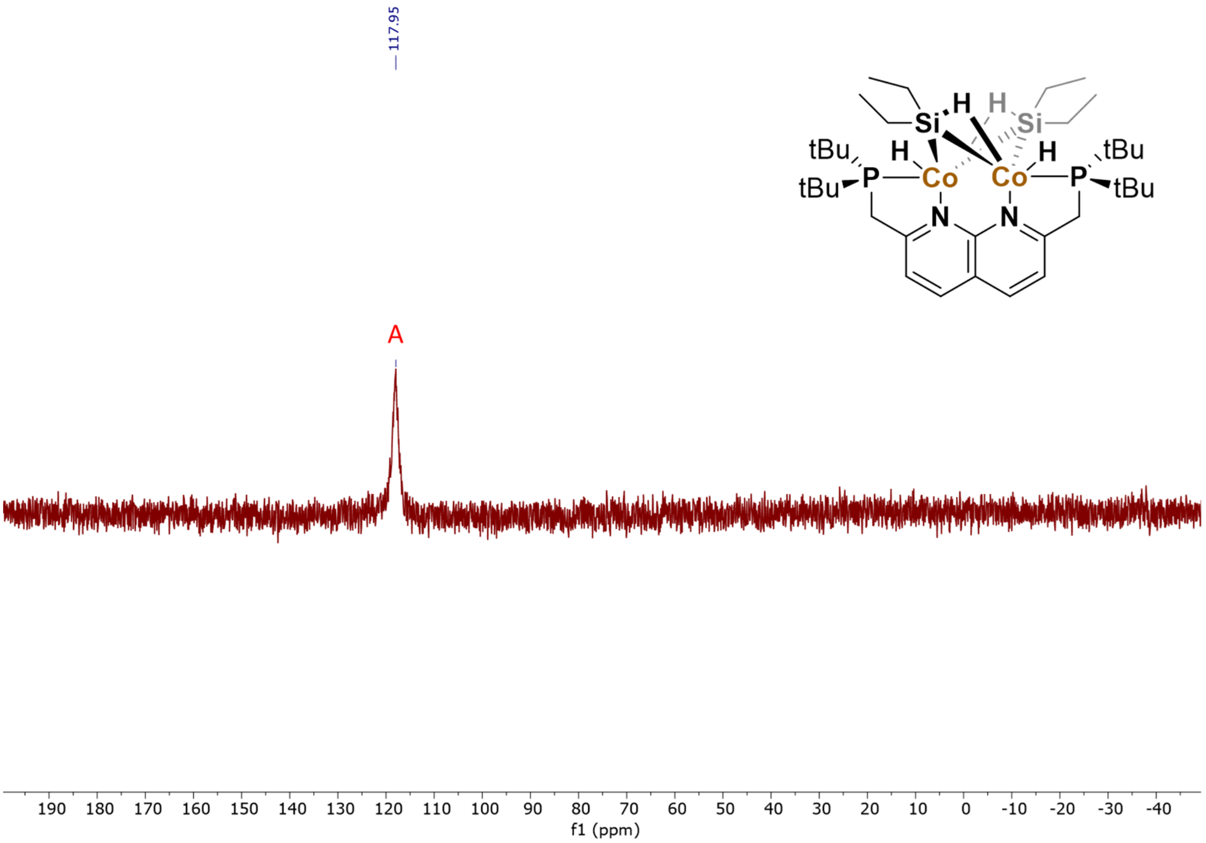

*Figure S9: ³¹P{¹H} NMR(298K, C_6D_6) of complex **3**.*

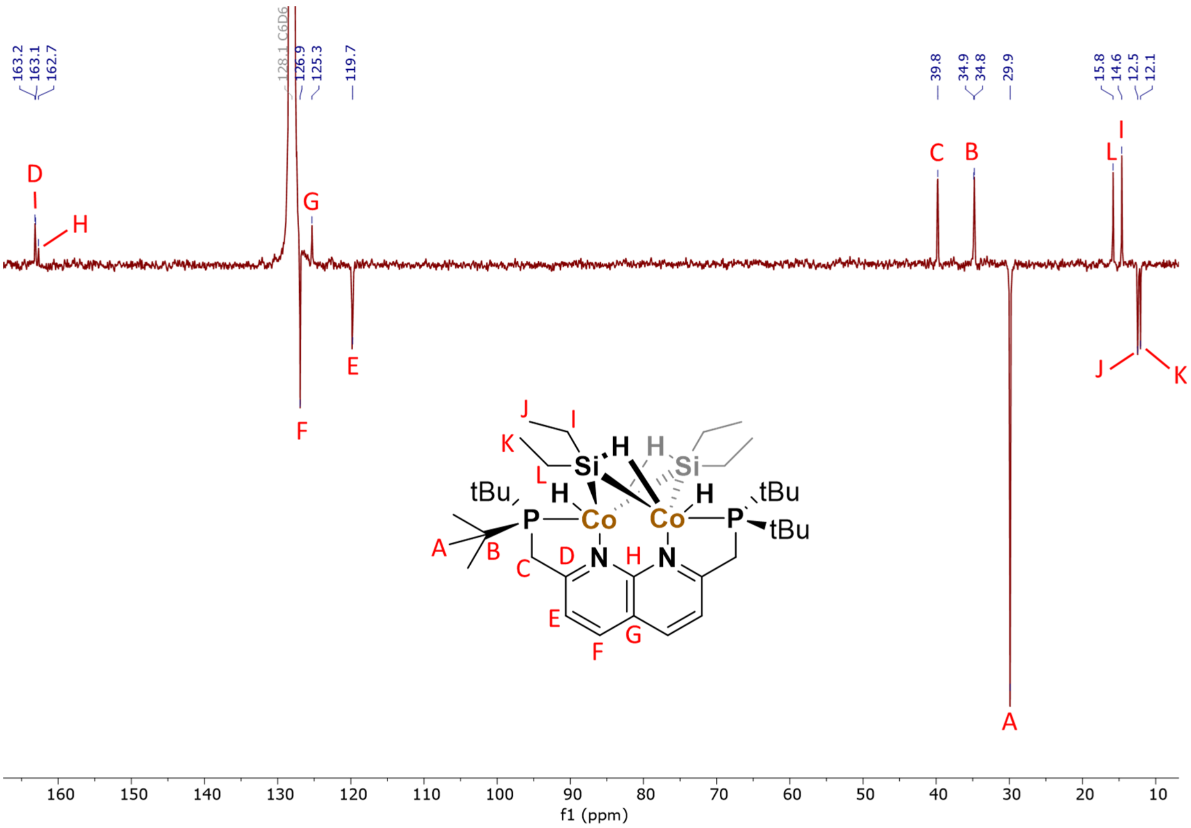

*Figure S10: $^{13}C\{^{1}H\}$ APT NMR(298K, C_6D_6) of complex **3**.*

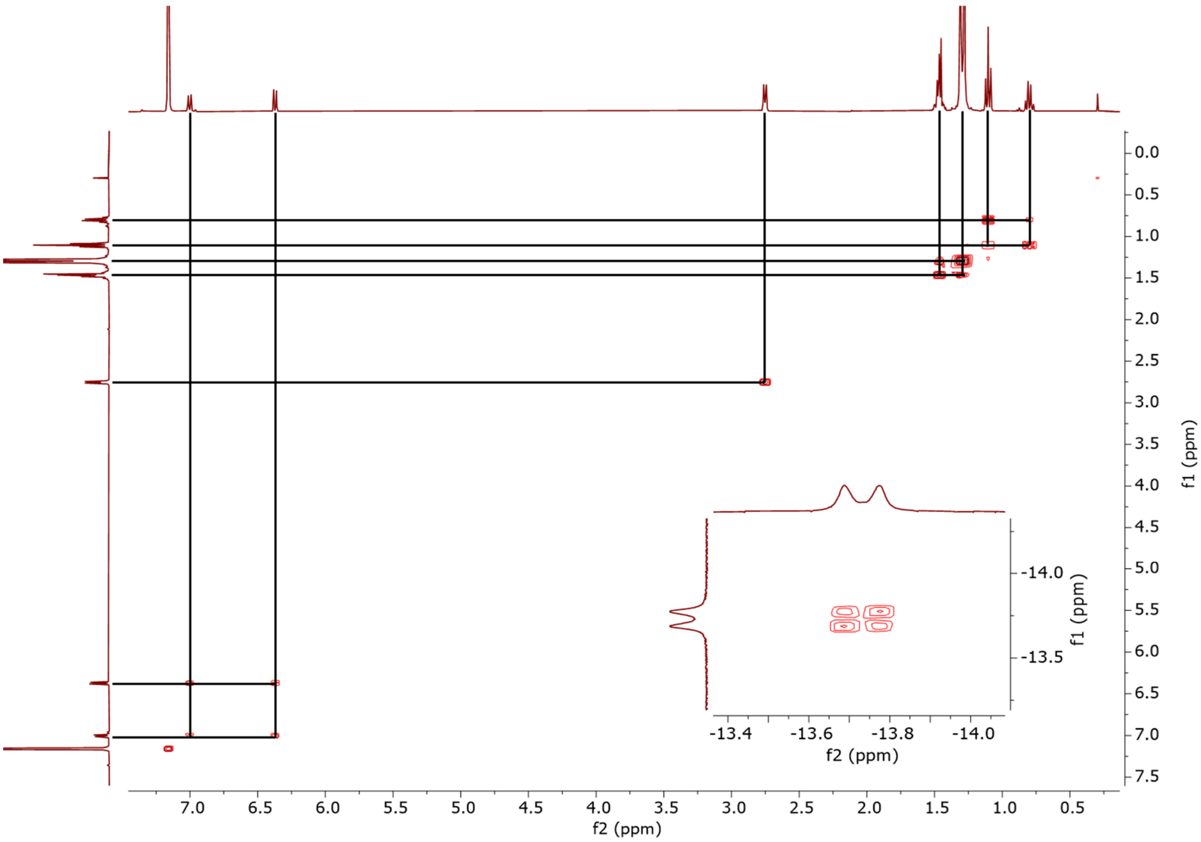

*Figure S11: ^{1}H-^{1}H COSY NMR(298K, C_6D_6) of complex **3**.*

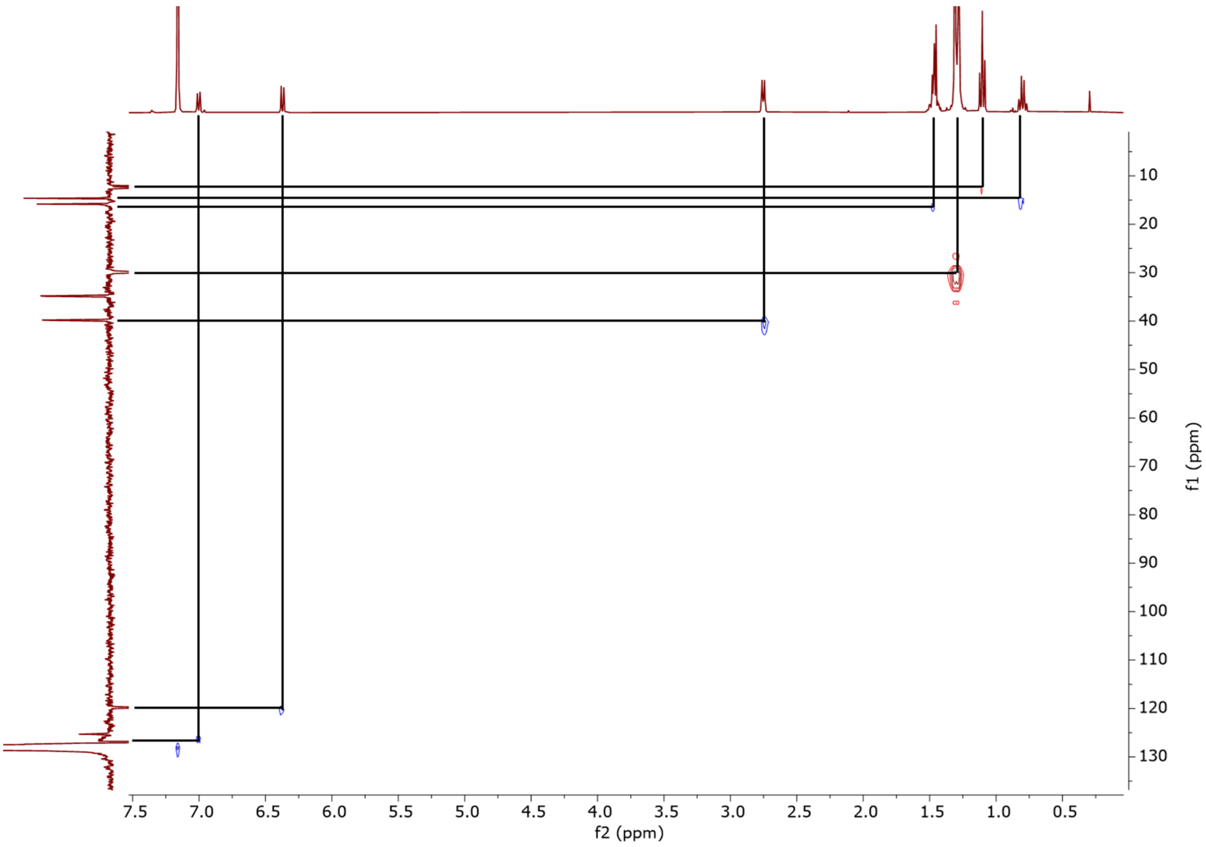

*Figure S12: ^{1}H-^{13}C HSQC NMR(298K, C_6D_6) of complex **3**.*

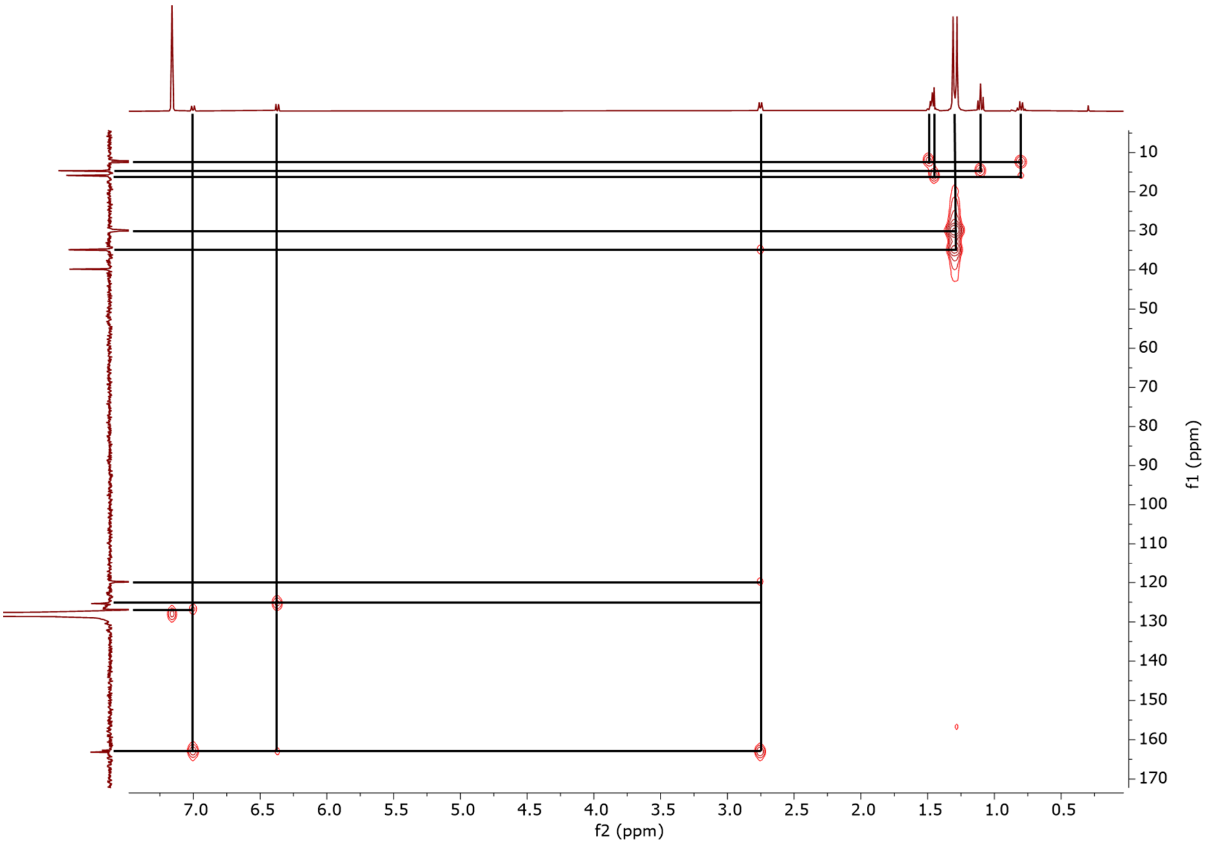

*Figure S13: ^{1}H-^{13}C HMBC NMR(298K, C_6D_6) of complex **3**.*

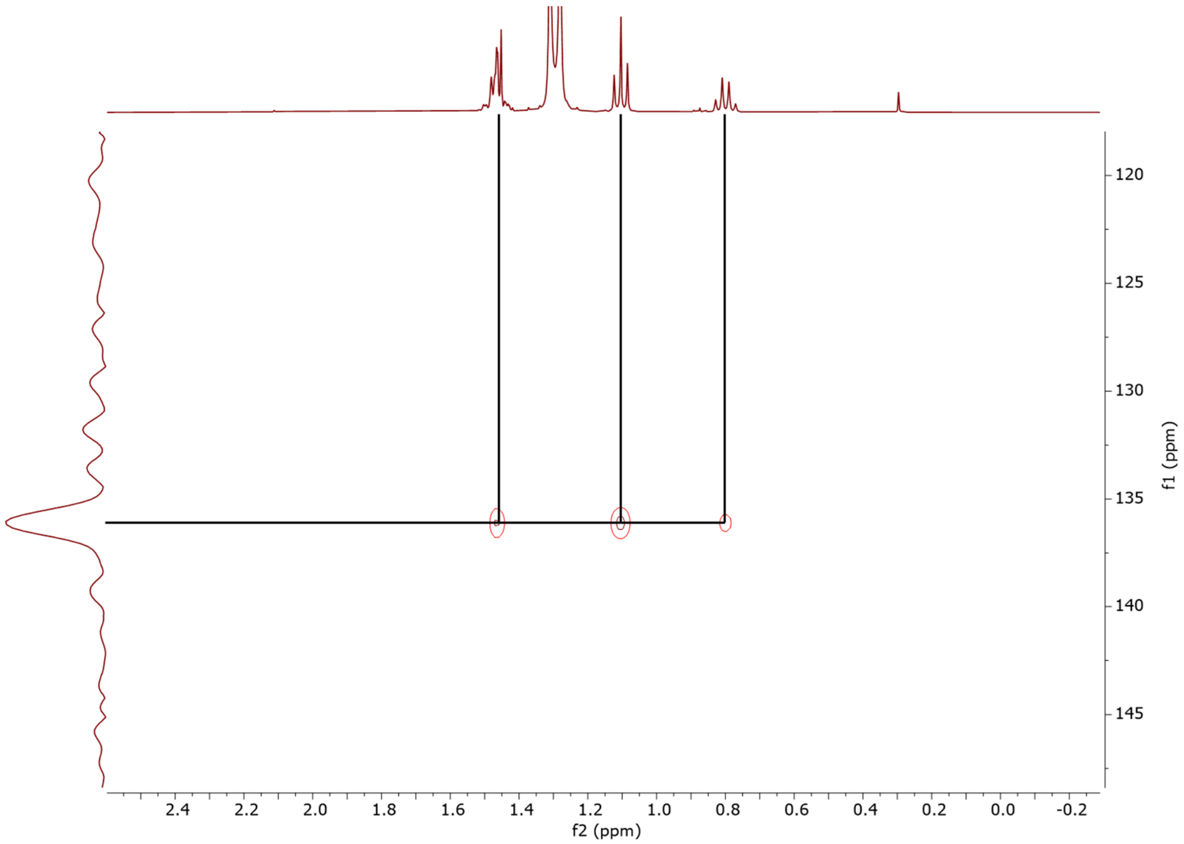

*Figure S14: ^{1}H-^{29}Si HMBC NMR(298K, C_6D_6) of complex **3**.*

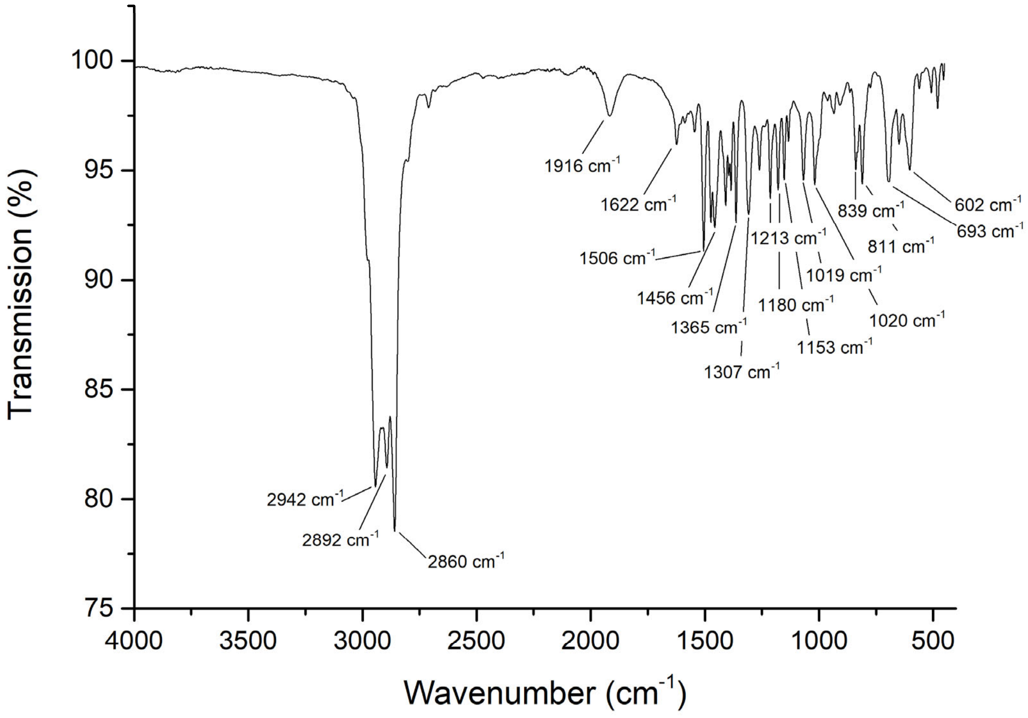

*Figure S15: FTIR (ATR) spectrum of complex **3**.*

VT NMR of complex 3

Complex **3** (5.3 mg) was dissolved in toluene-d_8 (0.6 mL) and sealed in a J-Young tube. The sample was first measured at 25°C, then cooled down to -70° and subsequently warmed up in steps of 20°C to 70°C. At low temperature there seems to be some broadening of the signals, however, since this is present for all peaks including the solvent, we ascribe this to suboptimal shimming of the machine at such low temperatures. During the warming of the sample, there are some minor impurities that appear which were not present in the starting material. In the ^{31}P spectra that were measured in this series as well, the appearance of an extra peak at around 35 ppm is observed. It is likely that these are impurities which originate from an imperfect seal of the J-Young valve at lower temperatures. Due to this, minor amounts of air may have gotten into the sample which explain the appearance of some minor (< 10%) side products.

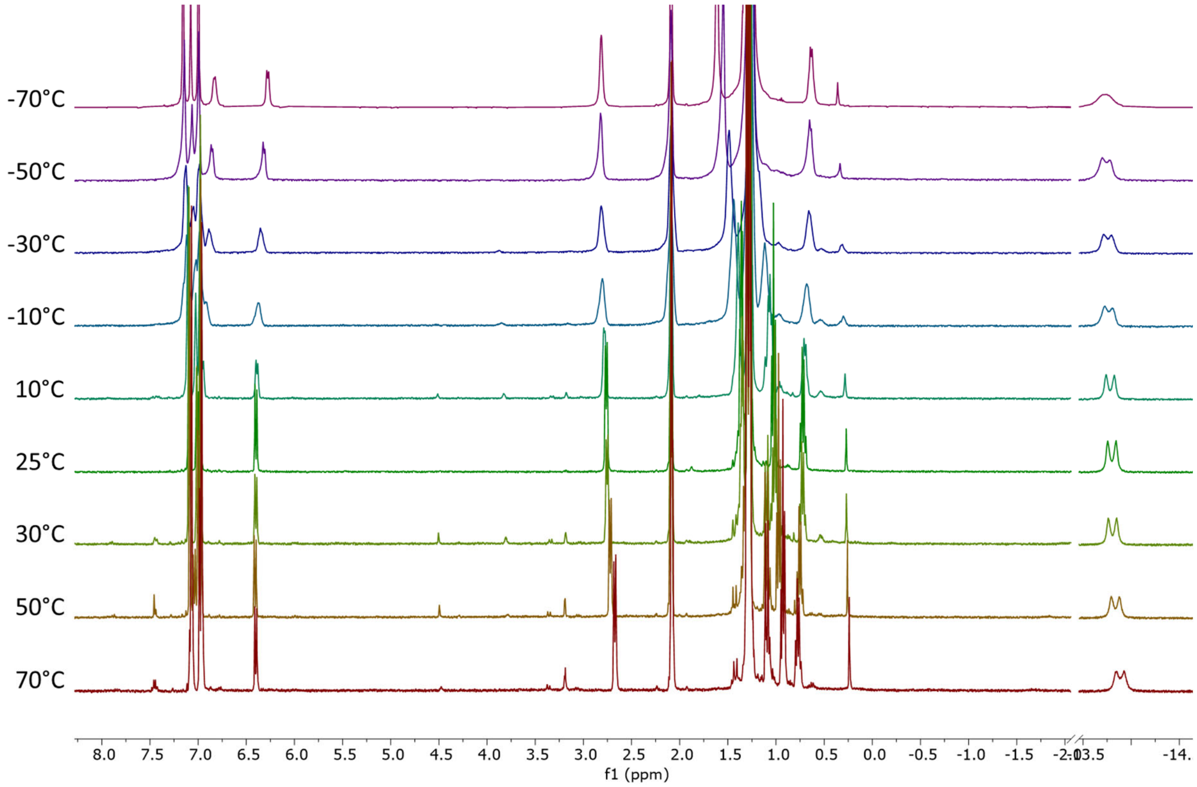

*Figure S16: VT ^1H NMR of complex **3** in toluene-d_8.*

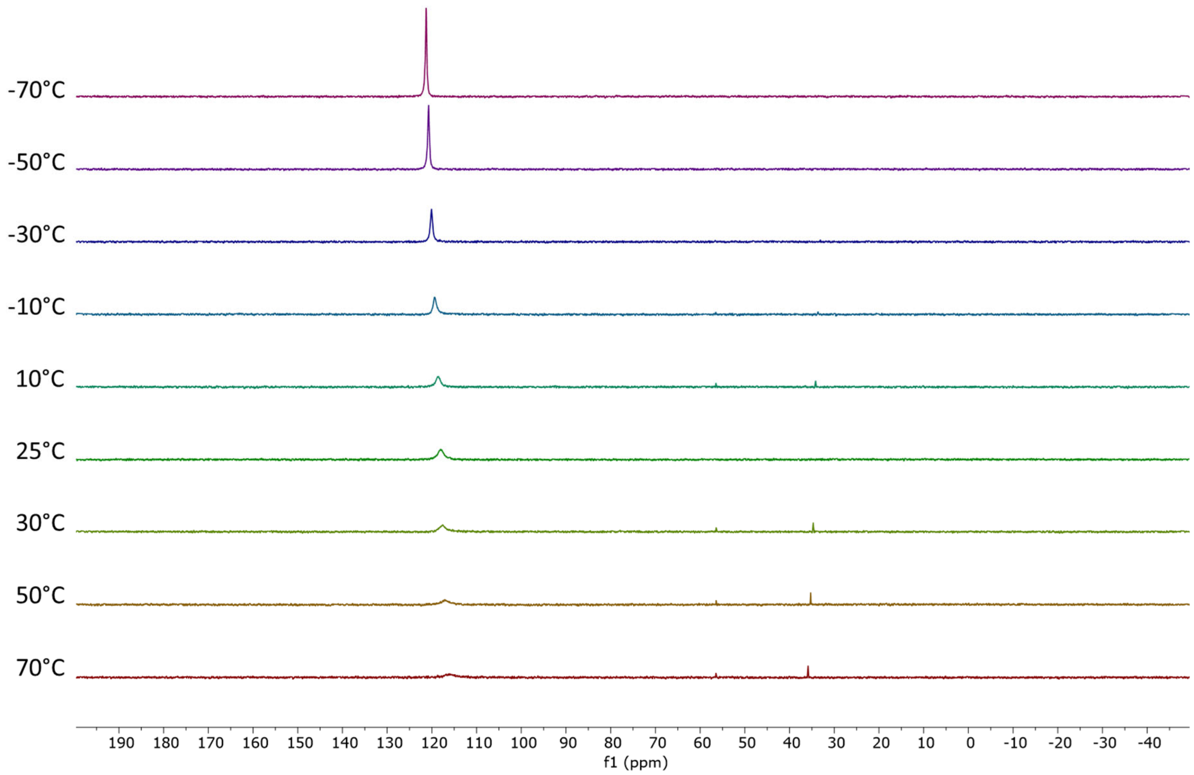

*Figure S17: VT ^{31}P NMR of complex **3** in toluene-d_8.*

Synthesis of [^{tBu}PNNP*$Co_2(SiEt_2)_2H_4$]K18-crown-6 (4)

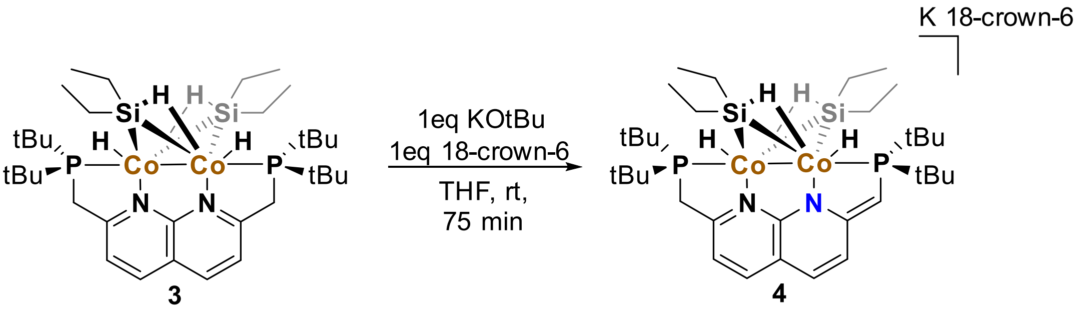

A solution of KOtBu (7.8 mg, 69.5 mmol, 1eq) and 18-crown-6 (18.4 mg, 69.6 mmol, 1eq) in THF (5.5 mL) was added dropwise to a solution of complex **3** (51.2 mg, 69.1 µmol) in THF (5.5 mL). During the addition, the color of the mixture changed from red brown to dark green. The mixture was stirred for 75 min after which the solvent was evaporated to yield a dark solid. The solid was suspended in hexane (6 mL) and filtered. The green residue was washed with hexane (3 times 2 mL) and subsequently extracted with THF (~4 mL). The THF extract was dried under a dynamic vacuum yielding **4** (62.8 mg, 77%) as a dark green powder.

^{1}H NMR (400 MHz, C_6D_6, 298 K): δ 6.27 (d, $^3J_{H,H}$ = 7.2 Hz, 1H), 6.15 (d, $^3J_{H,H}$ = 8.7 Hz, 1H), 5.93 (m, 2H), 4.25 (d, $^2J_{H,P}$ = 1.6 Hz, 1H), 4.25 (d, $^2J_{H,P}$ = 1.6 Hz, 1H), 3.61 (bs, 24H), 1.35 (d, $^3J_{H,P}$ = 10.9 Hz, 18H), 1.34 (d, $^3J_{H,P}$ = 11.1 Hz, 18H), 0.80 (m, 10H), 0.69 (m, 10H) ppm.

^{31}P NMR (162 MHz, C_6D_6, 298 K): δ 109.7, 108.3 ppm.

^{13}C NMR (101 MHz, C_6D_6, 298 K): δ 167.6 (d, $^2J_{C,P}$ = 24.6 Hz), 165.1 (s), 162.6 (d, $^2J_{C,P}$ = 11.4 Hz), 128.0 (s), 125.1 (s), 119.3 (d, $^3J_{C,P}$ = 15.6 Hz), 115.8 (s), 106.0 (d, $^3J_{C,P}$ = 8.9 Hz), 84.5 (d, $^1J_{C,P}$ = 27.4 Hz), 71.3 (s), 39.9 (d, $^1J_{C,P}$ = 4.7 Hz), 35.3 (d, $^1J_{C,P}$ = 12.6 Hz), 34.9 (d, $^1J_{C,P}$ = 6.2 Hz), 32.2 (d, $^2J_{C,P}$ = 6.3 Hz), 31.0 (d, $^2J_{C,P}$ = 6.1 Hz), 16.3 (bs), 15.2 (s), 12.3 (s) 12.2 (s) ppm.

^{29}Si NMR determined with HMBC (79 MHz, C_6D_6, 298 K): δ 127 ppm

IR-ATR (cm^{-1}): 2888(s), 2855(s), 1886(bw), 1611(w), 1529(w), 1494(m), 1409(m), 1351(w), 1109(s), 963(w).

Anal. Calc. for: C, 52.96; H, 8.79; N, 2.69. Found C, 46.53; H, 8.05; N, 2.25. The reactive nature of this compound precluded obtaining satisfactory elemental analysis.

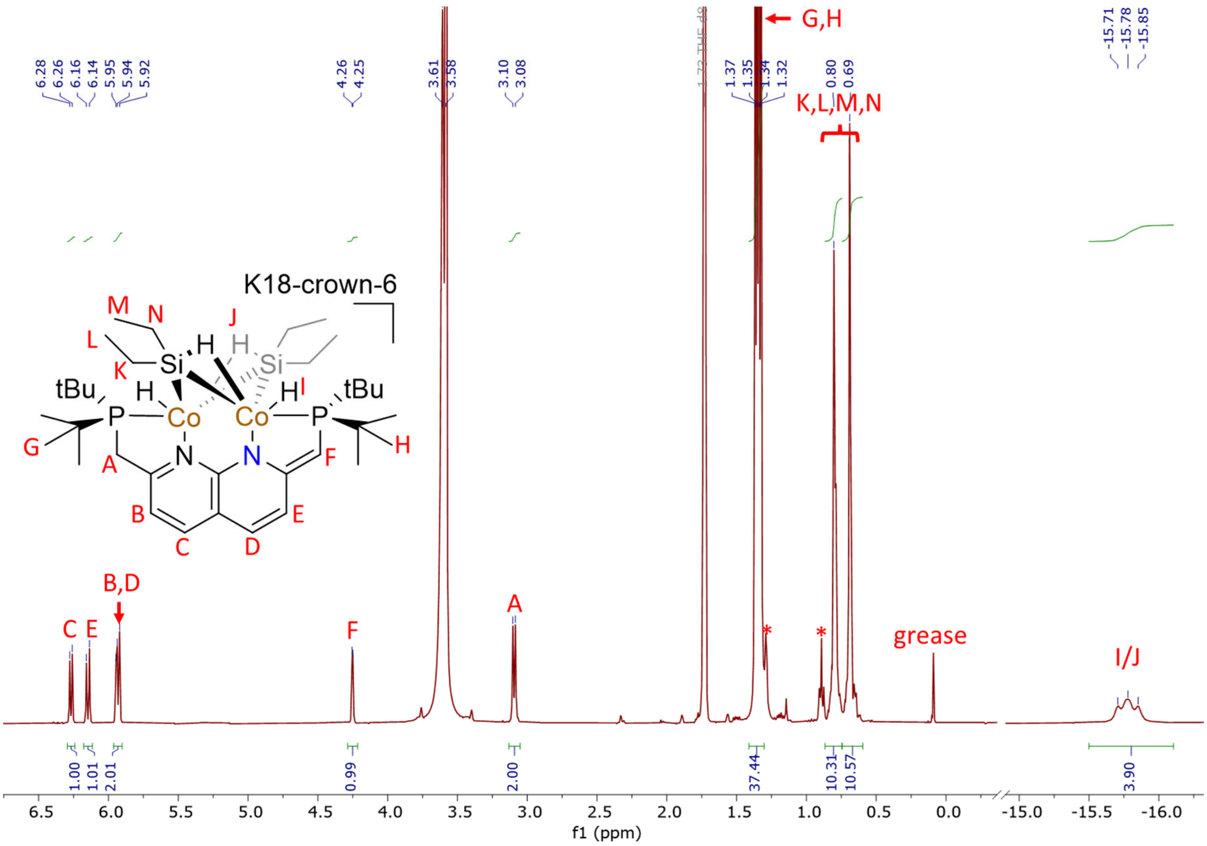

*Figure S18: Figure 1: ^{1}H NMR(298K, C_6D_6) of complex **4**.*

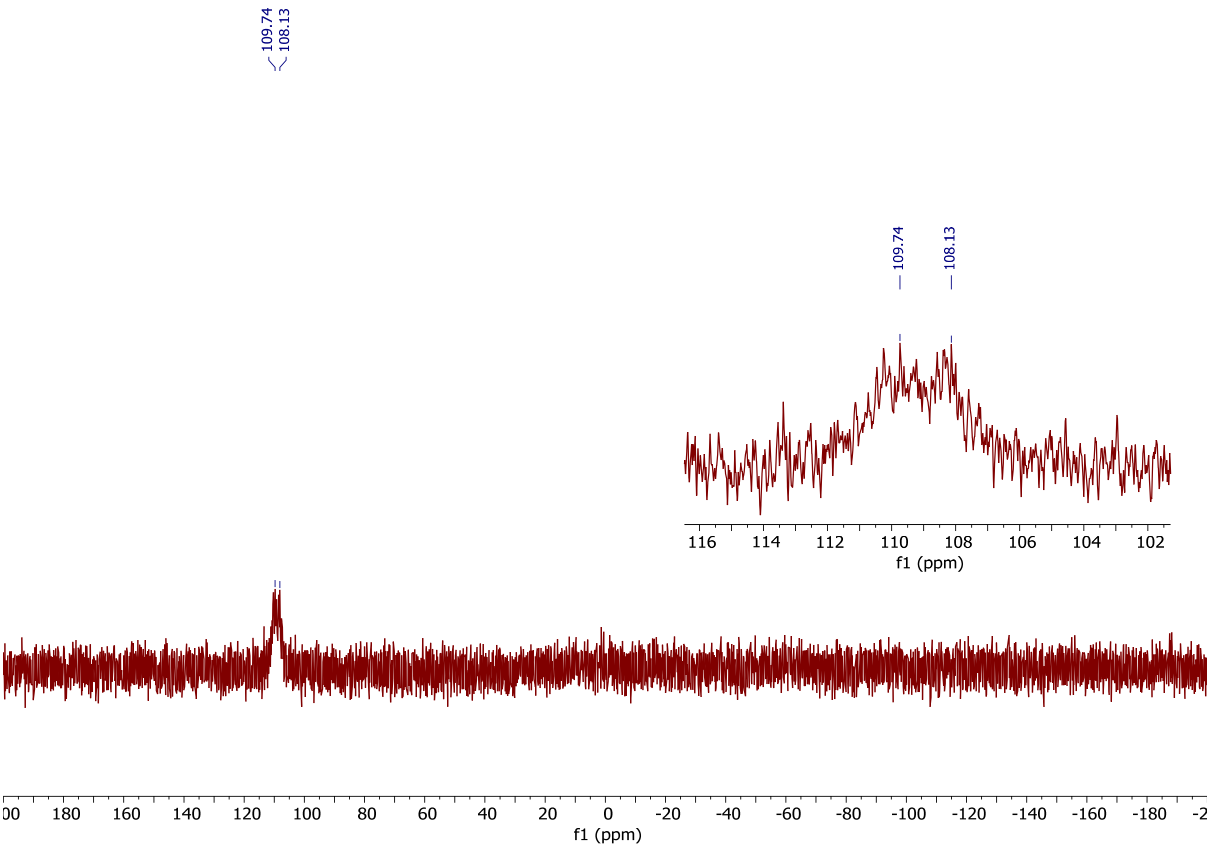

*Figure S19: $^{31}P\{^{1}H\}$ NMR(298K, C_6D_6) of complex **4**.*

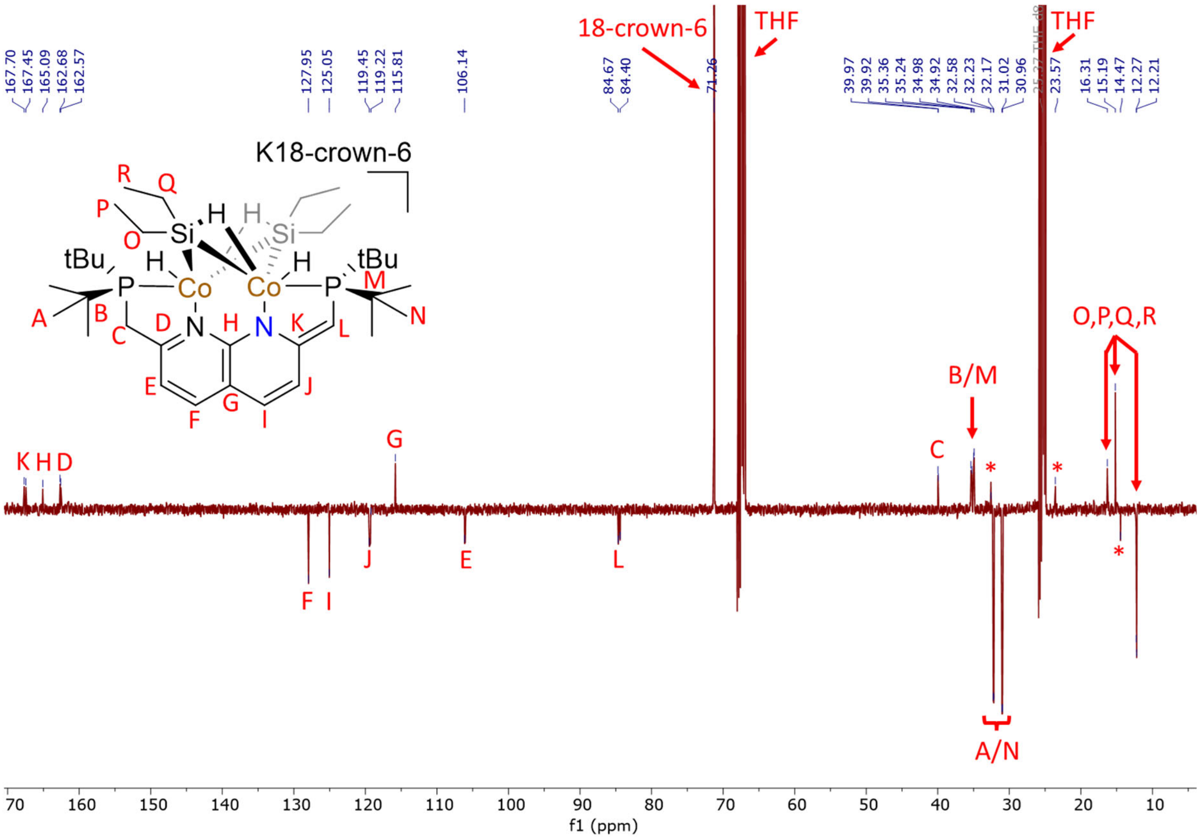

*Figure S20: $^{13}C\{^{1}H\}$ APT NMR(298K, C_6D_6) of complex **4**.*

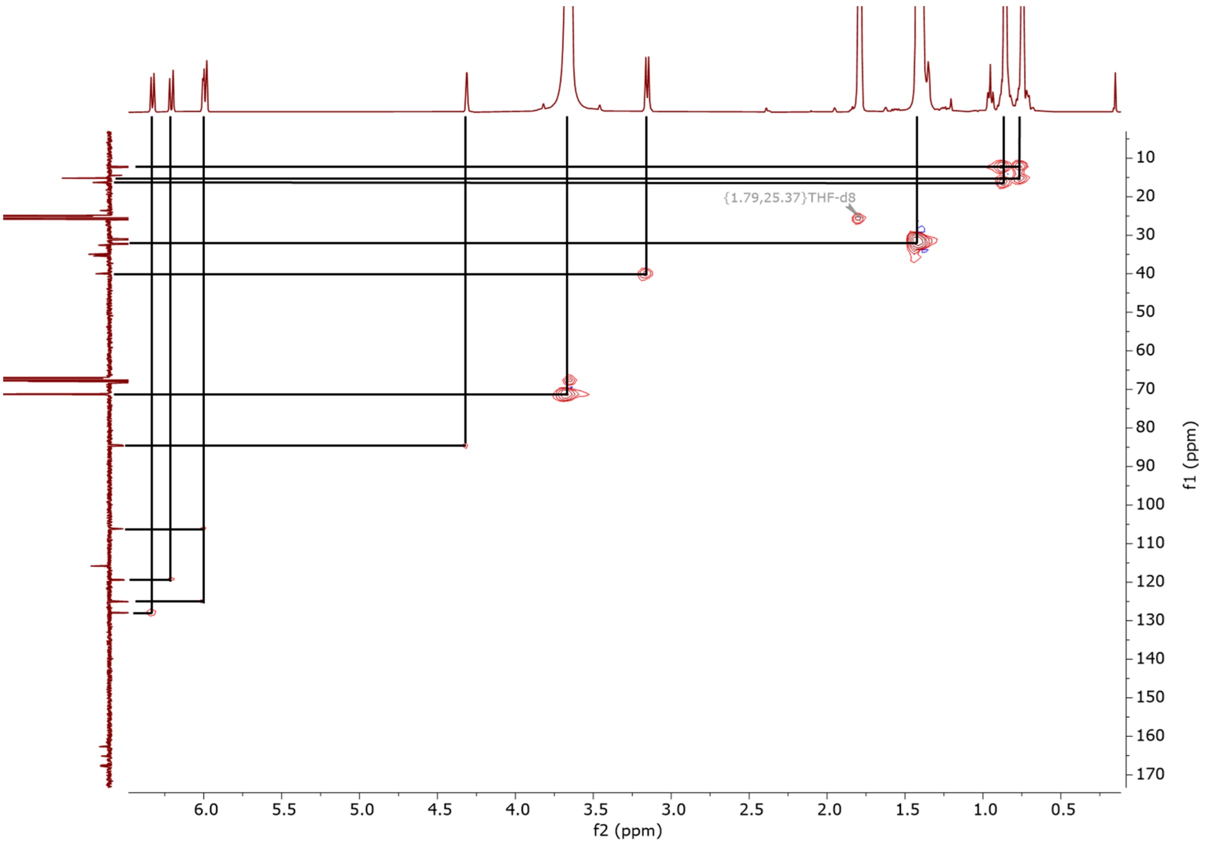

*Figure S21: ^{1}H-^{13}C ASAPHMQC NMR(298K, C_6D_6) of complex **4**.*

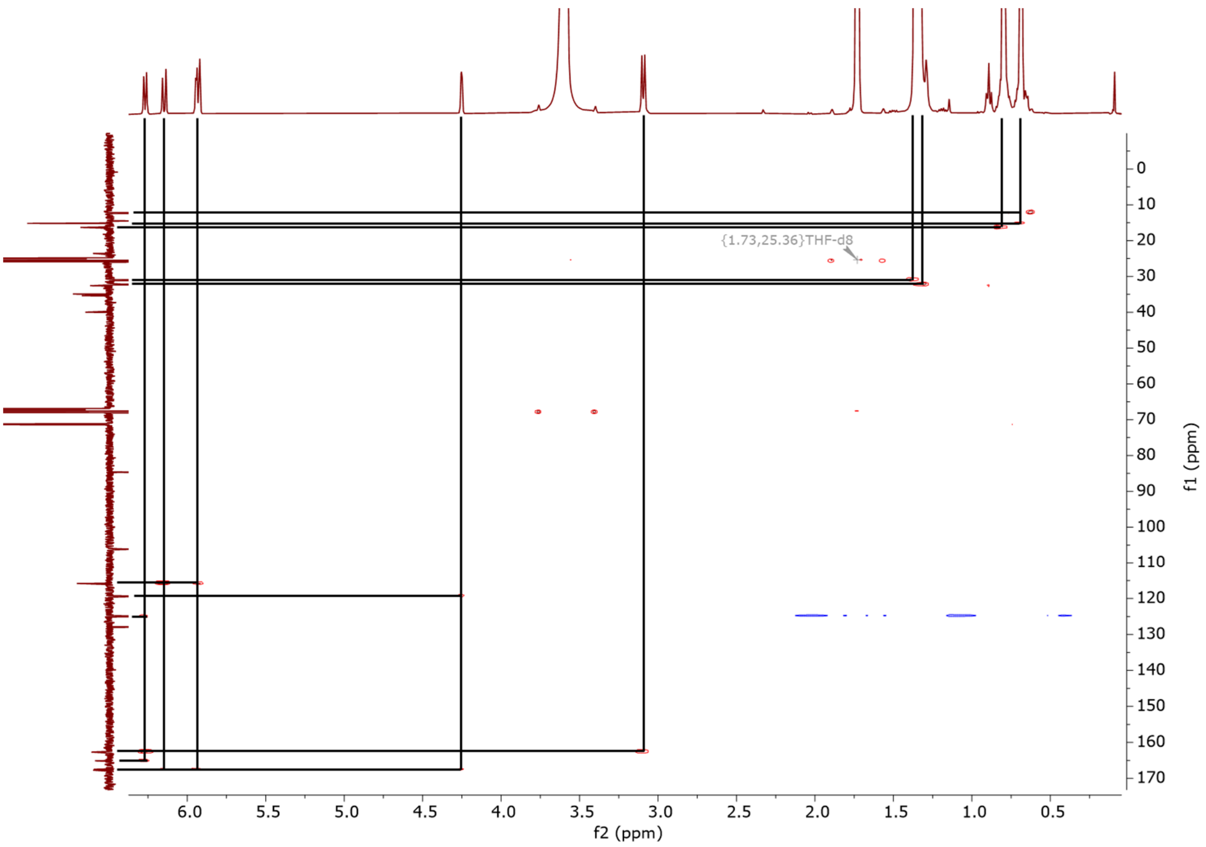

*Figure S22: ^{1}H-^{13}C HMBC NMR(298K, C_6D_6) of complex **4**.*

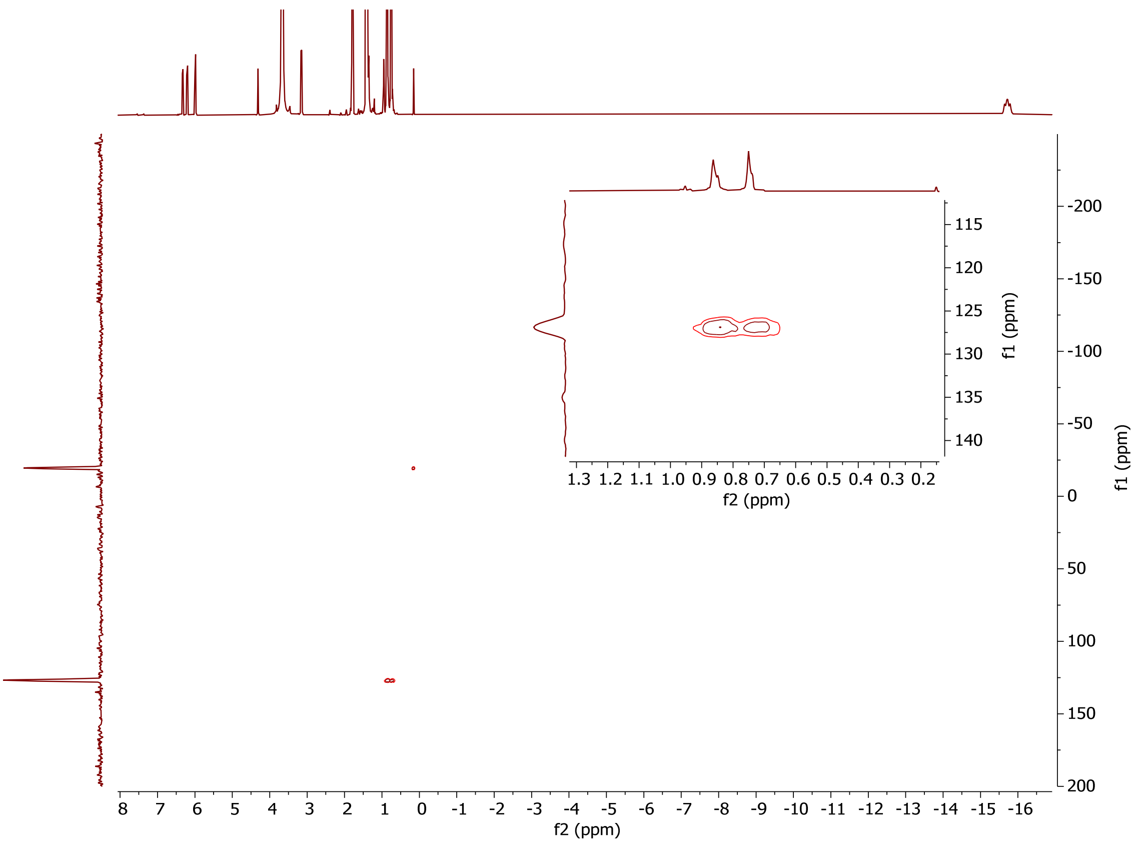

*Figure S23: ^{1}H-^{29}Si HMBC NMR(298K, C_6D_6) of complex **4** with a zoom-in of the cross-peak for the $SiEt_2$ fragments.*

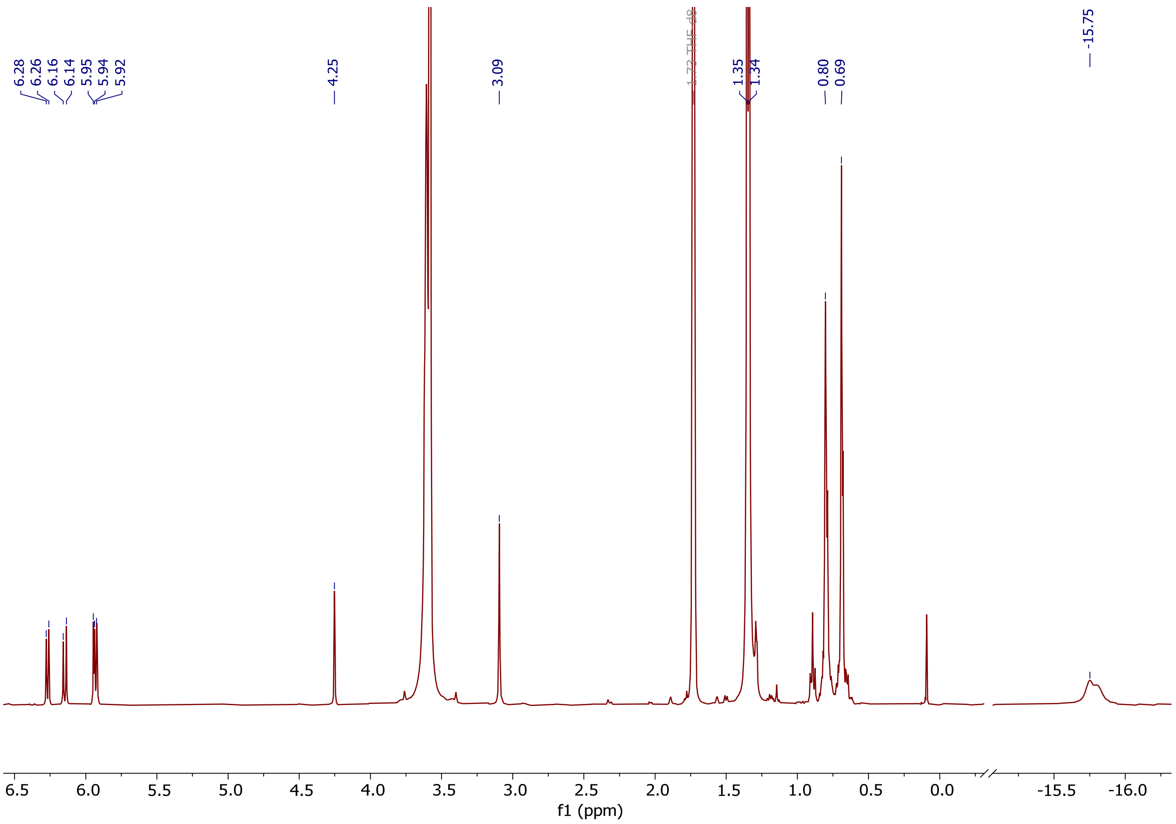

*Figure S24: $^{1}H\{^{31}P\}$ NMR(298K, C_6D_6) of complex **4**.*

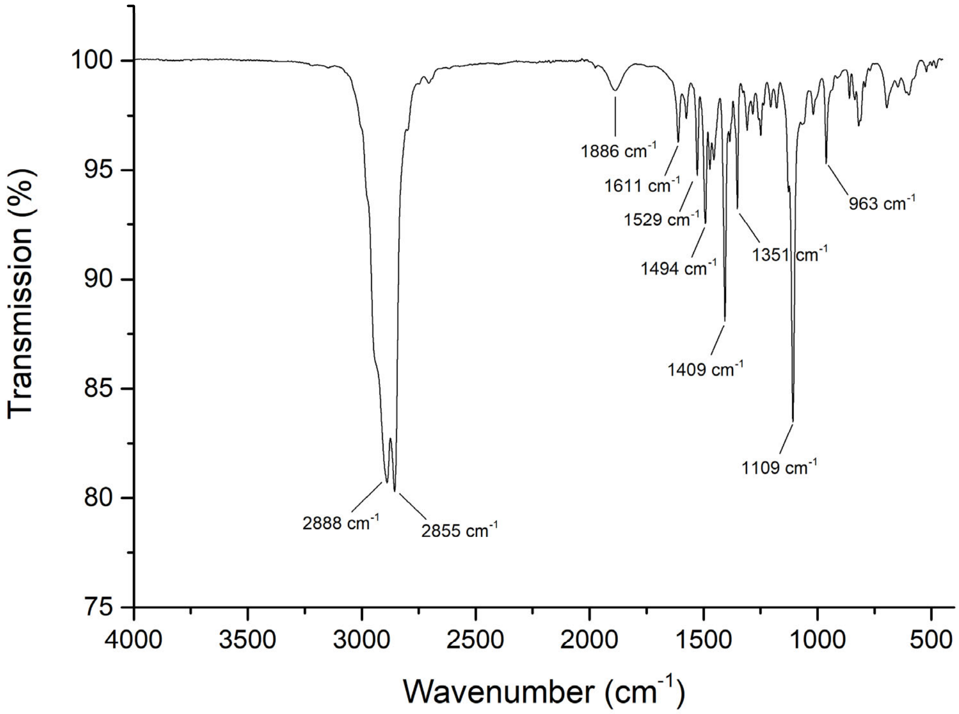

*Figure S25: FTIR (ATR) spectrum of complex **4**.*

Protonation of 4 with $HBArF_{24}$

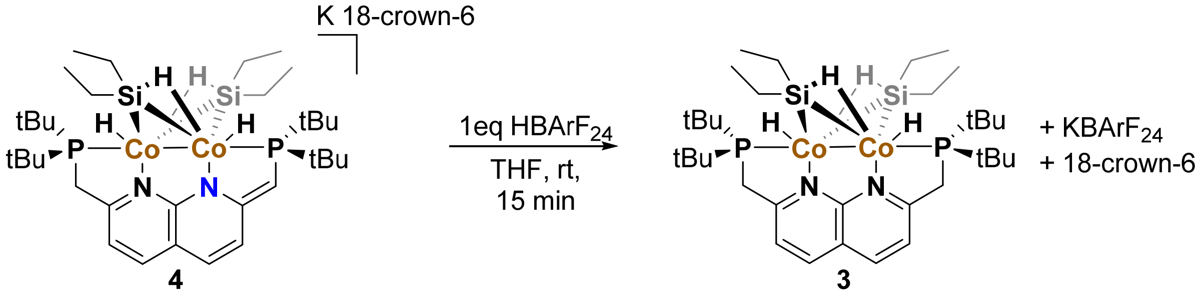

A freshly prepared solution of $HBArF_{24}$ (4.0 mg, 3.95 µmol, 1 eq) in THF (0.5 mL) was added dropwise to a solution of **4** (4.7 mg, 3.69 µmol, 1eq) in THF (0.5 mL). The solution changed color from dark green to brown. NMR signals are consistent with the formation of complex **3**.

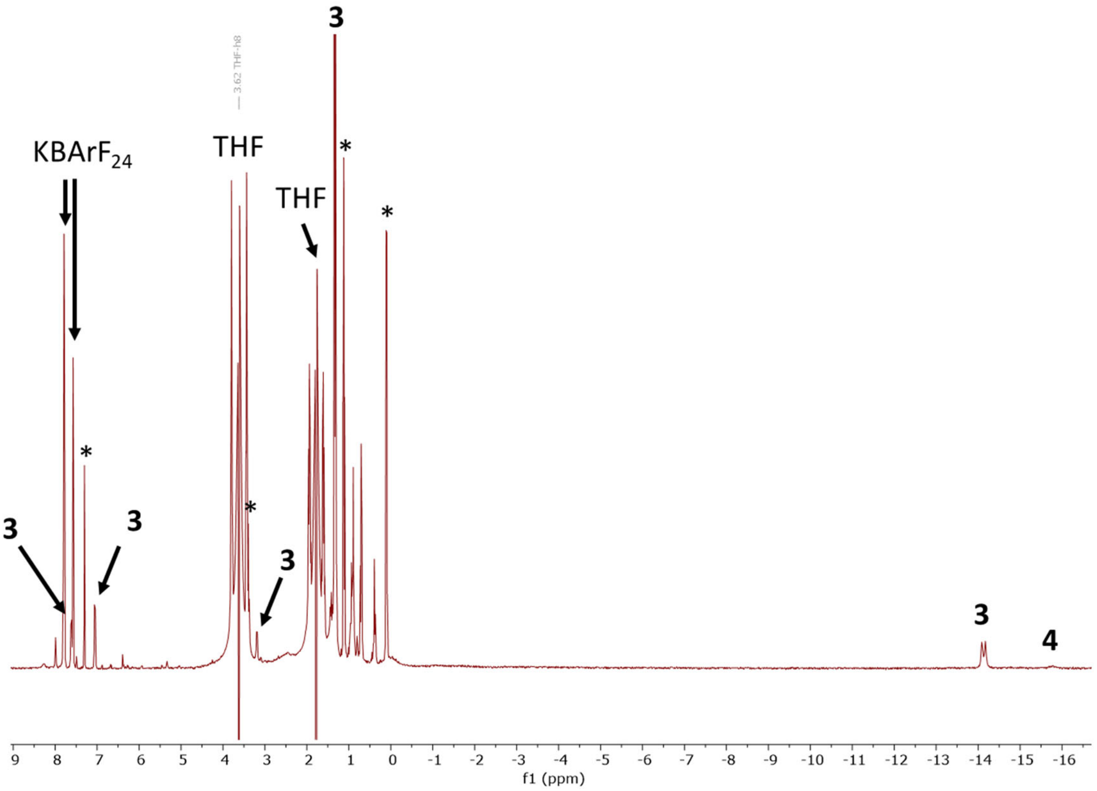

*Figure S26: Crude ^{1}H NMR (THF-h_8, 298K) spectrum of the reaction of **4** with $HBArF_{24}$ with PRESAT solvent suppression for the THF peaks. *is grease, benzene and diethyl ether.*

Tested reactions with 3 and 4

Table S1. Tested Reactivity for complexes **3** and **4**

#	Complex	Substrate	Result
1	**3**	PMe_3	No reaction at rt, decomposition at 50°C
2	**3**	Benzophenone imine	No reaction at 50°C
3	**3**	H_2SiPh_2	Substitution at 80°C
4	**3**	4-fluorophenyl acetylene	Slow reaction + decomposition at rt
5	**4**	4-fluorophenyl acetylene	Slow reaction + decomposition at rt
6	**3**	Diethylsilane + 4-fluoroanisole	No reaction at 80°C
7	**3**	H_2	No reaction at 80°C
8	**4**	1-octene	No reaction at rt
9	**4**	1-octene and CO_2	New unstable complex followed by decomposition
10	**4**	Benzonitrile and diethylsilane	No reaction
11	**4**	4-chlorobenzaldehyde	Decomposition
12	**4**	Butylacrylate and diethylsilane	Decomposition

Reaction of 3 with PMe_3

Complex **3** (4.4 mg, 5.9 µmol, 1eq) was dissolved in toluene-d_8 and a solution of 1M PMe_3 (10 µl, 10 µmol, 2 eq) was added. The mixture was sealed in a J-Young NMR tube. The sample was left at room temperature overnight, however, no reaction was observed with ^1H NMR. The sample was heated to 40°C for 3 hours after which no significant change was observed. The sample was heated to 50°C for 3 hours after which some new resonances between -4.5 ppm and -10 ppm were observed as well as a paramagnetic resonance around 70 ppm, indicating the formation of a mixture of new species.

Reaction of 3 with benzophenone imine

Complex **3** (4.4 mg, 5.9 µmol, 1eq) was dissolved in THF (0.6 mL) and benzophenone imine (1 µl, 5.96 µmol, 1eq) was added. After leaving the reaction at rt overnight, no change was observed in the ^1H NMR spectrum. The mixture was heated to 50°C for 24h, however, no change occurred in the NMR spectra.

Reaction of 3 with diphenylsilane

Complex **3** (4.8 mg, 6.5 µmol) was dissolved in C_6D_6 (0.5 mL) and diphenylsilane (12 µl, 65 µmol, 10 eq) was added. NMR showed no conversion of **3**. The sample was heated at 80°C for 15 hours, after which ~30% conversion of **3** into **3-EtPh** was observed evaluated by the integrals of the hydride resonances (-12.8 and -13.7 ppm respectively) (Figure 4). It might be that also some **3-Ph₂** had formed, however, since its peaks overlap with **3-EtPh** this is difficult to evaluate. The shape of the hydride peak assigned to some **3-EtPh**, does suggest that if there was any **3-Ph₂** present, it is a minor amount.

Reaction of 3 with 4-fluoroacetylene

Complex **3** (7.3 mg, 9.5 µmol, 1eq) was dissolved in THF (1 mL) and a THF solution of 0.35 M 4-fluorophenylacetylene (28 µl, 9.8 µmol, 1eq) was added to the solution with the complex. In NMR after leaving the mixture at room temperature overnight, mostly the signals of **3** were observed, however, a notable minor signal at - 3.97 ppm was visible with the same shape as the hydride peak from **3**. Leaving the sample at room temperature for 3 days led to the broadening of the resonances in 1H NMR indicative for decomposition into paramagnetic species.

Reaction of 3 with diethylsilane and 4-fluoroanisole

Complex **3** (4.7 mg, 6.3 µmol, 1 eq) was dissolved in toluene (0.7 mL) and transferred to a J-Young NMR tube. Diethylsilane (16 µl, 124 µmol, 20 eq) and 4-fluoroanisole (15 µl, 132 µmol, 20 eq) were added to the solution of **3**. No reaction was observed in NMR. The mixture was heated to 80°C for 3 days, however, no change was observed in NMR.

Reaction of 3 with H_2

Complex **3** (5.5 mg, 7.4 µmol) was dissolved in C_6D_6 (0.6 mL) and sealed in a J-Young NMR tube. The solution in the tube was degassed using 3 freeze-pump-thaw cycles after which it was backfilled with 1 atm of H_2 gas. The presence of H_2 gas was confirmed with ^1H NMR. The sample was heated to 40°C overnight but no change in the

^{1}H NMR spectrum was observed. The sample was heated at 80°C for 6 hours, however, no change was observed in NMR.

Reaction of 4 with 4-fluoroacetylene

Complex **4** (7.6 mg, 6.4 µmol, 1eq) was dissolved in THF (1 mL) and a THF solution of 0.35 M 4-fluorophenylacetylene (18 µl, 6.3 µmol, 1eq) was added to the solution with the complex. In NMR after leaving the mixture at room temperature overnight, mostly the signals of **4** were observed, however, a notable minor signal at - 3.24 ppm was visible with the same shape as the hydride peak from **4** similar to what was observed for the same reaction with complex **3**. We hypothesize that the new species might be one in which one of the silane moieties is exchanged for a coordinated alkyne. Leaving the mixture at room temperature for 3 additional days, however, did not lead to an increase in the peak associated with this potential new species but rather to the observation of broad signals associated with paramagnetic complexes.

Reaction of 4 with 1-octene and CO_2

From complex **3** (5.2 mg, 7 µmol, 1eq), complex **4** was synthesized according to the protocol described above. The product of the synthesis was dissolved in THF (0.6 mL) and 1-octene (2 µl, 13 µmol, 2eq) was added in a J-Young NMR tube. No reaction was observed after leaving the sample at room temperature overnight. The mixture was degassed with 3 freeze-pump-thaw cycles after which the tube was backfilled with 1 atm. of CO_2 upon which the colour of the solution changed from dark green to brown. NMR showed the formation of three new species in both ^{1}H and ^{31}P NMR. After leaving the mixture for 3 days, the original new species had disappeared, and new paramagnetically shifted signals (140 to -30 ppm) were observed in ^{1}H NMR.

Reaction of 4 with benzonitrile and diethylsilane

A stock solution of **4** (13.6 mg, 11.5 µmol, 1eq) and diethylsilane (112 µl, 0.86mmol, 75 eq) in THF (1.8 mL) was made. 0.5 mL of this stock solution was added to benzonitrile (25 µl, 0.24 mmol, 76 eq). After leaving the sample overnight at room temperature, no conversion of the complex was observed in ^{1}H NMR.

Reaction of 4 with 4-chlorobenzaldehyde

Complex **4** (4.0 mg, 3.4 µmol, 1eq) was dissolved in THF (0.6 mL) and was added 4-chlorobenzaldehyde (0.6 mg, 4.3 µmol, 1.3 eq). The colour instantly changed from green to dark orange. Directly after the addition, complex **4** had almost fully disappeared in the ^{1}H NMR spectrum and new broad resonances were observed in the diamagnetic region of the spectrum. Leaving the solution longer led to the further diminishing of the intensity of all signals in the spectrum.

Reaction of 4 with Butylacrylate

A stock solution of **4** (13.6 mg, 11.5 µmol, 1eq) and diethylsilane (112 µl, 0.86mmol, 75 eq) in THF (1.8 mL) was made. 0.5 mL of this stock solution was added to butylacrylate (34 µl, 0.24 mmol, 76 eq). The color changed from green to brown and eventually became red after 5 minutes. Three new resonances between -13.5 and -15.5 ppm were observed. These lost intensity over time in addition to the formation of new peaks in the aromatic region of the spectrum, indicating the formation of a complex mixture of species.

Computational methods

General considerations

Calculations were performed using Gaussian 16 rev. C01 software.[5] The Becke 3-parameter Lee-Yang-Parr (B3LYP) functional was used.[6,7] The redefinition of Ahlrichs triple-zeta split valence basis set (def2-TZVP) was used on all atoms, except for in the relaxed potential energy surface scan.[8] For all calculations, DFT-D3 dispersion correction with Becke-Johnson damping was used.[9,10] The Fukui functions were calculated by subtracting the electron density of **3** from the electron density of the radical anion of **3** using the MultiWFN program.[11] Visualization of the Fukui function was done in Jmol.[12] The starting geometry for the optimization of **3** was obtained from the coordinates of the solid-state structure. The starting geometry for **4** was obtained by removing a hydrogen atom from the structure of **3** and modifying the charge accordingly. The relaxed potential energy surface scan was performed with the redefinition of Ahlrichs split valence basis set (def2-SVP) instead of the def2-TZVP basis set used for all other optimizations. Quantum theory of atoms in molecules (QTAIM) calculations were performed using the MultiWFN program and Natural bonding orbital (NBO) calculations were performed with the NBO 6.0 software.[11,13]

Overlay calculated and measured structure of 3

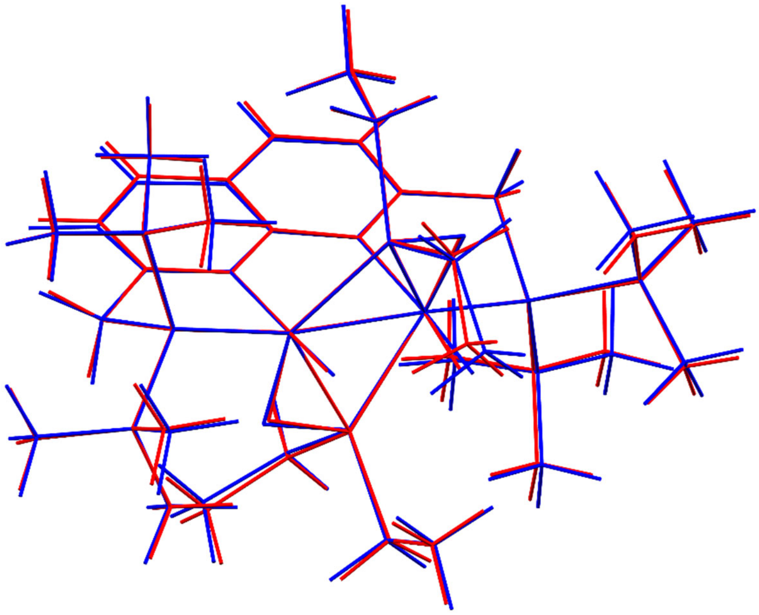

*Figure S27: The overlay of the solid-state structure of **3** (red) and the geometry optimized structure of **3** (blue).*

Fukui function of 3

Figure S28 shows that there is a clear electrophilic site on two of the methylene protons (H43 and H53), while there is no contribution of the Fukui function visible on silicon. In addition, a large part of the Fukui function is located on the backbone, which is consistent with what was previously observed for PNNP complexes. This does not mean that the backbone is prone to nucleophilic attack, but that in the case of increased electron density (for example due to deprotonation of the methylene positions) would be distributed mainly on the backbone, as was also previously found a PNNP copper hydride complex.[14]

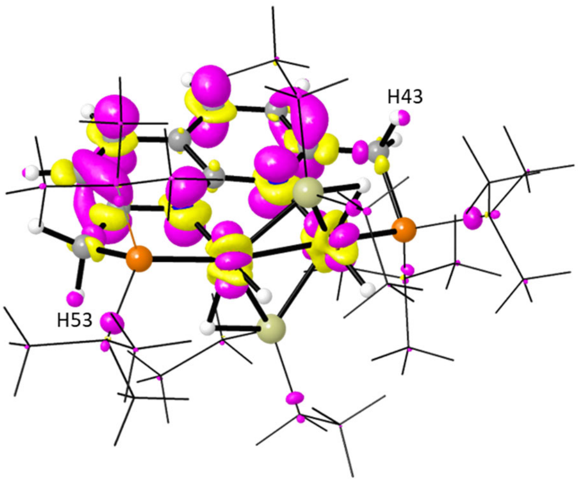

*Figure S28: The f_{n+1} Fukui function of complex **2**, showing the electrophilic sites in the molecule. tBu and Et groups are shown in wireframe for clarity. Purple shows the positive phase (i.e. increase of electron density upon addition of an electron) and yellow the negative phase.*

Hydride exchange mechanism of complex 3

To evaluate the feasibility of the hydride exchange mechanism proposed in figure 6, we performed a relaxed potential energy surface scan starting at the optimized structure of **3** in which the one of the terminal hydrides (H46) was moved towards one of the Si atoms (Si6) by decreasing the H46-Co1-N7 angle as shown in Scheme 4. The energy profile of this scan indicates the presence of a small energy barrier for this transformation as well as the presence of an intermediate (Figure S30). This intermediate was optimized and was indeed found to be a stationary structure as indicated by the absence of negative frequencies (Figure S30). In addition, the transition state (Figure S31) was also located and has a relative energy of 4.6 kcal/mol with respect to **3**. This indicates that the hydride exchange should be fast at room temperature, which is in line with the experimental results.

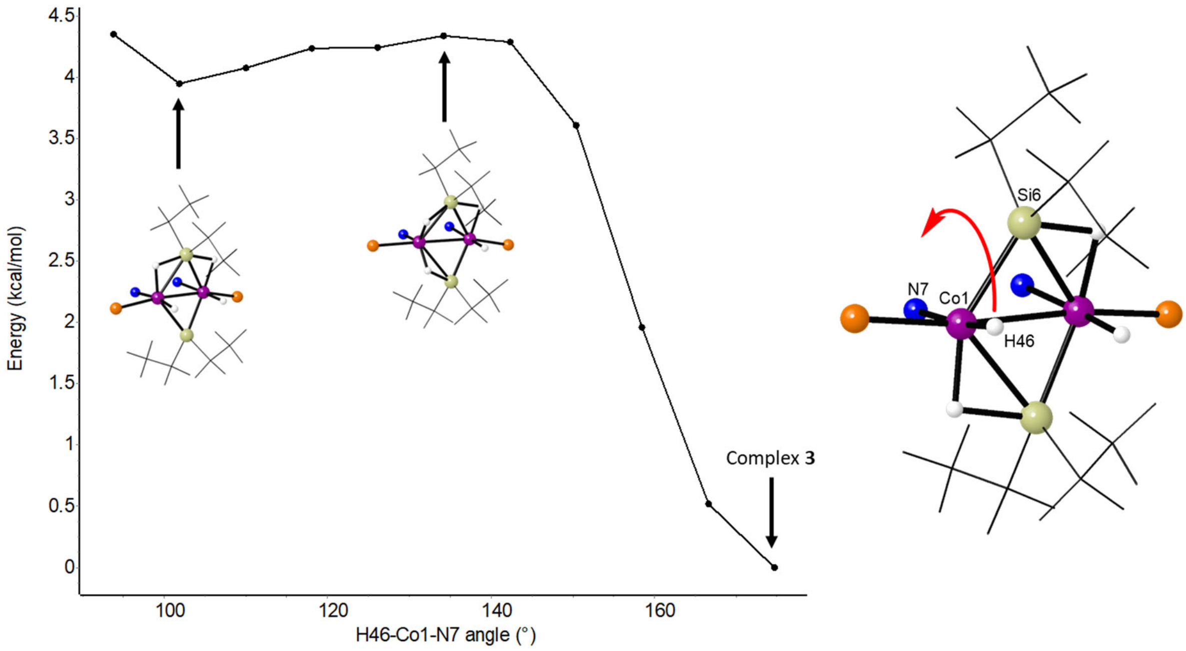

*Figure S29: The energy of the structures in the relaxed potential energy surface scan as a function of H46-Co1-N7 angle (left). The geometry around the core of the molecule for the highest energy (transition state like) structure and for the local minimum (intermediate like) structure are shown. The schematic representation of the potential energy surface scan shown on the core of **3** (right) in which the relevant atoms are labeled and where the arrow indicates the direction of the scan.*

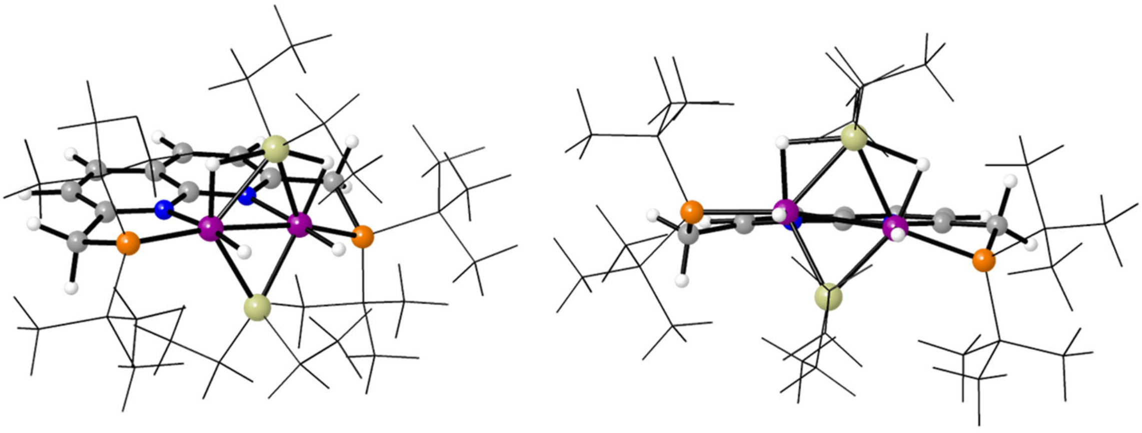

*Figure S30: The optimized structure of the intermediate for the hydride exchange mechanism in complex **3** in two perspectives. tBu and Et groups are drawn in wireframe for clarity.*

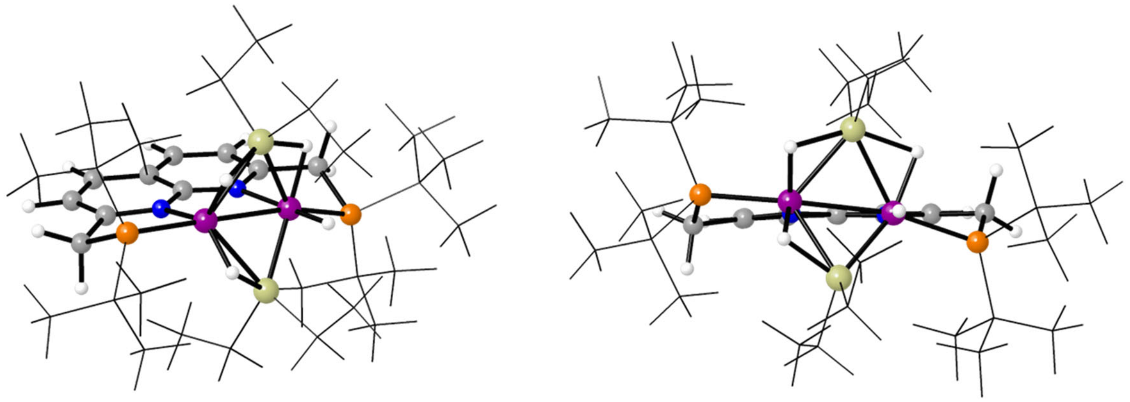

*Figure S31: The optimized structure of the transition state for the hydride exchange mechanism in complex **3** in two perspectives. tBu and Et groups are drawn in wireframe for clarity.*

Optimized structure of 4

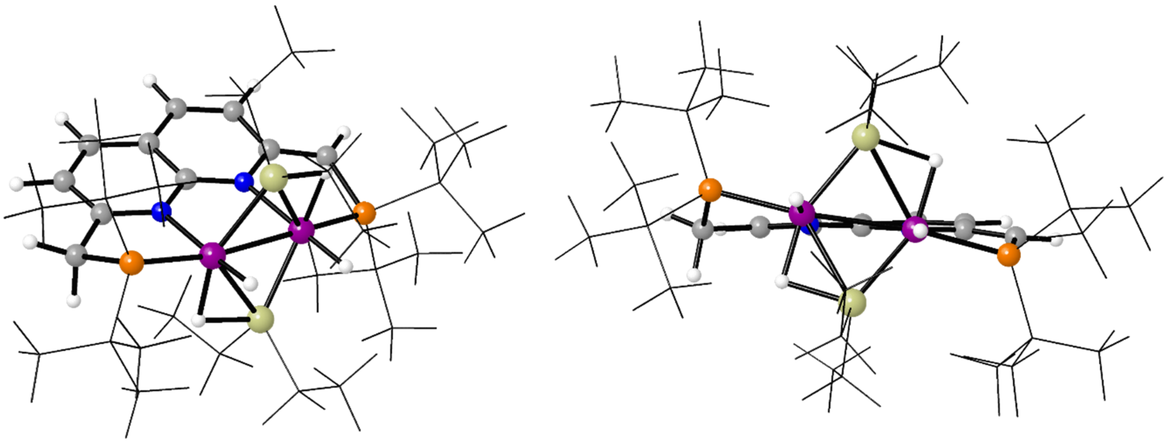

*Figure S32: Two views of the DFT optimized structure of complex **4**. tBu and Et groups are drawn in wireframe for clarity.*

QTAIM analysis of complex 3

QTAIM analysis of complex **3** shows that there are bond critical points (BCPs) between Co1-H45 and Si5-H45 both of which are located in a positive area of the Laplacian indicating a dative bonding interaction which is consistent with a base stabilized silylene description (R1, Scheme 5). There is no BCP between Si5 and Co1 although one would be expected in case of a bridging silylene (i.e. full oxidative addition of the Si-H bond). We interpret this as a result of the the partial silyl character (R2, Scheme 5) imposed by the base stabilized nature

of the silylene in this case, which weakens the Co1-Si5 interaction. For the other side of **3** (i.e. the Co2-Si6-H48 plane) the QTAIM analysis shows analogous results as expected based on the symmetry of the complex.

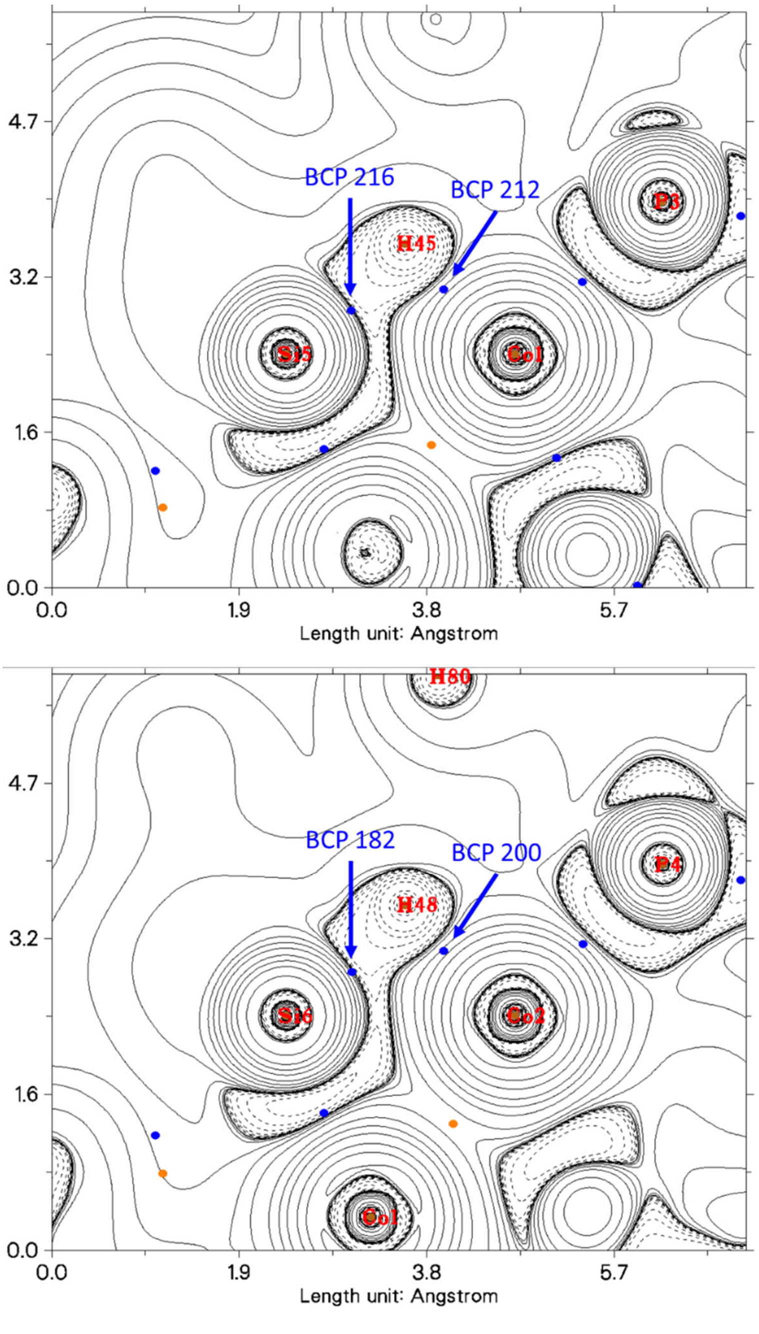

*Figure 33: The Laplacian of the electron density of complex **3** in the Co1-Si5-H45 plane (top) and the Co2-Si6-H48 plane (bottom). Bond critical points (BCP) are shown in blue, and ring critical points (RCP) are shown in orange. Solid lines indicate a positive area of the Laplacian and dashed lines indicate a negative area. The indicated BCPs have the following values for the Laplacian: BCP216 = 0.043, BCP212 = 0.255, BCP182 = 0.044, and BCP200 = 0.255.*

NBO analysis of complex 3

In the NBO analysis, the natural Lewis structure is the silyl resonance structure (R2, Scheme 5), which has a lower non-Lewis occupancy than the silylene Lewis structure (2.010% vs. 2.351%). Although it should be noted that in the silylene lewis structure, a lone-pair is located on H45 and H48 because the analogous structure with Co1-H45 and Co2-H48 could not be calculated with NBO and this could add to the higher non-Lewis occupancy of the silylene structure. Moreover, we noticed that NBO analysis of complex **3** in general is very sensitive to the precise geometry, and therefore to the level of theory used in the geometry optimization this warrants careful interpretation of the results.[15] In the natural population analysis, H45 and H48 (-0.20135 and -0.20383) are more

negatively charged than their cobalt hydride counterparts H46 and H50 (-0.01241 and -0.00668) which seems consistent with the QTAIM results of two hydrides which have a donating interaction towards Si and Co. In addition, the Wiberg bond indices indicate a weakened Si-H bonding (bond orders of 0.5633 and 0.5618). However, they also show only weak Co1-H45 and Co2-H48 bonding (0.1324 and 0.1286 respectively). Based on the Wiberg bond orders, the silicon atoms are bound asymmetrically between the cobalt centers with a bond orders of 0.4144 and 0.4118 to the Co center on the side of Si without the hydride and bond orders of 0.2085 and 0.2051 to the Co center on the side with the hydride. This is consistent with a combination of both the silyl and silylene resonance structures (R2 and R1 respectively, Scheme 5).

X-ray crystal structure determinations

X-ray crystal structure determination of 1

$C_{26}H_{44}Cl_4Co_2N_2P_2 \cdot CH_2Cl_2$, Fw = 791.16, blue needle, 0.37 × 0.05 × 0.04 mm^3, monoclinic, $P2_1/c$ (no. 14), a = 9.7176(6), b = 25.3618(13), c = 15.0924(9) Å, β = 98.470(3) °, V = 3679.1(4) Å^3, Z = 4, D_x = 1.428 g/cm^3, μ = 1.45 mm^{-1}. The diffraction experiment was performed on a Bruker Kappa ApexII diffractometer with sealed tube and Triumph monochromator (λ = 0.71073 Å) at a temperature of 150(2) K up to a resolution of $(\sin \theta/\lambda)_{max}$ = 0.61 Å^{-1}. The diffraction was weak and the crystal appeared to be cracked into several fragments. The three major fragments were integrated with the Eval15 software[16]. A multi-scan absorption correction and scaling was performed with TWINABS[17] (correction range 0.47-0.75). A total of 82550 reflections was measured, 7208 reflections were unique (R_{int} = 0.155), 3879 reflections were observed [$I>2\sigma(I)$]. The structure was solved with Patterson superposition methods using SHELXT[18]. Structure refinement was performed with SHELXL-2018[19] on F^2 of all reflections based on an HKLF-5 file[20]. Non-hydrogen atoms were refined freely with anisotropic displacement parameters. The CH_2Cl_2 solvent molecule was refined with a disorder model. All hydrogen atoms were introduced in calculated positions and refined with a riding model. 376 Parameters were refined with 41 restraints (geometry and displacement parameters of the disordered solvent). R1/wR2 [$I > 2\sigma(I)$]: 0.0832 / 0.1940. R1/wR2 [all refl.]: 0.1690 / 0.2315. Twin fractions of the second and third crystal fragment BASF = 0.347(4), 0.201(3). S = 1.057. Residual electron density between -0.96 and 1.22 e/Å^3. Geometry calculations and checking for higher symmetry was performed with the PLATON program.[21]

X-ray crystal structure determination of **2**

$C_{30}H_{52}Cl_2Co_2N_2OP_2$, Fw = 707.43, black block, 0.29 × 0.29 × 0.16 mm^3, monoclinic, $P2_1/c$ (no. 14), a = 12.8891(4), b = 12.8844(3), c = 21.5452(6) Å, β = 90.446(1) °, V = 3577.88(16) Å^3, Z = 4, D_x = 1.313 g/cm^3, μ = 1.19 mm^{-1}. The diffraction experiment was performed on a Bruker Kappa ApexII diffractometer with sealed tube and Triumph monochromator (λ = 0.71073 Å) at a temperature of 150(2) K up to a resolution of $(\sin \theta/\lambda)_{max}$ = 0.65 Å^{-1}. The crystal appeared to be cracked, with a rotation angle of 3.2 ° between the two crystal fragments. Consequently, two orientation matrices were used for the intensity integration with the Eval15 software[16]. A multi-scan absorption correction and scaling was performed with TWINABS[17] (correction range 0.59-0.75). A total of 50460 reflections was measured, 8875 reflections were unique (R_{int} = 0.049), 7197 reflections were observed [$I>2\sigma(I)$]. The structure was solved with Patterson superposition methods using SHELXT[18]. Structure refinement was performed with SHELXL-2018[19] on F^2 of all reflections based on an HKLF-5 file[20]. Non-hydrogen atoms were refined freely with anisotropic displacement parameters. All hydrogen atoms were located in difference Fourier maps and refined with a riding model. 368 Parameters were refined with no restraints. R1/wR2 [$I > 2\sigma(I)$]: 0.0419 / 0.1064. R1/wR2 [all refl.]: 0.0557 / 0.1151. Twin fraction of the second crystal fragment BASF = 0.3124(15). S = 1.014. Residual electron density between -0.36 and 0.67 e/Å^3. Geometry calculations and checking for higher symmetry was performed with the PLATON program.[21]

X-ray crystal structure determination of **3**

$C_{34}H_{68}Co_2N_2P_2Si_2$, Fw = 740.88, black plate, 0.20 × 0.17 × 0.04 mm^3, orthorhombic, Pbca (no. 61), a = 15.4152(4), b = 21.2750(5), c = 23.6074(6) Å, V = 7742.3(3) $Å^3$, Z = 8, D_x = 1.271 g/cm^3, μ = 1.03 mm^{-1}. The diffraction experiment was performed on a Bruker Kappa ApexII diffractometer with sealed tube and Triumph monochromator (λ = 0.71073 Å) at a temperature of 150(2) K up to a resolution of $(\sin \theta/\lambda)_{max}$ = 0.61 $Å^{-1}$. The crystal appeared to be cracked into several fragments. The Eval15 software[16] was used for the intensity integration using only the major crystal fragment. The prediction of reflection profiles involved a split-mosaic model. A multi-scan absorption correction and scaling was performed with SADABS[22] (correction range 0.63-0.75). A total of 73761 reflections was measured, 7208 reflections were unique (R_{int} = 0.098), 5191 reflections were observed [I>2σ(I)]. The structure was solved with Patterson superposition methods using SHELXT.[18] Structure refinement was performed with SHELXL-2018[19] on F^2 of all reflections. Non-hydrogen atoms were refined freely with anisotropic displacement parameters. Hydrogen atoms H1M-H4M were located in difference Fourier maps and kept fixed on these positions. All other hydrogen atoms were introduced in calculated positions and refined with a riding model. 395 Parameters were refined with no restraints. R1/wR2 [I > 2σ(I)]: 0.0395 / 0.0805. R1/wR2 [all refl.]: 0.0714 / 0.0919. S = 1.076. Residual electron density between -0.36 and 0.56 e/$Å^3$. Geometry calculations and checking for higher symmetry was performed with the PLATON program.[21]

CCDC 2329793 (compound **1**), 2329794 (compound **2**) and 2329795 (compound **3**) contain the supplementary crystallographic data for this paper. These data can be obtained free of charge from The Cambridge Crystallographic Data Centre via www.ccdc.cam.ac.uk/data_request/cif.

EXAFS measurements

General considerations

Co K-edge X-ray absorption spectra were measured at the Stanford Synchrotron Radiation Lightsource (SSRL) on the unfocused 20-pole 2T wiggler side-station beam line 7-3 under standard ring conditions of 3 GeV and ~500 mA. A Si(220) double crystal monochromator was used for energy selection with crystal orientation ϕ = 0°. Bulk solid samples were stored and handled in a dry N_2 glovebox. Samples were prepared by grinding ~5 mg of sample (mass calculated to yield ~1 absorption length above the Co K-edge) with ~25 mg dry boron nitride in an agate mortar and pestle to form a uniformly colored, fine powder. The powder was pressed into a 7 mm diameter cylindrical pellet and held between 64 µm Kapton tape. During data collection, the sample was held in a Cryo Industries closed cycle liquid He cryostat and maintained at ~10 K throughout the measurement. Spectra were measured to k = 14.1 $Å^{-1}$ in transmission mode using N_2-filled ionization chambers. The X-ray beam size was 1.2 mm (height) × 4 mm (width) for all measurements. A Co foil was measured simultaneously for energy calibration, and the first inflection point in the Co foil spectrum was fixed at 7709 eV.

Data processing was performed in the Athena program of the Demeter package.[23] Data presented here were obtained by aligning and merging two scans on different sample spots to ensure minimal beam damage. The post-edge EXAFS background was modeled in the Pyspline program using a three-region spline of orders 2, 3, and 3.[24] EXAFS was modeled using the Artemis program of the Demeter package. Theoretical EXAFS signals $\chi(k)$ were calculated using FEFF6. Absorber-backscatter paths were generated from the atomic coordinates using DFT-optimized structures. The EXAFS model was optimized in k-space using k^1, k^2, and k^3 weightings with the model obeying the Nyquist criterion.[25] EXAFS fits were performed between a k-range of 2-14 $Å^{-1}$ and varying R-ranges. Structural parameters varied during the fitting process were the bond distance (R) and bond variance (σ^2). The non-structural parameter ΔE_0 (E_0 is the energy at which k equals 0) was also fit. Coordination numbers were systematically varied over the course of the fitting process to assess different models but were fixed during a given fit. The value of S_0^2 was set to 0.9 for all fits.

EXAFS fit results for complexes **1-4**

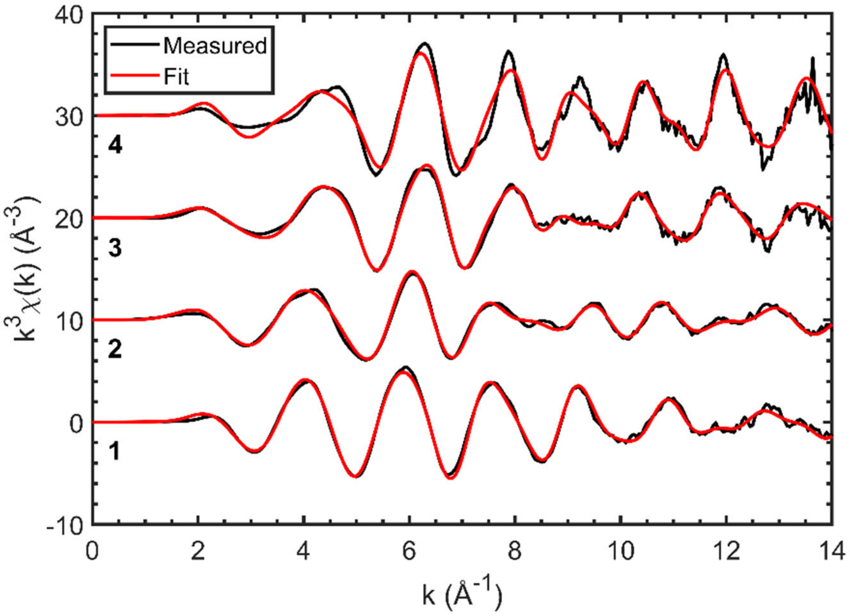

Figure 34: k^3-weighted Co K-edge EXAFS of dicobalt complexes. Spectra were fit in a k-range of 2-14 Å^{-1} and differing R-ranges depending on the scattering paths included in the model.

Table S2: EXAFS fit results for dicobalt compounds.

Compound	Path	CN	R (Å)	$10^5 \times \sigma^2$ (Å^2)	ΔE_0 (eV)	R-factor
**1**	Co-N	0.5	1.94 ± 0.03	101 ± 214	-7.4 ± 1.1	0.012
	Co-Cl[a]	2	2.22 ± 0.01	116 ± 49		
	Co-Cl[a]	1.5	2.35 ± 0.01	52 ± 60		
	Co-Co	1	3.85 ± 0.01	475 ± 128		
**2**	Co-O	1	1.92 ± 0.03	167 ± 168	-5.6 ± 2.9	0.017
	Co-N	1	2.10 ± 0.12	120 ± 1731		
	Co-Cl	1	2.22 ± 0.09	395 ± 2077		
	Co-P	1	2.37 ± 0.06	272 ± 1031		
	Co-C[a]	2	2.94 ± 0.14	999 ± 1613		
	Co-Co	1	3.05 ± 0.02	573 ± 172		
	Co-C[a]	2	3.10 ± 0.14	999 ± 1613		
**3**	Co-N	1	1.99 ± 0.02	241 ± 287	-9.2 ± 3.0	0.026
	Co-P[a]	1	2.14 ± 0.03	507 ± 184		
	Co-Si[a]	1	2.20 ± 0.03	507 ± 184		
	Co-Si[a]	1	2.31 ± 0.03	507 ± 184		
	Co-Co	1	2.51 ± 0.01	448 ± 65		
	Co-C	3	2.88 ± 0.04	867 ± 433		
	Co-Co-P	2	4.69 ± 0.03	695 ± 268		
**4**	Co-N	1	2.00 ± 0.05	317 ± 550	-9.2 ± 3.7	0.131
	Co-P[a]	1	2.16 ± 0.04	516 ± 332		
	Co-Si[a]	1	2.24 ± 0.04	516 ± 332		
	Co-Si[a]	1	2.38 ± 0.04	516 ± 332		
	Co-Co	1	2.49 ± 0.01	155 ± 69		
	Co-C[b]	4	2.90 ± 0.06	642 ± 490		
	Co-N-C[b]	4	3.02 ± 0.06	642 ± 490		
	Co-C[c]	1	3.30 ± 0.05	61 ± 456		
	Co-C[c]	3	3.43 ± 0.05	61 ± 456		
	Co-C[c]	2	3.55 ± 0.05	61 ± 456		
	Co-P-C[c]	8	3.67 ± 0.05	61 ± 456		
	Co-C[d]	2	4.27 ± 0.04	48 ± 391		
	Co-C-C[d]	4	4.30 ± 0.04	48 ± 391		
	Co-Co-P[a]	2	4.66 ± 0.04	516 ± 332		

[a],[b],[c],[d]Bond deviation from guess value and σ^2 forced to be the same value for these paths.

EXAFS analysis of complexes 1 and 2

Geometric structure was characterized by modeling the Co K-edge EXAFS (Figures 3b, S34). The EXAFS Fourier transforms for **1** and **2** show intensity between 1-2 Å (not phase-shift corrected) resulting from single scattering contributions from Co-N, Co-P, and Co-Cl, as well as Co-O for **2**. Compared to **1**, the average Co center in compound **2** has a higher number of O/N scatterers (0.5 in **1** vs. 2 in **2**) at a relatively closer distance and a lower number of Cl/P scatterers (3.5 in **1** vs. 2 in **2**) at a farther distance. This difference results in an increase in relative scattering intensity of the feature at ~1.8 Å compared to the feature at ~1.4 Å in the phase-uncorrected Fourier transform spectra of **1** versus **2**. These observations are reinforced by the best-fit EXAFS models (Table S2). Going from **1** to **2**, the fit Co-N distance increases from 1.94 ± 0.03 Å to 2.10 ± 0.12 Å, a trend that is consistent with the crystal structures, albeit with greater uncertainty in the distance for **2** due to the similar distance to the much stronger P and Cl scatterers. Additionally, we note that the EXAFS model of **1** cannot distinguish Co-P paths from scattering with the bridging Cl ligand, due to their similar atomic number and distances within the resolution of the measurement ($\Delta R = \frac{\pi}{2\Delta k}$). Finally, a single Co-Co scattering path could be modeled from the EXAFS data, at distances of 3.85 ± 0.01 Å and 3.05 ± 0.02 Å for **1** and **2**, respectively. These distances agree well with those measured by crystallography, and overall support the assigned structures of these compounds.

References

(1) Kounalis, E.; Lutz, M.; Broere, D. L. J. Cooperative H $_2$ Activation on Dicopper(I) Facilitated by Reversible Dearomatization of an "Expanded PNNP Pincer" Ligand. *Chem. Eur. J.* **2019**, *25* (58), 13280–13284. https://doi.org/10.1002/chem.201903724.

(2) Brookhart, M.; Grant, B.; Volpe, A. F. $[(3,5\text{-}(CF_3)_2C_6H_3)_4B]\text{-}[H(OEt_2)_2]^+$: A Convenient Reagent for Generation and Stabilization of Cationic, Highly Electrophilic Organometallic Complexes. *Organometallics* **1992**, *11* (11), 3920–3922. https://doi.org/10.1021/om00059a071.

(3) Pregaglta, G.; Mazzanti, G.; Morero, D. *TETRAHYDROFURAN COMPLEXES OF TRANSITION METAL CHLORIDES*; Pergamon Press Ltd, 1962; Vol. 24.

(4) Fulmer, G. R.; Miller, A. J. M.; Sherden, N. H.; Gottlieb, H. E.; Nudelman, A.; Stoltz, B. M.; Bercaw, J. E.; Goldberg, K. I. NMR Chemical Shifts of Trace Impurities: Common Laboratory Solvents, Organics, and Gases in Deuterated Solvents Relevant to the Organometallic Chemist. *Organometallics* **2010**, *29* (9), 2176–2179. https://doi.org/10.1021/om100106e.

(5) Gaussian 16, Revision C.01, M. J. Frisch, G. W. Trucks, H. B. Schlegel, G. E. Scuseria, M. A. Robb, J. R. Cheeseman, G. Scalmani, V. Barone, G. A. Petersson, H. Nakatsuji, X. Li, M. Caricato, A. V. Marenich, J. Bloino, B. G. Janesko, R. Gomperts, B. Mennucci, H. P. Hratchian, J. V. Ortiz, A. F. Izmaylov, J. L. Sonnenberg, D. Williams-Young, F. Ding, F. Lipparini, F. Egidi, J. Goings, B. Peng, A. Petrone, T. Henderson, D. Ranasinghe, V. G. Zakrzewski, J. Gao, N. Rega, G. Zheng, W. Liang, M. Hada, M. Ehara, K. Toyota, R. Fukuda, J. Hasegawa, M. Ishida, T. Nakajima, Y. Honda, O. Kitao, H. Nakai, T. Vreven, K. Throssell, J. A. Montgomery, Jr., J. E. Peralta, F. Ogliaro, M. J. Bearpark, J. J. Heyd, E. N. Brothers, K. N. Kudin, V. N. Staroverov, T. A. Keith, R. Kobayashi, J. Normand, K. Raghavachari, A. P. Rendell, J. C. Burant, S. S. Iyengar, J. Tomasi, M. Cossi, J. M. Millam, M. Klene, C. Adamo, R. Cammi, J. W. Ochterski, R. L. Martin, K. Morokuma, O. Farkas, J. B. Foresman, and D. J. Fox, Gaussian, Inc., Wallingford CT, 2019.

(6) Lee, C.; Yang, W.; Parr, R. G. Development of the Colle-Salvetti Correlation-Energy Formula into a Functional of the Electron Density. *Phys. Rev. B* **1988**, *37* (2), 785–789. https://doi.org/10.1103/PhysRevB.37.785.

(7) Becke, A. D. Density-Functional Thermochemistry. III. The Role of Exact Exchange. *J. Chem. Phys.* **1993**, *98* (7), 5648–5652. https://doi.org/10.1063/1.464913.

(8) Weigend, F.; Ahlrichs, R. Balanced Basis Sets of Split Valence, Triple Zeta Valence and Quadruple Zeta Valence Quality for H to Rn: Design and Assessment of Accuracy. *Phys. Chem. Chem. Phys.* **2005**, *7* (18), 3297. https://doi.org/10.1039/b508541a.

(9) Grimme, S.; Ehrlich, S.; Goerigk, L. Effect of the Damping Function in Dispersion Corrected Density Functional Theory. *J. Comput. Chem.* **2011**, *32* (7), 1456–1465. https://doi.org/10.1002/jcc.21759.

(10) Grimme, S.; Antony, J.; Ehrlich, S.; Krieg, H. A Consistent and Accurate Ab Initio Parametrization of Density Functional Dispersion Correction (DFT-D) for the 94 Elements H-Pu. *J. Chem. Phys.* **2010**, *132* (15), 154104. https://doi.org/10.1063/1.3382344.

(11) Lu, T.; Chen, F. Multiwfn: A Multifunctional Wavefunction Analyzer. *J. Comput. Chem.* **2012**, *33* (5), 580–592. https://doi.org/10.1002/jcc.22885.

(12) Jmol: An Open-Source Java Viewer for Chemical Structures in 3D. Http://Www.Jmol.Org/ .

(13) NBO 6.0. E. D. Glendening, J. K. Badenhoop, A. E. Reed, J. E. Carpenter, J. A. Bohmann, C. M. Morales, C. R. Landis, and F. Weinhold (Theoretical Chemistry Institute, University of Wisconsin, Madison, WI, 2013).

(14) Bienenmann, R. L. M.; Schanz, A. J.; Ooms, P. L.; Lutz, M.; Broere, D. L. J. A Well-Defined Anionic Dicopper(I) Monohydride Complex That Reacts like a Cluster. *Angew. Chem. Int. Ed.* **2022**, *61* (29), e202202318. https://doi.org/10.1002/anie.202202318.

(15) Asundi, A. S.; Bienenmann, R. L. M.; Broere, D. L. J.; Sarangi, R. X-ray Spectroscopy Characterization of Electronic Structure and Metal-Metal Bonding in Dicobalt Complexes. *ChemRxiv (preprint)*, **2024**, https://doi.org/10.26434/chemrxiv-2024-dmm7x, accessed on 29-08-2024.

(16) Schreurs, A. M. M.; Xian, X.; Kroon-Batenburg, L. M. J. *EVAL15* : A Diffraction Data Integration Method Based on *Ab Initio* Predicted Profiles. *J. Appl. Crystallogr.* **2010**, *43* (1), 70–82. https://doi.org/10.1107/S0021889809043234.

(17) Sevvana, M.; Ruf, M.; Uson, I.; Sheldrick, G. M.; Herbst-Irmer, R. Non-Merohedral Twinning: From Minerals to Proteins Sevvana Madhumati. *Acta Crystallogr. D Struct. Biol.* **2019**, *75*, 1040–1050. https://doi.org/10.1107/S2059798319010179.

(18) Sheldrick, G. M. *SHELXT* – Integrated Space-Group and Crystal-Structure Determination. *Acta Crystallogr. A Found. Adv.* **2015**, *71* (1), 3–8. https://doi.org/10.1107/S2053273314026370.

(19) Sheldrick, G. M. Crystal Structure Refinement with *SHELXL*. *Acta Crystallogr. C Struct. Chem.* **2015**, *71* (1), 3–8. https://doi.org/10.1107/S2053229614024218.

(20) Herbst-Irmer, R.; Sheldrick, G. M. Refinement of Twinned Structures with *SHELXL* 97. *Acta Crystallogr. B* **1998**, *54* (4), 443–449. https://doi.org/10.1107/S0108768197018454.

(21) Spek, A. L. Structure Validation in Chemical Crystallography. *Acta Crystallogr. D Biol. Crystallogr.* **2009**, *65* (2), 148–155. https://doi.org/10.1107/S090744490804362X.

(22) Krause, L.; Herbst-Irmer, R.; Sheldrick, G. M.; Stalke, D. Comparison of Silver and Molybdenum Microfocus X-Ray Sources for Single-Crystal Structure Determination. *J. Appl. Crystallogr.* **2015**, *48* (1), 3–10. https://doi.org/10.1107/S1600576714022985.

(23) Ravel, B.; Newville, M. *ATHENA* , *ARTEMIS* , *HEPHAESTUS* : Data Analysis for X-Ray Absorption Spectroscopy Using *IFEFFIT*. *J. Synchrotron Radiat.* **2005**, *12* (4), 537–541. https://doi.org/10.1107/S0909049505012719.

(24) Tenderholt, A.; Hedman, B.; Hodgson, K. O. PySpline: A Modern, Cross-Platform Program for the Processing of Raw Averaged XAS Edge and EXAFS Data. In *AIP Conference Proceedings*; AIP, 2007; pp 105–107. https://doi.org/10.1063/1.2644442.

(25) Booth, C. H.; Hu, Y.-J. Confirmation of Standard Error Analysis Techniques Applied to EXAFS Using Simulations. *J. Phys. Conf. Ser.* **2009**, *190*, 012028. https://doi.org/10.1088/1742-6596/190/1/012028.
